# Preclinical models for prediction of immunotherapy outcomes and immune evasion mechanisms in genetically heterogeneous multiple myeloma

**DOI:** 10.1038/s41591-022-02178-3

**Published:** 2023-03-16

**Authors:** Marta Larrayoz, Maria J. Garcia-Barchino, Jon Celay, Amaia Etxebeste, Maddalen Jimenez, Cristina Perez, Raquel Ordoñez, Cesar Cobaleda, Cirino Botta, Vicente Fresquet, Sergio Roa, Ibai Goicoechea, Catarina Maia, Miren Lasaga, Marta Chesi, P. Leif Bergsagel, Maria J. Larrayoz, Maria J. Calasanz, Elena Campos-Sanchez, Jorge Martinez-Cano, Carlos Panizo, Paula Rodriguez-Otero, Silvestre Vicent, Giovanna Roncador, Patricia Gonzalez, Satoru Takahashi, Samuel G. Katz, Loren D. Walensky, Shannon M. Ruppert, Elisabeth A. Lasater, Maria Amann, Teresa Lozano, Diana Llopiz, Pablo Sarobe, Juan J. Lasarte, Nuria Planell, David Gomez-Cabrero, Olga Kudryashova, Anna Kurilovich, Maria V. Revuelta, Leandro Cerchietti, Xabier Agirre, Jesus San Miguel, Bruno Paiva, Felipe Prosper, Jose A. Martinez-Climent

**Affiliations:** 1 Division of Hemato-Oncology, Center for Applied Medical Research CIMA, Cancer Center University of Navarra (CCUN), Navarra Institute for Health Research (IDISNA), CIBERONC, Pamplona, Spain; 2 Immune System Development and Function Unit, Centro de Biologia Molecular Severo Ochoa, Consejo Superior de Investigaciones Cientificas/Universidad Autonoma, Madrid, Spain; 3 Department of Medicine, Mayo Clinic Arizona, Scottsdale, AZ USA; 4 Department of Hematology, Clinica Universidad de Navarra, CCUN, IDISNA, CIBERONC, Pamplona, Spain; 5 Program in Solid Tumors, Center for Applied Medical Research CIMA, University of Navarra, IDISNA, CIBERONC, Pamplona, Spain; 6 Monoclonal Antibodies Unit, Biotechnology Program, Spanish National Cancer Research Centre CNIO, Madrid, Spain; 7 Department of Anatomy and Embryology, Faculty of Medicine, University of Tsukuba, Tsukuba, Japan; 8 Department of Pathology, Yale School of Medicine, New Haven, CT USA; 9 Department of Pediatric Oncology and Program in Cancer Chemical Biology, Dana-Farber Cancer Institute, Harvard Medical School, Boston, MA USA; 10 Oncology Biomarker Development, Genentech, South San Francisco, CA USA; 11 Department of Translational Oncology, Genentech, South San Francisco, CA USA; 12 Roche Innovation Center Zurich, Roche Pharmaceutical Research and Early Development (pRED), Schlieren, Switzerland; 13 Program of Immunology and Immunotherapy, Center for Applied Medical Research CIMA, University of Navarra, IDISNA, CIBEREHD, Pamplona, Spain; 14 Translational Bioinformatics Unit, Navarra-Biomed, Public University of Navarra, IDISNA, Pamplona, Spain; 15 Biological and Environmental Sciences & Engineering Division, King Abdullah University of Science & Technology, Thuwal, Kingdom of Saudi Arabia; 16 BostonGene, Waltham, MA USA; 17 Department of Medicine, Division of Hematology and Medical Oncology, Weill Cornell Medicine, New York, NY USA; 18 Present Address: Department of Health Promotion, Mother and Child Care, Internal Medicine and Medical Specialties, University of Palermo, Palermo, Italy

**Keywords:** Myeloma, Myeloma, Immune evasion

## Abstract

The historical lack of preclinical models reflecting the genetic heterogeneity of multiple myeloma (MM) hampers the advance of therapeutic discoveries. To circumvent this limitation, we screened mice engineered to carry eight MM lesions (NF-κB, KRAS, MYC, TP53, BCL2, cyclin D1, MMSET/NSD2 and c-MAF) combinatorially activated in B lymphocytes following T cell-driven immunization. Fifteen genetically diverse models developed bone marrow (BM) tumors fulfilling MM pathogenesis. Integrative analyses of ∼500 mice and ∼1,000 patients revealed a common MAPK–MYC genetic pathway that accelerated time to progression from precursor states across genetically heterogeneous MM. MYC-dependent time to progression conditioned immune evasion mechanisms that remodeled the BM microenvironment differently. Rapid MYC-driven progressors exhibited a high number of activated/exhausted CD8^+^ T cells with reduced immunosuppressive regulatory T (T_reg_) cells, while late MYC acquisition in slow progressors was associated with lower CD8^+^ T cell infiltration and more abundant T_reg_ cells. Single-cell transcriptomics and functional assays defined a high ratio of CD8^+^ T cells versus T_reg_ cells as a predictor of response to immune checkpoint blockade (ICB). In clinical series, high CD8^+^ T/T_reg_ cell ratios underlie early progression in untreated smoldering MM, and correlated with early relapse in newly diagnosed patients with MM under Len/Dex therapy. In ICB-refractory MM models, increasing CD8^+^ T cell cytotoxicity or depleting T_reg_ cells reversed immunotherapy resistance and yielded prolonged MM control. Our experimental models enable the correlation of MM genetic and immunological traits with preclinical therapy responses, which may inform the next-generation immunotherapy trials.

## Abstract (web summary)

New experimental models provide much-needed tools for understanding how genetically diverse multiple myeloma progresses and evolves in response to therapy.

## Main

MM is a neoplasia of bone marrow (BM) plasma cells (PCs), which secrete monoclonal immunoglobulins that induce multi-organ damage^1^. MM occurs predominantly in older people, and is preceded by an asymptomatic condition termed monoclonal gammopathy of undetermined significance (MGUS)^2,3^. Progression of MGUS into MM usually proceeds through a transitional stage known as smoldering multiple myeloma (SMM). Understanding the mechanisms driving progression from precursor conditions into clinically active MM may contribute to the implementation of early therapies for select groups of individuals^1^.

Genetic heterogeneity is a hallmark of MM^4^. Chromosomal translocations of immunoglobulin-coding genes and hyperdiploidy are considered early genetic events, being followed by abnormalities in NF-κB, MAPK–RAS and apoptotic pathways that promote the full malignant MM phenotype^4,5^. Late-stage genetic changes frequently involve *MYC* and *TP53* genes, which are commonly altered in relapsed/refractory MM^6,7^. Based on genetic features, MM is classified into risk groups that exhibit different outcomes to standard-of-care therapies^4,5^. In this scenario, the order of acquisition of the primary genetic lesions, and how they contribute to MM progression from precursor states, have not been completely elucidated^5^. Beyond genetics, accumulating evidence indicates that survival of neoplastic PCs largely depends on the interplay with the BM hematopoietic cell niche where they reside^8^. Thus, a tumor suppressive microenvironment provides effective surveillance to restrict PC growth at the MGUS and SMM stages, while progressive immuno-editing leading to T cell exhaustion underlies MM transformation^2,3,8,9^. However, the mechanisms by which genetically diverse tumor cells interact with the BM microenvironment to evade immunological surveillance during progression are largely unknown.

Addressing these scientific questions is of clinical relevance, because despite continuous improvement in MM survival, a cure remains elusive and the majority of individuals with MM eventually relapse^1^. Novel immunotherapy strategies with monoclonal antibodies, T cell engagers and chimeric antigen receptor T cell therapies hold promise for more prolonged MM control, which might eventually lead to a cure^10–14^. However, such therapeutic efficacy clearly contrasts with the low response rate of patients with MM to immune checkpoint inhibitors^15,16^. Deciphering the mechanisms that underlie the discrepant outcomes to different immunotherapeutic approaches is urgently required. However, this investigation is seriously hampered by the paucity of experimental mouse models recapitulating the principal clinical, genetic and immunological characteristics of MM^17–22^. In this setting, a major obstacle to generating MM in mice has been the uncertainty about the disease’s cell of origin and the key genetic drivers that initiate and sustain the transformation process. The lack of mouse models of MM restricts preclinical immunotherapy research, which constitutes a current unmet medical need.

Here, we introduce fifteen genetically engineered mouse models of human-like MM that reflect the key elements in the pathogenesis of the disease: the genetic heterogeneity, the progressive transition of MGUS and SMM states into clinical active disease, and the interaction of tumor cells with the BM immune microenvironment during transformation. Our results point to MYC as a key regulator of the tumor and immune progression in genetically heterogeneous MM, which conditions clinical responses to immunotherapy.

## Results

### Modeling genetic heterogeneity of human multiple myeloma in mice

**Fig. 1:**
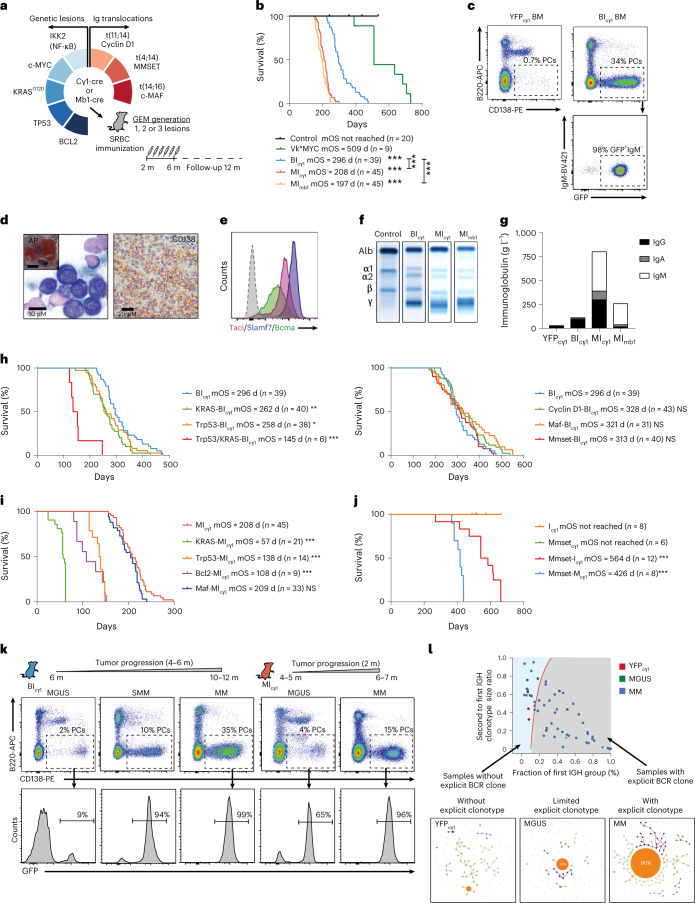
Genetically heterogeneous mouse models of human-like multiple myeloma. **a**, Schematic of the genetic screen strategy, whereby transgenic mice were crossed with cγ1-cre or mb1-cre mice. Among 31 genetically heterogeneous mouse lines generated, MI_mb1_, MI_cγ1_ and BI_cγ1_ strains developed MM. GEM, genetically engineered mice; m, months. **b**, Kaplan–Meier OS curves of MI_mb1_, MI_cγ1_, BI_cγ1_, control (YFP_cγ1_ and YFP_mb1_) and Vk*MYC mice. **c**, Representative flow cytometry analysis in the BM of BI_cγ1_ mice at the time of death, which shows an increased number of GFP^+^CD138^+^B220^−^sIgM^−^ MM cells. **d**, Giemsa staining of a representative BM sample in BI_cγ1_ mice revealed human-like PCs with expression of acid phosphatase (AP; left). On the right, immunohistochemical examination in BI_cγ1_ mice revealed CD138 surface expression by MM cells. **e**, MM cells show increased surface expression of Bcma, Slamf7 and Taci according to flow cytometry analyses. **f**, Representative electrophoresis of immunoglobulin secretion in serum samples from MI_mb1_, MI_cγ1_ and BI_cγ1_ mice shows M spikes corresponding to the gamma fraction. **g**, Quantification of immunoglobulin isotypes in serum samples by ELISA in MI_mb1_ (*n* = 3), MI_cγ1_ (*n* = 2), BI_cγ1_ (*n* = 4) and YFP_cγ1_ control (*n* = 9) mice. **h**, Kaplan–Meier survival curves of mouse lines that develop MM derived from the BI_cγ1_ strain with additional *KRAS*^*G12D*^ mutation, heterozygous *Trp53* deletion, or expression of cyclin D1, c-MAF or MMSET. **i**, Kaplan–Meier survival curves of mouse lines that develop MM derived from MI_cγ1_ mice with additional *KRAS*^*G12D*^ mutation, heterozygous *Trp53* deletion, c-MAF expression or BCL2 expression. **j**, Kaplan–Meier survival curves in mice with MMSET/NSD2 expression crossed with lines carrying either *IKK2*^*NF-κB*^ activation or c-MYC expression, which developed MM at old ages. **k**, Flow cytometry analyses in BI_cγ1_ and MI_cγ1_ mice revealed that precursor states precede clinically evident MM in genetically heterogeneous mice. **l**, Analysis of *Igh* clonality according to RNA-seq of immunoglobulin gene loci and classification by the presence of explicit clonotypes for each sample. B cell receptor (BCR) repertoires and the most expanded clone groups in control, MGUS and MM samples. Log-rank (Mantel–Cox) test was used. *P < 0.05; **P < 0.01; ***P < 0.001; NS, not significant. Source data

**Extended Data Fig. 1:**
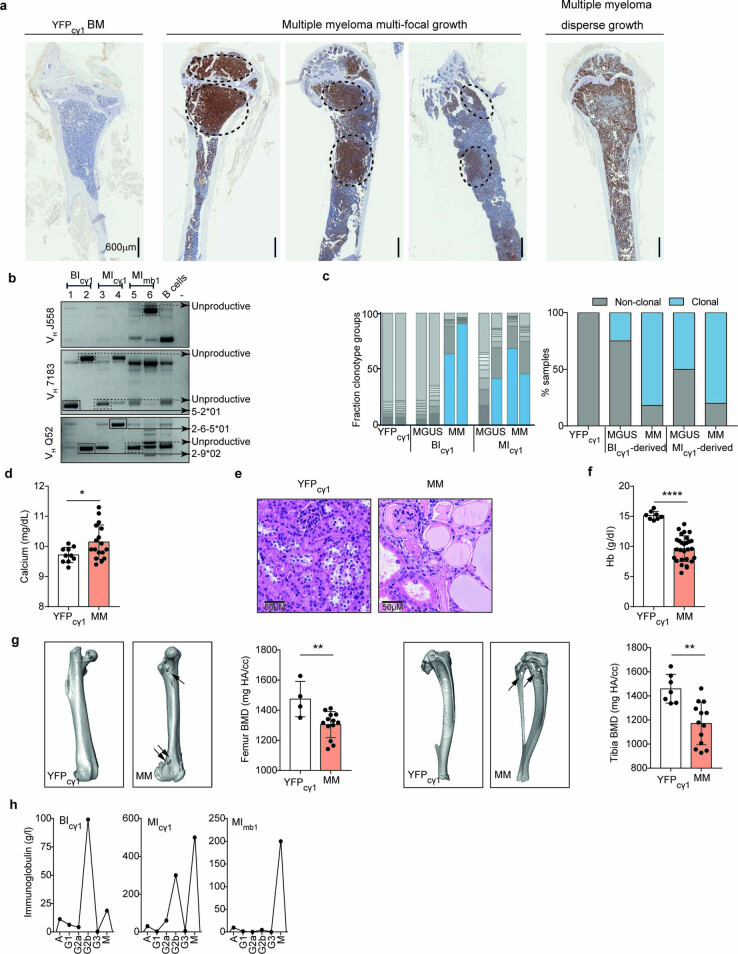
Characterization of multiple myeloma in genetically engineered mice. a) Immunohistochemical analysis of bone sections using GFP staining to visualize the GFP^+^ MM cells within the BM (excluding by-stander GFP-negative PCs). Two MI_cγ1_ mice, two BI_cγ1_ mice and one YFP_cγ1_ control mice were characterized. A multifocal growth of MM is observed in three of the four examined mice, including focal lesions in the BM. b) Examination of tumor clonality by genomic PCR and sequencing revealed clonal *IghV* gene rearrangements in DNA isolated from BM PCs from two MI_mb1_, two MI_cγ1_ and two BI_cγ1_ mice. As negative control, splenic B220^+^ B cells from a YFP_cγ1_ mouse were included. c) Representation of the fraction clonotype groups according to the tumor *IghV* gene clonality in two samples from BI_cγ1_ and MI_cγ1_ mice at MGUS and MM states, shown on the left. The percentage of samples with clonal and non-clonal *IghV* genes in BI_cγ1_-derived and MI_cγ1_-derived strains at MGUS and MM states is shown on the right. Representation of CRAB features in MI_mb1_, MI_cγ1_, and BI_cγ1_ mice (n = 17-28), including hypercalcemia (d), renal disease due to Ig light-chain deposits in tubules (e), anemia (f), and bone disease (g). In g), presentative images of micro-computed tomography (micro-CT) performed in the bones of mice with MM are shown, which detected osteolytic lesions (marked with arrows) in femur (left) and tibia and fibula (right) in BI_cγ1_ and MI_cγ1_ mice, respectively. As controls, YFP_cγ1_ mice were characterized, which did not show bone lesions. In addition, quantification of bone density from micro-CT images was performed in 13 mice from different genotypes at the MM stage, which showed global decrease of bone mineral density (BMD) in femur (left) and tibia (right) with respect to controls(n = 4-7). Bars represent mean ± s.d. Unpaired two-tailed t Student test or Mann-Whitney test P values (d, f and g) are indicated. h) Representative examples of individual mice showing the quantification of Ig isotypes in serum samples by ELISA. *p < 0.05; **p < 0.01; ***p < 0.001; NS, non-significant.

To establish preclinical models of genetically diverse MM, transgenic mice carrying eight MM genetic drivers that recapitulate the most common changes observed in human MM were bred to engineer strains with single, double and triple genetic alterations. These included NF-κB signaling activation by IKBKB/IKK2 expression, a KRAS^G12D^ mutation, antiapoptotic BCL2 expression, c-MYC expression, TP53 deletion, and constitutive expression of cyclin D1, c-MAF and MMSET/NSD2 mimicking immunoglobulin translocations t(11;14), t(16;14) and t(4;14), respectively (Supplementary Table 1)^4,5^. These changes were triggered in immature pre-B lymphocytes or mature germinal center (GC) B lymphocytes, which are the two developmental stages proposed to be the origin of the disease^23,24^, using mb1-cre or cγ1-cre mice, respectively^25,26^. Young mice were immunized with sheep red blood cells (SRBCs) to induce the formation of PCs labeled with a GFP reporter, after which mice were monitored for MM development up to 12 months of age (Fig. 1a, Methods and Supplementary Fig. 1). Vk*MYC mice were included as a reference model of MM development at a late age, driven by single MYC expression in GC B lymphocytes^17^. Among 31 strains bearing varied genetic combinations, 9 developed lethal tumors classified as mature B cell lymphoma or acute lymphoblastic leukemia (Supplementary Fig. 2a–c). Three of the remaining lines exhibited fully penetrant PC tumors in the BM, which shortened median overall survival (mOS) to below 12 months of age (Fig. 1b). Two of these mouse lines were termed MI_mb1_ and MI_cγ1_ as they carry MYC and IKK2^NF-κB^ expression by mb1-cre or cγ1-cre alleles, respectively, which indicates that NF-κB activation accelerated MYC-driven MM development in these two models compared to Vk*MYC mice (mOS, 197 d and 208 d versus 509 d; *P* < 0.001). The third mouse line was termed BI_cγ1_ as it carries BCL2 and IKK2^NF-κB^ expression by the cγ1-cre allele, and exhibited an mOS of 296 d, which indicates that apoptosis blockade in cells with NF-κB signaling was sufficient for transformation (Supplementary Table 1). BM tumors in the three different lines were composed of >10% GFP^+^CD138^+^B220^−^sIgM^−^ PCs, which morphologically resembled human MM cells and exhibited a multifocal infiltration pattern in the BM; they also expressed typical MM markers including acid phosphatase, Bcma, Slamf7 and Taci, secreted immunoglobulins into the serum, and showed clonal *IghV* gene rearrangements (Fig. 1c–f and Extended Data Fig. 1a–c). In addition, mice presented with common CRAB-like clinical features (hyperCalcemia, Renal disease, Anemia and Bone disease; Extended Data Fig. 1d–g). However, while the BI_cγ1_ and MI_cγ1_ strains predominantly secreted IgG or IgA, the MI_mb1_ mice derived from immature pre-B cells presented IgM-secreting MM (Fig. 1g and Extended Data Fig. 1h). Genetic studies in patients with IgM MM, corresponding to less than 1% of MM cases, showed a pre-germinal B lymphocyte origin, which is matched by the MI_mb1_ model^27^. In contrast, BI_cγ1_ and MI_cγ1_ mice developed class-switched MM from GC B lymphocytes that fulfill the diagnostic criteria of human disease, which implicates these cells in the origin of typical MM.

**Extended Data Fig. 2:**
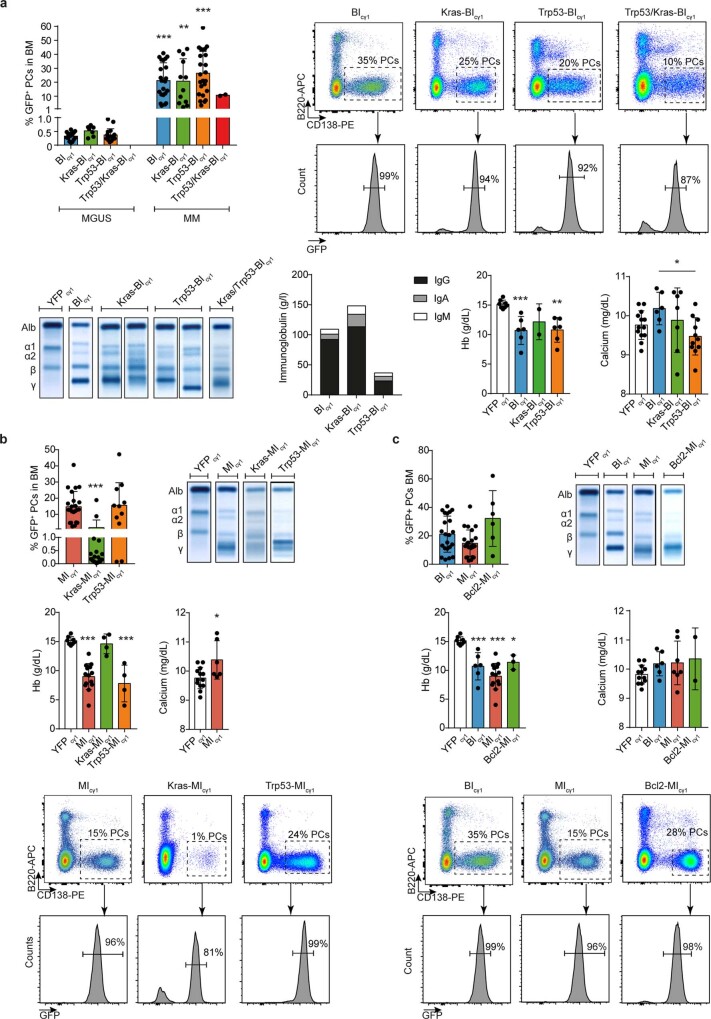
Characterization of BI_cγ1_ and MI_cγ1_ models with additional genetic lessions. MM development in BI_cγ1_ (a) and MI_cγ1_ (b) strains carrying an additional *KRAS*^*G12D*^ mutation or heterogeneous deletion of *Trp53* (complementary to Fig. 1h–i). In c), characterization of MM development in MI_cγ1_ mice with additional expression of BCL2 (Bcl2-MI_cγ1_ mice) (complementary to Fig. 1i). Data is depicted as mean ± s.d. P values are obtained using one-way ANOVA test followed by Tukey’s multiple comparison test (a, b and c), Kruskal-Wallis adjusted for multiple comparisons by Dunn’s test (b and c), unpaired t test (a and b) and Mann-Whitney test (a). Source data

**Extended Data Fig. 3:**
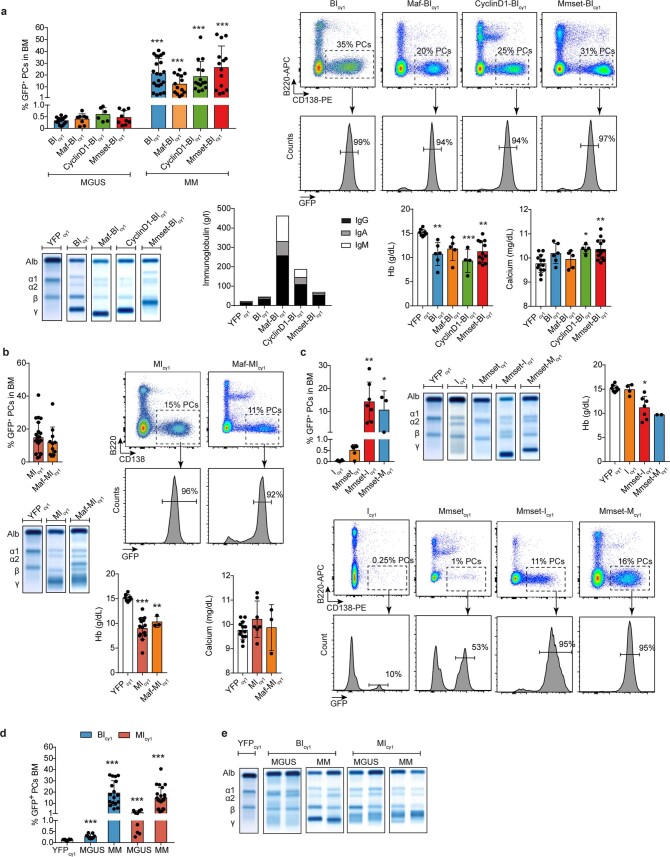
Characterization of BI_cγ1_ and MI_cγ1_ mice with additional immunoglobulin chromosomal translocations. MM development in BI_cγ1_ (a) and MI_cγ1_ (b) mice carrying immunoglobulin chromosomal translocations (complementary to Fig. 1h-i). c) Characterization of MM development in mice with t(4;14) crossed with lines carrying IKK2^NF-κB^ activation or MYC expression (complementary to Fig. 1j). d) Quantification of GFP^+^CD138^+^B220^−^sIgM^−^ PCs by flow cytometry in the BM of BI_cγ1_ and MI_cγ1_ mice at MGUS and MM states, and in YFP_cγ1_ control mice at 6 months of age, are shown (complementary to Fig. 1k). e) Representative electrophoresis analyses of immunoglobulin secretion in serum samples from BI_cγ1_ and MI_cγ1_ mice at MGUS and MM states, and in YFP_cγ1_ control mice, are shown. Data is depicted as mean ± s.d.. P values are obtained using one-way ANOVA test followed by Tukey’s multiple comparison test (a and b), Kruskal-Wallis adjusted for multiple comparisons by Dunn’s test (c and d), unpaired t test (a) and Mann-Whitney test (a and b). Source data

To build MM genetic heterogeneity, BI_cγ1_ and MI_cγ1_ strains were crossed with lines carrying additional MM genetic changes, including the common *KRAS*^*G12D*^ mutation and the high-risk *Trp53* deletion^4^. Both genetic abnormalities shortened the time to MM development in BI_cγ1_ and MI_cγ1_ mice, inducing a BM disease composed of GFP^+^CD138^+^B220^−^sIgM^−^ PCs that secreted IgG or IgA, and was classified as MM (Fig. 1h,i and Extended Data Fig. 2a,b). Likewise, concomitant *KRAS*^*G12D*^ and *Trp53* deletion in BI_cγ1_ mice rapidly induced BM and extramedullary PC tumors (Extended Data Fig. 2a). These experimental results mimic data from individuals with SMM^28,29^, which indicates that MAPK–RAS mutations and heterozygous *TP53* inactivation accelerate the onset of clinically active MM from precursor conditions. Then we explored whether apoptosis restriction could influence MM development in MI_cγ1_ mice. To this end, transgenic BCL2 expression was added to the MI_cγ1_ strain, which yielded marked acceleration of MM onset (Fig. 1i and Extended Data Fig. 2c). We further expanded the genetic heterogeneity by adding the overexpression of the three genes involved in the immunoglobulin chromosomal translocations used to stratify MM into genetic-risk groups^4,5^. To achieve this, BI_cγ1_ mice were crossed with the Eµ-cyclin D1, Eµ-MAF or the newly generated Rosa26-hMMSET-IIStop-floxed mouse lines, representative of standard-risk t(11;14) or the high-risk t(14;16) and t(4;14) translocations, respectively. BI_cγ1_ mice carrying overexpression of each of these three transgenes developed BM tumors classified as typical MM, all of which exhibited overlapping survival curves (Fig. 1h and Extended Data Fig. 3a). Similarly, a strain derived from MI_cγ1_ mice with additional overexpression of c-MAF developed MM and exhibited a similar survival to that of MI_cγ1_ mice (Fig. 1i and Extended Data Fig. 3b). Likewise, SMM patients carrying t(11;14), t(14;16) or t(4;14) are not at increased risk of progression to active MM compared with those without immunoglobulin translocations^28,29^. On the other hand, dysregulation of MMSET contributed to MM initiation, as MMSET transgenic mice crossed with lines carrying either IKK2^NF-κB^ activation or MYC expression drove MM development (Fig. 1j and Extended Data Fig. 3c). These experimental findings suggest that the expression of the oncogenes involved in the immunoglobulin translocations contributes to MM development, while such additional expression does not accelerate MM onset. Taken together, we have developed a panel of 15 mouse models encompassing MM genetic heterogeneity, including the standard-risk and high-risk genetic subgroups (Extended Data Table 1).

### Multiple myeloma is preceded by MGUS and SMM-like precursor states

We next determined whether, like in humans, precursor disease was present before the onset of symptomatic MM^2,3^. In BI_cγ1_ and BI_cγ1_-derived mice, lethal MM was uniformly preceded by an MGUS-like stage from 6 months of age, characterized by minimal BM infiltration of oligoclonal GFP^+^CD138^+^B220^−^sIgM^−^ PCs that moderately secreted class-switched immunoglobulins into the serum (Fig. 1k,l and Extended Data Fig. 3d,e). The number of PCs, the degree of *IghV* clonality, and the levels of immunoglobulins increased over time and demarcated an SMM-like asymptomatic stage with >10% of clonal PCs, which eventually transformed into MM in 4 to 6 months. In contrast, MI_cγ1_ and MI_cγ1_-derived mice exhibited prominent MGUS-like disease in BM from 4–5 months of age that rapidly transformed into aggressive MM within several weeks (Fig. 1k,l, Extended Data Fig. 3d,e and Supplementary Fig. 2d). Thus, pre-malignant stages precede clinically evident MM in genetically heterogeneous mice. However, MI_cγ1_-derived models exhibited a rapid MGUS-to-MM transition, while the BI_cγ1_-derived strains were characterized by a longer time to progression, which in humans corresponds to the many years required by MGUS cells undergoing MM transformation^2,3^. In summary, our genetically diverse mice recapitulate the natural history and clinical evolution of human disease, including models of early and late MM progression from precursor states.

### MYC activation is a common feature in multiple myeloma genetic groups

**Fig. 2:**
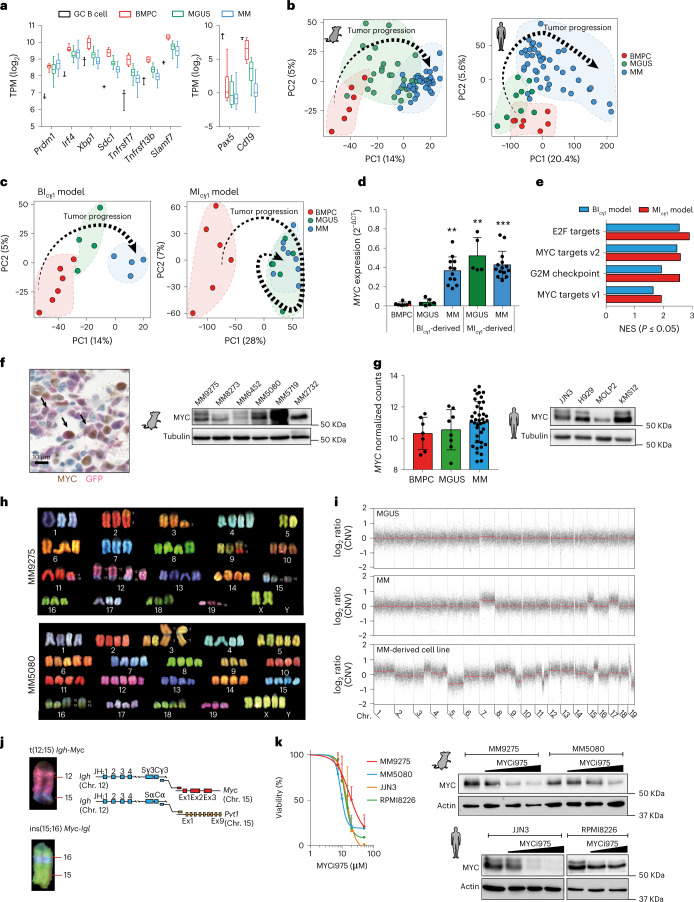
Transcriptional and genomic profiling of multiple myeloma in mice. **a**, RNA-seq analyses of typical PC and B cell genes in PCs from mice at MGUS (*n* = 25) and MM (*n* = 40) stages versus control BM PCs (*n* = 6) and GC B cells (*n* = 3). TPM, transcripts per million. Boxes represent the median, upper and lower quartiles and whiskers represent minimum to maximum range. **b**, PCA of RNA-seq data from mouse and human MGUS and MM cells compared with control BM PCs. Human PCs were obtained from patients with newly diagnosed MGUS (*n* = 9) and MM (*n* = 41), and from BM aspirates from healthy donors (*n* = 7). **c**, PCA of RNA-seq data from BI_cγ1_ and MI_cγ1_ mice revealed two transcriptional modes of evolution during MM development. **d**, Quantitative PCR with reverse transcription (RT–qPCR) of mouse and human *MYC* gene expression in isolated BM PCs (*n* = 7), MGUS (*n* = 6) and MM (*n* = 12) cells from BI_cγ1_-derived and MGUS (*n* = 5) and MM (*n* = 14) cells from MI_cγ1_-derived mice. The mean and s.d. are represented. Kruskal–Wallis test *P* values adjusted for multiple comparisons by Dunn’s test are indicated. **e**, GSEA of RNA-seq data shows ‘MYC target genes’ at the top of the MM hallmarks in BI_cγ1_-related and MI_cγ1_ mice. NES, normalized enrichment score. **f**, Immunohistochemical image of BM sections revealed nuclear MYC protein expression in GFP^+^ MM cells from BI_cγ1_ mice (left). Western blot analysis revealed MYC expression in mouse MM-derived cell lines (right). **g**, *MYC* expression from RNA-seq data in samples from patients with MGUS (*n* = 8) or MM (*n* = 39) and in BM PCs (*n* = 7) from healthy donors. The mean ± s.d. is represented. Western blot analysis of MYC protein expression in human MM cell lines (right). **h**, Representative examples of spectral karyotyping analysis in metaphase cells from two MM-derived cell lines. **i**, Copy number variation and WES analyses of primary cells from mice with MGUS and MM and in an MM-derived cell line. **j**, WGS mapped the breakpoints in two chromosomal translocations between the *Igh* or *Igl* and *MYC* genes in MM9275 and MM5080 cell lines, respectively. **k**, MYC targeting with the MYC inhibitor MYCi975 reduced MYC expression (right) and decreased MM cell viability (left) in mouse and human MM cells. Data corresponding to the mean ± s.e.m. from two or three independent experiments are represented for each cell line. *P < 0.05; **P < 0.01; ***P < 0.001. Source data

RNA sequencing (RNA-seq) of MGUS and MM cells from MI_cγ1_-derived and BI_cγ1_-derived mice defined a common transcriptional signature with respect to normal BM PCs, including the upregulation of PC genes (that is, *Prdm1*, *Irf4*, *Xbp1*, *Sdc1* encoding Cd138, *Tnfrsf17* encoding Bcma, *Tnfrsf13b* encoding Taci and *Slamf7*) and the downregulation of B cell genes (that is, *Pax5* and *Cd19*; Fig. 2a and Supplementary Table 2). To compare mouse tumors with human disease, RNA-seq was applied to malignant PCs from newly diagnosed MGUS and MM patients to define a human transcriptional signature with respect to normal BM PCs. Using principal-component analysis (PCA), mouse and human MGUS cells were mapped in between PCs and MM cells, which is indicative of a similar evolving transcriptional trajectory (Fig. 2b and Supplementary Fig. 3a,b). Additionally, gene-set enrichment analysis (GSEA) showed enrichment of transcriptionally deregulated mouse genes in the human MM expression signatures (Supplementary Fig. 3c and Supplementary Table 3). These data indicate that mouse and human MM share a common transcriptional profile. We then characterized the transcriptional changes underlying the transition of MGUS into MM in mouse models with different times to progression. BI_cγ1_-derived mice exhibited a linear transcriptional evolution as BM PCs progressed to MGUS cells and then to MM cells, concordant with the late progression. In contrast, MGUS and MM cells from MI_cγ1_ mice clustered closely and exhibited a reduced number of differentially expressed genes, concordant with the rapid progression (Fig. 2c and Supplementary Table 4). Comparative analyses of these two transcriptional patterns of progression revealed that the *MYC* oncogene was highly expressed in MM cells compared with MGUS cells in the BI_cγ1_-derived models, while transgenic *MYC* expression was already high in MGUS cells from MI_cγ1_ mice and remained stable during MM progression (Fig. 2d). GSEA of the MM transcriptomes found that ‘MYC target genes’ were among the top hallmarks in both BI_cγ1_-derived and MI_cγ1_-derived models (Fig. 2e). Accordingly, MYC protein expression was detected in primary BM GFP^+^ MM cells and MM-derived cell lines established from primary MM samples, including early and late progressors (Fig. 2f, Supplementary Fig. 4 and Supplementary Table 5). These results demonstrate the acquisition of endogenous MYC expression during MM progression in BI_cγ1_-derived models, while early activation of transgenic MYC in MI_cγ1_ mice accelerates MM progression. Likewise, in patients, *MYC* expression levels in MGUS cells were similar to those in BM PCs and were increased in MM cells (Fig. 2g), which agrees with previous studies^7,17,28,29^, and confirms that MYC regulates time to progression into MM.

Genetic characterization of mouse MM cells revealed karyotypes with triploidy, tetraploidy or complex aneuploidy, with recurrent chromosomal gains and losses as well as structural rearrangements (Fig. 2h,i). These included human-like translocations between *MYC* and the *Igh* or *Igl* genes in 11 of 62 (18%) primary MM samples and 3 of 6 (50%) MM-derived cell lines (Fig. 2j)^7^. However, *MYC* chromosomal changes were not observed in MGUS cells, indicating that these were acquired during MM progression, as reported in patients (Supplementary Fig. 5)^7,28,29^. We then evaluated the oncogenic function of MYC in genetically diverse MM-derived cell lines. Selective targeting of MYC with the small molecule MYCi975 induced dose-dependent MYC protein reduction^30,31^, which decreased viability of mouse and human MM cells (Fig. 2k). Therefore, MYC activation is a unifying feature in genetically heterogeneous MM, which distinguishes cases with early and late progression from precursor stages.

### A MAPK–MYC genetic axis is amenable to targeted therapy

**Fig. 3:**
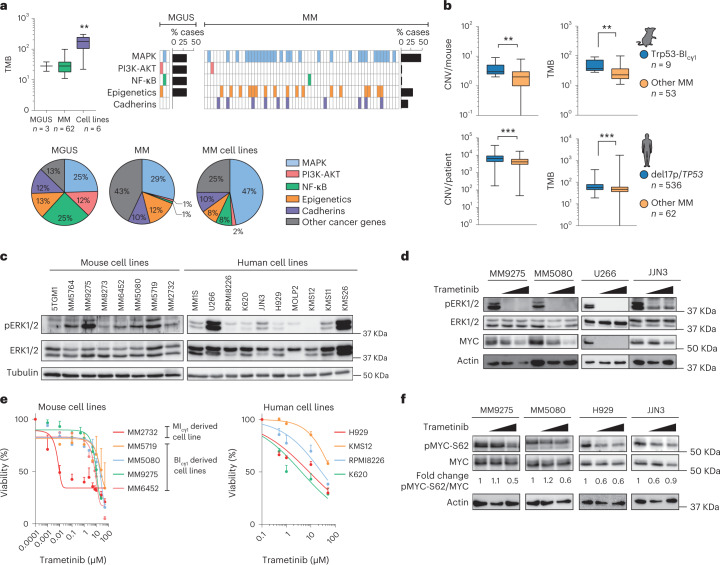
A common MAPK–MYC axis dictates multiple myeloma progression. **a**, Quantification of the TMB, which corresponds to the total number of somatic mutations per tumor, according to WES analysis (left). Distribution of mutations in genes within signaling and cancer-related pathways in MM (*n* = 62) and MGUS (*n* = 3) primary samples, and in MM-derived cell lines (*n* = 6). Kruskal–Wallis test *P* values adjusted for multiple comparisons by Dunn’s test are indicated. **b**, Quantification of copy number variation and TMB according to WES data from MM cells from Trp53-BI_cγ1_ mice compared with the remaining strains, and in MM patients from the CoMMpass study with and without 17p/*TP53* deletion and/or *TP53* somatic mutations. Mann–Whitney test two-tailed *P* values are indicated. **c**, Western blot analyses revealed ERK phosphorylation in mouse and human MM cell lines. The mouse cell line 5TGM1 was included as a positive control. **d**, The MEK inhibitor trametinib induced a dose-dependent reduction in ERK phosphorylation in mouse and human MM-derived cell lines. **e**, Dose-dependent decrease in viability of mouse and human MM cell lines following trametinib treatment. Data corresponding to the mean ± s.e.m. from two to ten independent experiments are represented for each cell line. **f**, Reduced phosphorylation of MYC at S62 (pMYC-S62) following treatment with trametinib in mouse and human MM cell lines. Quantification of the fold change in expression levels of pMYC-S62 with respect to total MYC protein is shown. Boxes represent the median, upper and lower quartiles and whiskers represent minimum to maximum range (**a** and **b**). **P < 0.01; ***P < 0.001. Source data

Quantification of the tumor mutational burden (TMB) by whole-exome sequencing (WES) revealed 28 somatic mutations in each mouse tumor in MGUS samples, 31 in MM samples and 172 in MM-derived cell lines (Supplementary Table 6). These included typical mutations in the*Tent5c* gene and in genes encoding epigenetic modulators and cadherins (Fig. 3a). Mutations in genes in the NF-κB pathway were observed in 1 of 31 MM samples, which indicates that moderate NF-κB signaling activation by transgenic *IKK2* expression in heterozygosity is enough for the development of precursor stages, which progress into MM without additional changes in the pathway. In clear contrast, mutations in genes in the MAPK pathway were observed in 29 of 62 (47%) mice at the MM stage; these rates are like those observed in MM patients^4–6^, which suggests that mutations in this signaling cascade accumulate during MM progression (Supplementary Table 7). Analysis of the genomic characteristics in MM cells from the models of early and late progression revealed that MI_cγ1_ mice exhibited normal karyotypes without *MYC* translocations, while BI_cγ1_ mice with *Trp53* deletion exhibited more abundant chromosomal abnormalities and higher TMB compared with the strains without *Trp53* deletion (Fig. 3b and Supplementary Fig. 6). Concordantly, among 599 MM patients in the CoMMpass study (NCT01454297), those carrying del(17p) and/or *TP53* mutations exhibited higher copy number changes and TMB compared with the remaining patients (Fig. 3b), indicating that TP53-driven genetic instability promotes genetic rearrangements including those involving *MYC* during MM progression.

We next asked whether the acquired MAPK mutations were analogous in mouse and human MM. Of the 34 MAPK genes found with mutations in mouse MM, 19 (56%) were recurrently mutated in MM patients in the CoMMpass study (Supplementary Table 8). Accordingly, western blot analyses identified consistent phosphorylation of the protein kinase ERK, a surrogate of MAPK activation, in mouse and human MM-derived cell lines (Fig. 3c). Moreover, targeting MAPK signaling with trametinib, a MEK-ERK inhibitor clinically approved for *BRAF*-mutated melanoma^32^, reversed ERK phosphorylation and reduced mouse and human MM cell growth, which indicates shared MAPK activation (Fig. 3d,e). Given that mutations in MAPK pathway and MYC activation are acquired during MM development in mice, we investigated whether MAPK signaling could modulate MYC expression. Although trametinib did not consistently change *MYC* gene expression at the RNA level, MEK inhibition decreased phosphorylation of MYC at Ser62, which induced dose-dependent MYC degradation (Fig. 3f)^33^. These results suggest that while *Trp53* loss triggers transcriptional *MYC* activation through chromosomal rearrangements, constitutive MAPK signaling stabilizes MYC protein during MM development. These data are in accordance with the Trp53/KRAS-BI_cγ1_ mouse model (Fig. 1h and Supplementary Fig. 4), which showed that simultaneous *Trp53* loss and KRAS^G12D^ cooperated to accelerate MM onset.

### Immunological features of the bone marrow microenvironment in multiple myeloma

**Fig. 4:**
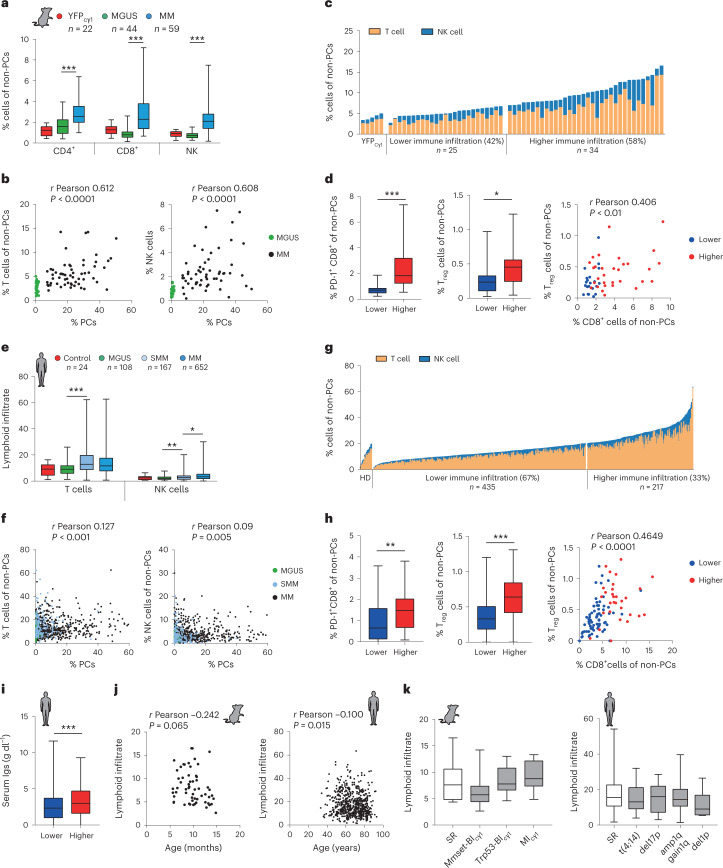
Immune features of multiple myeloma progression. **a**, Distribution of lymphoid cell subpopulations in the BM of mice with MGUS and MM, and in control mice. **b**, Two-tailed Pearson correlation analyses between the number of BM PCs in mice at MGUS and MM states with T cells or NK cells in the BM. **c**, Classification of MM samples into categories according to the abundance of T and NK lymphoid cells in the BM with respect to that in healthy mice. **d**, MM cases with higher number of infiltrating immune cells contained more tumor-reactive PD-1^+^CD8^+^ T cells and T_reg_ cells. Two-tailed Pearson correlation analysis between CD8^+^ T cells and T_reg_ cells in the BM (right). **e**, Characterization of the BM lymphoid cell composition by flow cytometry in BM samples from patients with MGUS, SMM and MM. **f**, Two-tailed Pearson correlation analyses between the percentage of PCs in the BM from MM patients and the percentage of T or NK cells in the BM. **g**, Classification of MM patients (*n* = 652) into those with lower and higher number of immune cells in the BM microenvironment with respect to healthy donors (HDs; *n* = 24). **h**, Tumors with high immune infiltrates contained more tumor-reactive PD-1^+^ CD8^+^ T cells and T_reg_ cells in the BM compared with MM cases with a lower number of immune cells. Two-tailed Pearson correlation analysis between the percentages of CD8^+^ T cells in BM and the percentage of T_reg_ cells (right). **i**, MM cases with more abundant immune cells had increased immunoglobulin secretion with respect to the remaining cases. **j**, Two-tailed Pearson correlation analyses between the T and NK lymphoid cell infiltrate in BM from mice (*n* = 59) and humans with MM (*n* = 638) and the age. **k**, Quantification of the BM lymphoid infiltrates including CD4^+^, CD8^+^ and NK cells across genetic subgroups of mouse and human MM. Boxes represent the median, upper and lower quartiles and whiskers represent minimum to maximum range (**a**, **d**, **e**, **h**, **i** and **k**). Kruskal–Wallis test *P* values adjusted for multiple comparisons by Dunn’s test (**a**, **b** and **k**) and Mann–Whitney test *P* values (**d**, **h** and **i**) are indicated. *P < 0.05; **P < 0.01; ***P < 0.001. Source data

**Extended Data Fig. 4:**
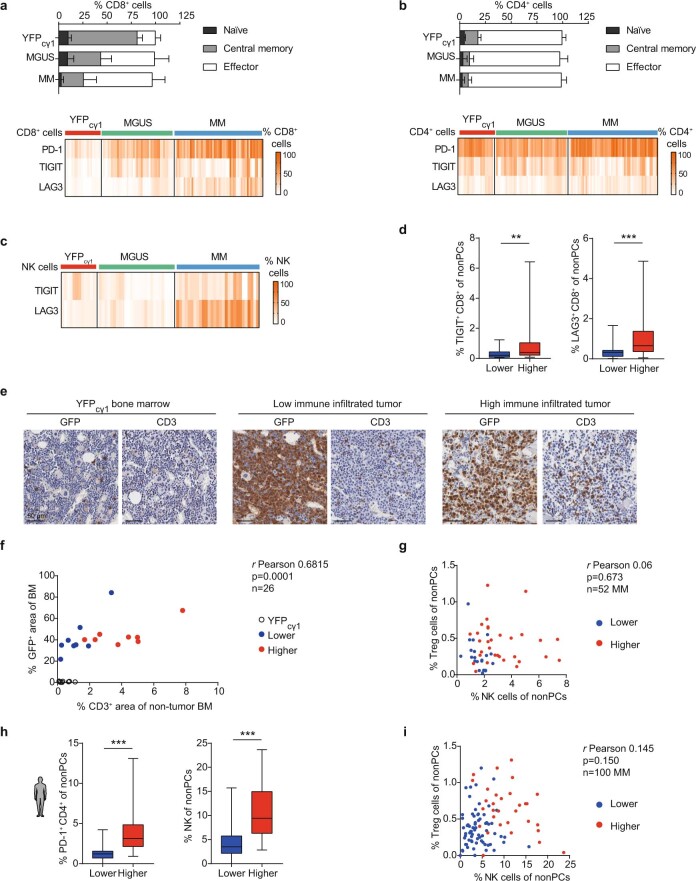
Immunological characteristics of genetically engineered mice with multiple myeloma. a) Stage of CD8^+^ T cells (a) and CD4^+^ T cells (b) during MGUS (n = 8) and MM (n = 27) progression in mice. Controls corresponded to YFP_cγ1_ mice (n = 3). Phenotype of exhaustion markers in CD8^+^ T cells (a) and in CD4^+^ T cells (b) during MGUS (n = 24) and MM (n = 43) compared with control age-matched mice (n = 17) (complementary to Fig. 4a). Mean ±s.d. are represented. c) Percentage of NK cells with TIGIT and LAG3 expression in BM in YFP_cγ1_ control mice (n = 4) and in mice with MGUS (n = 6) and MM (n = 26) (complementary to Fig. 4a). d) Tumors with higher number of immune cells (n = 27-34) in the BM contained an increased number of tumor-reactive CD8^+^ T cells that expressed TIGIT, and LAG3, in contrast to those cases with lower immune infiltrates (n = 22-25) (complementary to Fig. 4d). e) Immunohistochemical studies using antibodies to detect GFP^+^ transgenic MM cells or CD3^+^ T lymphocytes in BM sections from YFP_cγ1_ control mice, BI_cγ1_ mice and MI_cγ1_ mice. f) Representation of the percentage (%) of the area in a region of interest in the BM that is occupied by CD3^+^ T cells with respect to non-GFP^+^ MM cells. Samples from YFP_cγ1_ control mice (n = 2), BI_cγ1_ mice (n = 2) and MI_cγ1_ mice (n = 2) were included. g) Pearson correlation analyses between the percentages of NK cells in the mouse BM and those of T_reg_ cells in the BM (complementary to Fig. 4d). h) In MM patients, tumors with more abundant infiltrating immune cells (n = 31) contained an increased number of tumor-reactive PD-1^+^ CD4^+^ T cells and NK cells in the BM compared with MM cases with lower number of immune cells (n = 69) (complementary to Fig. 4g). i) Pearson correlation analyses between the percentages of NK cells in the BM of MM patients and those of T_reg_ cells in the BM (complementary to Fig. 4h). Boxes represent median, upper and lower quartiles and whiskers represent minimum to maximum range (d and h). Two-tailed Mann-Whitney test P values (d and h) are indicated. **p < 0.01; ***p < 0.001.

Immune surveillance restricts clinical progression in individuals with MGUS and SMM for extended periods^2,3,8^. To give further insights from the models, sequential changes in the BM immune microenvironment were determined by multi-parametric flow cytometry in mice with different genotypes at sequential disease stages. A linear increase in the number of T lymphocytes and natural killer (NK) cells was observed during progression, which correlated with PC expansion (Fig. 4a,b). CD8^+^ T cells acquired a CD44^+^CD62L^−^ effector phenotype and sequentially expressed the exhaustion markers PD-1, TIGIT and LAG3, while NK cells also exhibited activated phenotypes (Extended Data Fig. 4a–c). Due to the wide range of T cell and NK cell infiltration observed in the BM microenvironment across the different mouse strains, we divided MM cases according to the abundance of T and NK cells (Fig. 4c). A subset of MM cases (25 of 59, 42%) exhibited an immune cell infiltrate that resembled the BM microenvironment of healthy mice, while a subset of cases (34 of 59, 58%) was characterized by more abundant lymphoid cells, primarily CD8^+^ T lymphocytes with exhausted phenotypes (Fig. 4d and Extended Data Fig. 4d). Immunohistochemical studies in the BM revealed that T lymphocytes localized preferentially at the MM focal areas (Extended Data Fig. 4e,f). In addition, cases with more abundant T lymphocytes and NK lymphocytes contained a higher number of immunosuppressive CD4^+^CD25^+^Foxp3^+^ T_reg_ cells. The burden of CD8^+^ T lymphocytes, but not of NK cells, correlated with the number of T_reg_ cells, suggesting that T cell cytotoxic and immunosuppressive states interact during MM development in mice (Fig. 4d and Extended Data Fig. 4g).

**Extended Data Fig. 5:**
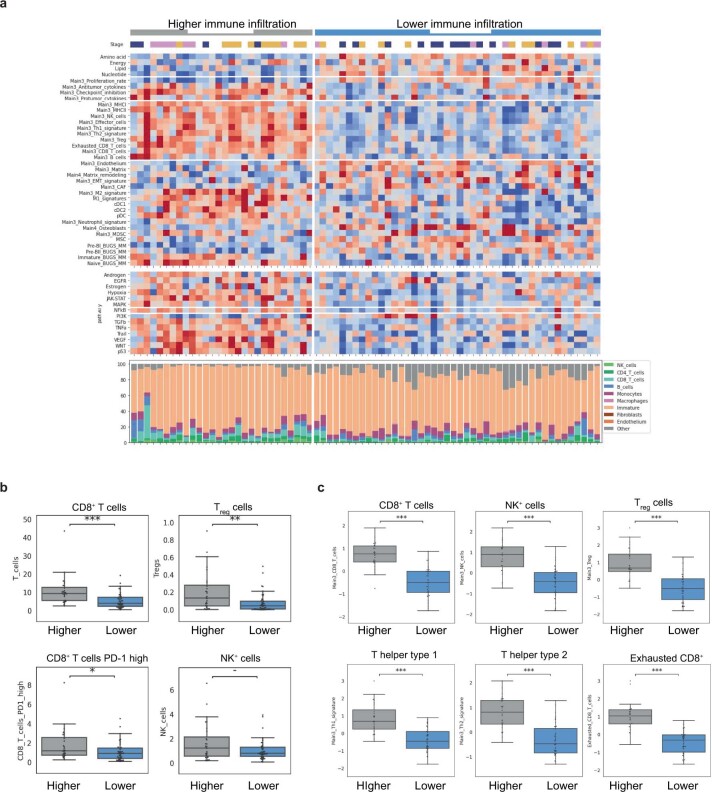
Bio-informatic deconvolution of RNAseq data. Bio-informatic deconvolution of RNA-seq data was applied to a previously reported clinical series of 72 newly diagnosed MM patients (GSE104171), which allowed the definition of the cellular composition of the BM microenvironment^34,35^. a) These studies confirmed the presence of the MM immunological subgroups, which divided the patients into two immune categories according to the abundance of immune cells in the BM. b) In this clinical series, patients with abundant immune cells MM (28 cases, 39%) presented higher number of PD-1^+^CD8^+^ T cells and T_reg_ cells with respect to those patients with low-infiltrating MM cases (44 cases, 61%). c) In addition, the transcriptomic signatures corresponding to CD8^+^ T cells, T_reg_ cells, NK cells and T_helper_ type 1 and type 2 cells were increased in the cases with higher number of immune cells with respect to those with less abundant T and NK cells.

To explore similarities with human disease, we examined the BM immune microenvironment in primary samples from individuals newly diagnosed with MGUS (*n* = 108), SMM (*n* = 167) or MM (*n* = 652) by multi-parametric flow cytometry. A progressive increase in T cell and NK cell populations was observed during the sequential MM stages, which correlated with MM cell burden (Fig. 4e,f). According to the classification described above, the cohort of MM patients was divided into those with lower and higher numbers of infiltrating T cells and NK cells (Fig. 4g). Of 652 MM cases, 435 (67%) were characterized by T cell and NK cell infiltrates that matched those in healthy donors. In contrast, the remaining 217 cases (33%) corresponded to those with a higher number of CD4^+^ and CD8^+^ T lymphocytes and NK cells (Fig. 4g,h and Extended Data Fig. 4h). Mimicking results in mice, the number of T_reg_ cells was higher in the cases with a higher number of immune cells, and was correlated with the abundance of CD8^+^ T lymphocytes, but not with NK cells (Fig. 4h and Extended Data Fig. 4i). The presence of the MM subgroups with lower and higher immune infiltrates was validated in a previously reported clinical series of MM (Extended Data Fig. 5)^34,35^. In summary, remodeling of the BM microenvironment during progression classifies mouse and human MM into distinct immune subtypes according to the abundance of infiltrating T cells and NK cells.

**Extended Data Fig. 6:**
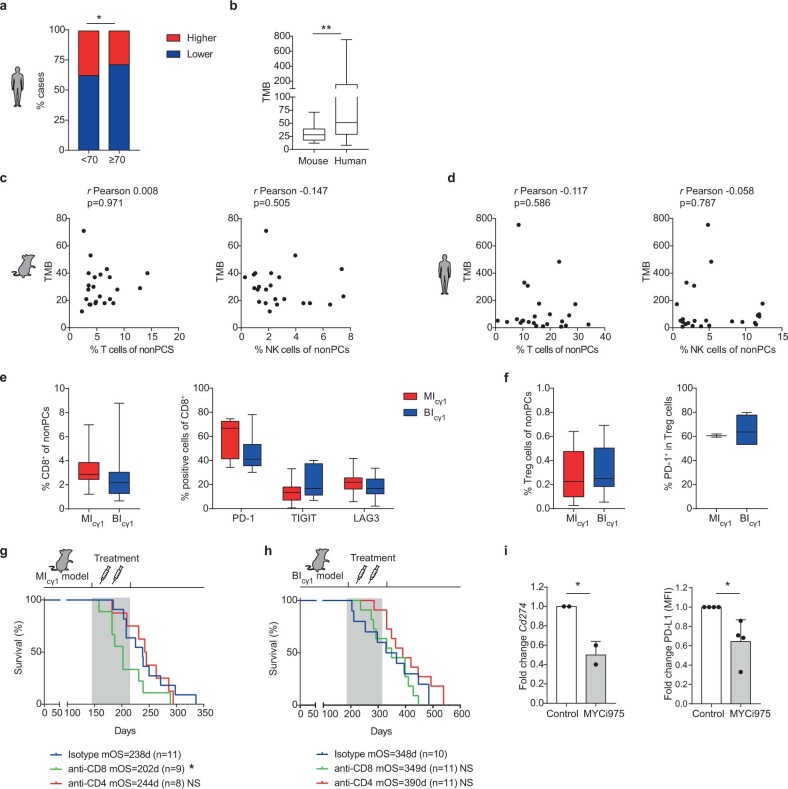
Immunological characterization of genetically heterogeneous mouse and human multiple myeloma. a) MM with lower frequency of immune cells was more common in patients older than 70 years. Fisher’s exact test. b) Measurement of the tumor mutation burden in MM samples from mice (n = 23) and patients (n = 24) according to whole-exome sequencing (WES) studies of somatic mutations. Pearson correlation studies of the TMB quantified and the T and NK cells infiltrating the BM in mouse MM (c) and human MM (d); MGUS and SMM cases were not included in these correlations. e) Comparison of the BM immune phenotypes including the number of activated PD-1^+^, TIGIT^+^, and LAG3^+^ CD8^+^ T lymphocytes at MM stages in MI_cγ1_ (n = 13) *vs*. BI_cγ1_ (n = 13) mice (complementary to Fig. 5c). f) Comparison of the number of PD-1^+^ Treg cells in the BM of MI_cγ1_ (n = 3) mice and in BI_cγ1_ (n = 5) mice (complementary to Fig. 5c). g-h) Kaplan-Meier survival curves in MI_cγ1_ mice and BI_cγ1_ mice undergoing depletion of CD4^+^ and CD8^+^ T cells. Monoclonal antibodies were administered by i.p. injection when MI_cγ1_ and BI_cγ1_ mice were 4.5 and 6 months of age, respectively. Mice received 100 µg of anti-CD4, anti-CD8, or rat IgG control antibodies, administered on days +1, +4, and +8 and then weekly for 8 weeks. Median overall survival, mOS. The number of mice included on each cohort is represented. i) Pharmacological inhibition of MYC repressed *Cd274*/PD-L1 expression at transcriptional and protein levels in the MM2732 cell line established from the Trp53-MI_cγ1_ model. Mean and s.d. of 2-4 independent experiments are shown. Boxes represent median, upper and lower quartiles and whiskers represent minimum to maximum range (b, e and f). Two-tailed t test or Mann-Whitney test P values (b, e, f and i) are indicated. Log-rank (Mantel-Cox) test was used in g and h. *p < 0.05; **p < 0.01; NS, non-significant.

Next, we investigated whether these categories were associated with MM biological and clinical characteristics in mice and patients. Cases with higher number of immune infiltrating cells exhibited higher levels of monoclonal immunoglobulin in serum, as a surrogate of the increased MM cell burden (Fig. 4i). In addition, the BM immune phenotypes correlated with age, with the quantity of the BM infiltrating T and NK lymphocytes negatively correlated with aging (Fig. 4j and Extended Data Fig. 6a). However, in mouse models and humans, the distribution of tumor-reactive lymphoid cell infiltrates was similar among the MM genetic subgroups, including the standard-risk and high-risk categories (Fig. 4k)^5^. Additionally, and contrary to other cancers^36^, quantification of the TMB from WES analyses in MM cells did not reveal a correlation with BM immune features (Extended Data Fig. 6b–d). In conclusion, MM immune categories correlate with the number of tumor cells and with aging, but not with the genetic-risk groups or the TMB.

### A CD8^+^ T cell versus T_reg_ cell ratio modulates immunotherapy responses

**Fig. 5:**
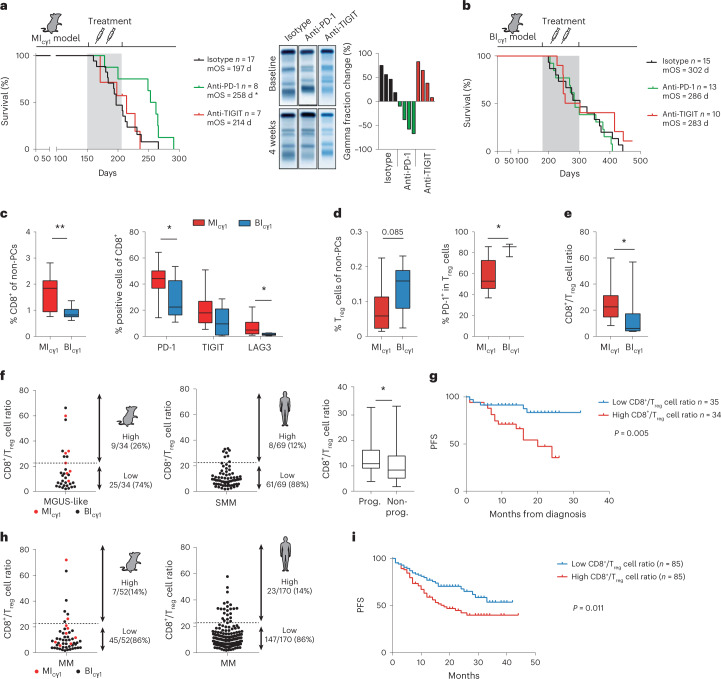
Immunotherapy responses in multiple myeloma. **a**, Preclinical immunotherapy trial in MI_cγ1_ mice testing anti-PD-1 or anti-TIGIT monoclonal antibodies with respect to isotype-treated mice. Kaplan–Meier OS curves and mOS values are shown. **b**, Preclinical immunotherapy trial in BI_cγ1_ mice testing anti-PD-1 or anti-TIGIT monoclonal antibodies with respect to isotype-treated mice. Kaplan–Meier OS curves and mOS values are shown. **c**, MI_cγ1_ mice (*n* = 10) exhibited higher numbers of activated PD-1^+^, TIGIT^+^ and LAG3^+^ CD8^+^ T lymphocytes in the BM compared with BI_cγ1_ mice (*n* = 9). **d**, The number of PD-1^+^ T_reg_ cells in the BM of MI_cγ1_ mice (*n* = 9) was lower than in BI_cγ1_ mice (*n* = 9) at MGUS stages. **e**, The ratio of CD8^+^ T cells to T_reg_ cells in the BM microenvironment was higher in MI_cγ1_ mice (*n* = 8) than in BI_cγ1_ mice (*n* = 9; median value, 22.5 versus 6.1; *P* = 0.019). **f**, Representation of the CD8^+^ T/T_reg_ cell ratio in BM samples from mouse MM and from patients with SMM. Median value of CD8^+^ T/T_reg_ cell ratios in SMM patients with progression versus those without progression at 2 years from diagnosis (*P* < 0.05; right). **g**, Kaplan–Meier PFS curve for patients with untreated SMM (*n* = 69). A high CD8^+^ T/T_reg_ cell ratio was associated with shorter time to progression with respect to the remaining cases (median PFS at 2 years, 38% versus 88%; *P* = 0.005). **h**, In 170 newly diagnosed individuals with clinically active MM, 23 (14%) exhibited a high CD8^+^ T/T_reg_ cell ratio, while the remaining patients (86%) showed lower CD8^+^ T/T_reg_ cell ratios. **i**, Kaplan–Meier PFS curve for 170 MM patients aged >70 years treated with lenalidomide and dexamethasone in the GEM-CLARIDEX clinical trial (NCT02575144). The presence of a high BM CD8^+^ T/T_reg_ cell ratio was associated with a higher rate of progression in comparison with those cases with low values (PFS, 18 months versus not reached; *P* = 0.011). Boxes represent the median, upper and lower quartiles and whiskers represent minimum to maximum range (**c**–**f**). Unpaired two-tailed Student’s *t*-test or Mann–Whitney test *P* values (**c**–**f**) are indicated. Log-rank (Mantel–Cox) test was used in **a**, **b**, **g** and **i**. *P < 0.05; **P < 0.01; ***P < 0.001.

We then asked whether early and late MM progression in the MI_cγ1_ and BI_cγ1_ models could influence responses to immunotherapy, and particularly to immune checkpoint blockade (ICB) therapy. To this end, preclinical in vivo immunotherapy trials using monoclonal antibodies to inhibit the two immune checkpoint receptors PD-1 and TIGIT were performed. In MI_cγ1_ mice, anti-PD-1 therapy started at the MGUS stage and continued during 8 weeks markedly reduced tumor burden and delayed MM development in treated versus untreated animals (mOS, 258 versus 197 d; *P* < 0.05; Fig. 5a). In contrast, anti-PD-1 therapy in the BI_cγ1_ strain started at the MGUS stage did not induce responses in the treated cohort compared with control mice (mOS, 286 d versus 302 d; *P* = 0.61; Fig. 5b). Similar therapy strategies with the anti-TIGIT monoclonal antibody did not yield therapeutic benefits in MI_cγ1_ or BI_cγ1_ mice (Fig. 5a,b and Supplementary Fig. 7). We investigated whether the composition of the BM microenvironment at precursor stages modulated responses to immunotherapy. MI_cγ1_ mice exhibited higher numbers of activated PD-1^+^, TIGIT^+^ and LAG3^+^ CD8^+^ T lymphocytes compared with BI_cγ1_ mice, but also showed a lower number of immunosuppressive CD4^+^PD-1^+^ T_reg_ cells (Fig. 5c,d and Extended Data Fig. 6e,f). Accordingly, the ratio of CD8^+^ T cells to T_reg_ cells was markedly higher in MI_cγ1_ than in BI_cγ1_ mice (median value of CD8^+^ T/T_reg_ cell ratio, 22.5 versus 6.1; *P* = 0.019; Fig. 5e). In this setting, in vivo depletion of CD8^+^ T lymphocytes, but not of CD4^+^ T cells, accelerated MM onset in MI_cγ1_ mice (Extended Data Fig. 6g). In contrast, depletion of CD8^+^ T cells did not modify survival of BI_cγ1_ mice, but rather, survival was extended upon CD4^+^ T cell depletion (Extended Data Fig. 6h). These findings show that the abundance of tumor-reactive CD8^+^ T cells versus the immunosuppressive T_reg_ cells characterized the rapid model of MM progression driven by MYC activation, which may have favored the activity of anti-PD-1 therapy. Because MYC can regulate the immune response by promoting *CD274* transcription in tumor cells^37,38^, we investigated this possibility in the mouse models. Pharmacological inhibition of MYC repressed programmed death-ligand 1 (PD-L1) expression at transcriptional and protein levels in MM cells from MI_cγ1_ mice (Extended Data Fig. 6i). These results suggest that early MYC activation triggered PD-L1 expression in MM cells to evade cytotoxic CD8^+^ T cell surveillance via PD-1 blockade, thereby explaining the selective efficacy of PD-1 inhibition in this model of early progression.

**Extended Data Fig. 7:**
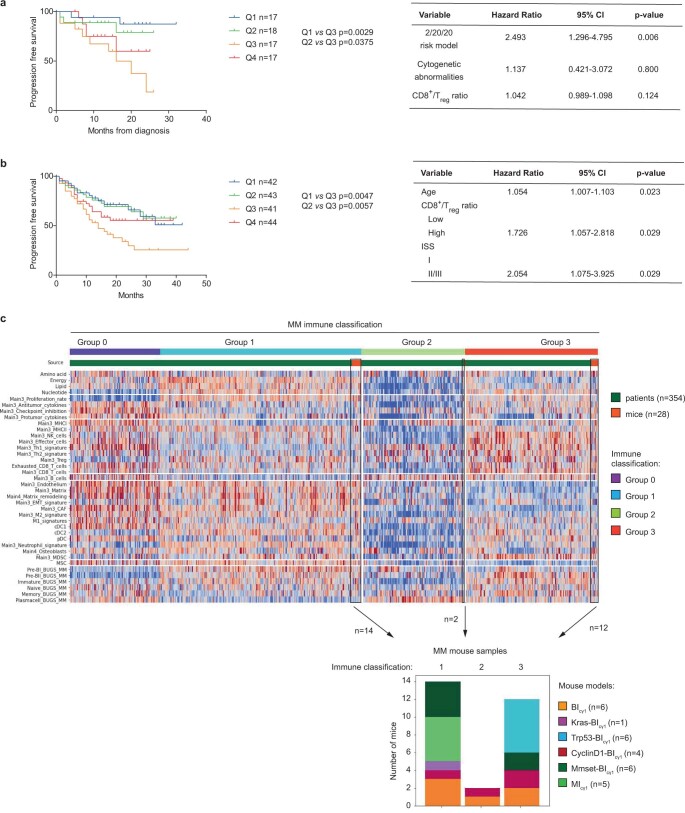
Immunological characteristics of mouse and human multiple myeloma. a) Kaplan-Meier progression-free survival (PFS) curves in the four quartiles, and Cox regression analysis of SMM patients (n = 69). b) Kaplan-Meier PFS curves in the four quartiles, and Cox regression analysis of newly diagnosed MM patients (n = 170). c) Bulk RNA-seq and microarray data analyses in mouse and human MM. The composition of the BM microenvironment was investigated in MM mouse samples with different genotypes (n = 28) and in data from the study of MM patient samples (n = 354) by applying bio-informatic reconstruction of the tissue microenvironment (TME) according to RNA-microarray and RNA-seq data from BM samples^34,36,40^. According to the composition of the BM immune microenvironment, patient samples were divided into four immune categories (group 0, group 1, group 2 and group 3). Integrative studies of the TME in mouse and human MM revealed that the MM in mice was classified into groups 1, 2 and 3, but not into group 0. Thus, the TME of in the mouse models of MM represents the TME of 307 of 354 human MM samples (87%). Log-rank (Mantel-Cox) test was used in a and b.

To explore the balance between cytotoxic and immunosuppressive T cells in patients, flow cytometry analysis was carried out in the BM of 69 patients with SMM who were followed up without receiving treatment. Those with a high CD8^+^ T/T_reg_ cell ratio exhibited a shorter time to progression into active MM with respect to the cases with low ratios (median progression-free survival (PFS) at 2 years, 38% versus 88%; *P* = 0.005; Fig. 5f,g and Extended Data Fig. 7a). These results indicate that a rapid progression in SMM occurs through the blockade of PD-1^+^CD8^+^ T lymphocytes by the tumor cells irrespectively of T_reg_ cells, and suggest that SMM patients at high risk of progression may benefit from anti-PD-1 therapy. Then, the ratio of BM CD8^+^ T cells versus T_reg_ cells was investigated in patients with newly diagnosed, clinically active MM. Among 170 patients, 23 (14%) exhibited a higher T cell ratio like in MI_cγ1_ mice, while the remaining individuals (147 cases, 86%) showed lower ratios comparable to those in BI_cγ1_-derived mice (Fig. 5h). The presence of a high CD8^+^ T/T_reg_ cell ratio predicting ICB responsiveness in only 14% of MM cases may provide a scientific rationale to the negative results of the anti-PD-1 monoclonal antibody in past clinical trials^15,16,39^. We then examined whether the BM T cell ratio could influence clinical responses to standard-of-care therapy. In MM patients aged >70 years treated with lenalidomide and dexamethasone in the GEM-CLARIDEX clinical trial (NCT02575144), those with a high BM CD8^+^ T/T_reg_ cell ratio showed a higher rate of early relapse in comparison with those with low values (PFS, 18 months versus not reached; *P* = 0.0114; Fig. 5i and Extended Data Fig. 7b). These findings reveal that the time to progression from precursor stages into MM shapes the BM immune microenvironment, which in turn influences clinical immunotherapy outcomes.

### Targeting the multiple myeloma immune microenvironment

**Fig. 6:**
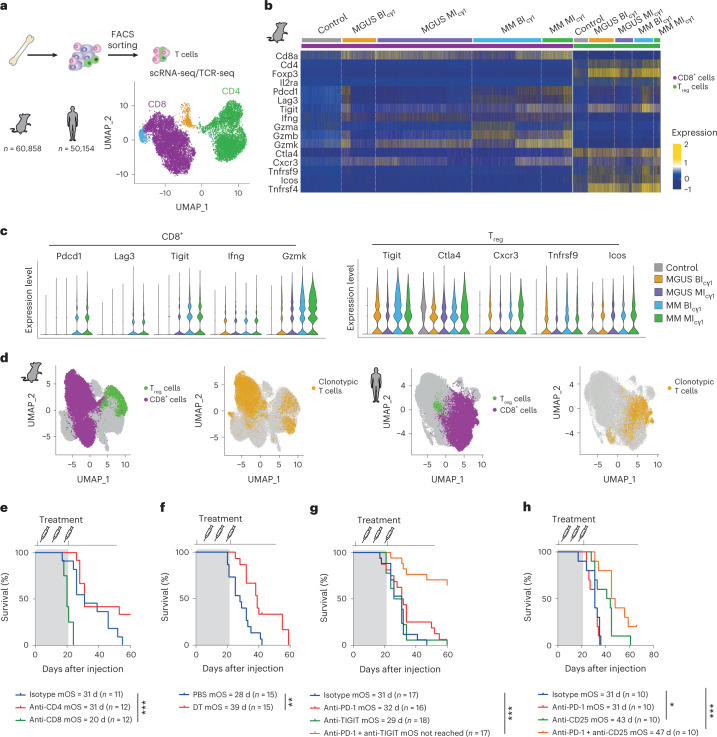
Modulating CD8^+^ T/T_reg_ cell ratio enhances immunotherapy outcomes. **a**, scRNA-seq/TCR-seq analyses of 60,858 CD3^+^ T cells isolated from the BM of MI_cγ1_ and BI_cγ1_ mice, and from YFP_cγ1_ controls. Three mice from each subgroup at MGUS and MM states were included. In patients, scRNA-seq/TCR-seq analyses of 50,154 CD3^+^ T cells isolated from the BM of newly diagnosed MM (*n* = 7) and MGUS (*n* = 4), and from the BM of healthy adults (*n* = 6), were performed. **b**, Differential expression of genes in CD8^+^ T cells and CD4^+^CD25^+^Foxp3^+^ T_reg_ cells are shown across MM progression. **c**, Quantification of the expression of selected markers in CD8^+^ T cells in MI_cγ1_ and BI_cγ1_ mice at different disease states (left). Quantification of the expression of markers in CD4^+^CD25^+^Foxp3^+^ T cells in MI_cγ1_ and BI_cγ1_ mice and in MM patients at MGUS and MM stages (right). **d**, Uniform manifold approximation and projection (UMAP) plots of single-cell transcriptomic and TCR genomic profiles from CD8^+^ T cells and T_reg_ cells in mice and patients at MM states are shown. In mice and humans, cells with a clonotypic TCR were identified preferentially among the CD8^+^ T cell subset. **e**, In vivo depletion of CD4^+^ or CD8^+^ T cells in the MM5080 syngeneic transplantation model is shown. The Kaplan–Meier OS curve included two experiments. The mOS and the number of mice in each treatment cohort are shown. **f**, In vivo genetic depletion of T_reg_ cells in Foxp3-GFP-DTR mice with transplanted MM5080 cells. The mOS and the number of mice in each treatment cohort are shown. **g**, Enhancing CD8^+^ T cell cytotoxicity by TIGIT co-inhibition dictates anti-PD-1 responses. The mOS and the number of mice in each treatment cohort are shown. **h**, Depletion of T_reg_ cells with a mouse anti-CD25 monoclonal antibody delayed MM onset and increased anti-PD-1 responses. The mOS and the number of mice in each treatment cohort are shown. Log-rank (Mantel–Cox) test was used. *P < 0.05; **P < 0.01; ***P < 0.001.

**Extended Data Fig. 8:**
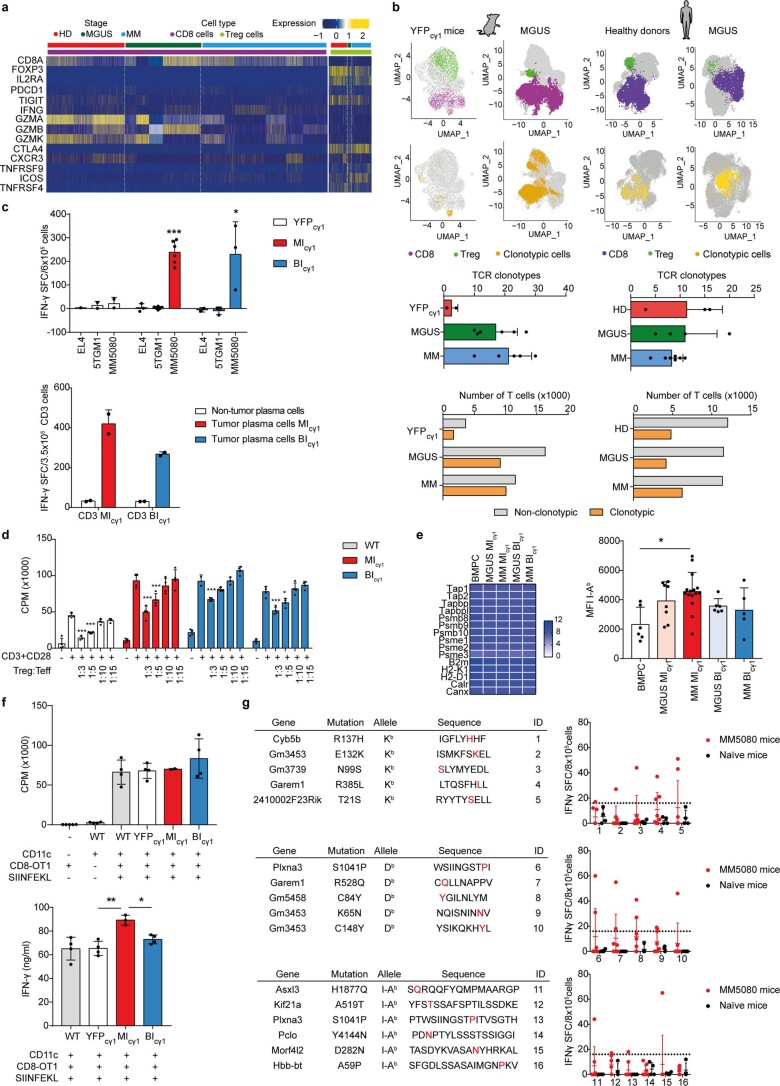
Functional evaluation of immunological features in mouse models of multiple myeloma. a) Differential expression of genes in BM CD8^+^ T cells and CD4^+^CD25^+^Foxp3^+^ Treg cells in MGUS and MM patients and healthy donor BM samples. b) UMAP plots of scRNA/TCR-seq data showing the cells with a clonotypic TCR among CD8^+^ T cells and CD4^+^CD25^+^Foxp3^+^ Treg cells in mice (n = 6) and patients (n = 4) at MGUS states, and in the BM of YFP_cγ1_ mice (n = 2) and healthy donors (n = 6) (complementary to Fig. 6d). At the bottom, the number of TCR clonotypes and the distribution of non-clonotypic and clonotypic T cells according to the TCR examination in mouse and human samples are shown. c) Specific recognition of MM cells by BM T lymphocytes. T cells from MI_cγ1_ (n = 6), BI_cγ1_ (n = 3) and YFP_cγ1_ (n = 2) mice were co-cultured with EL4, 5TGM1 and MM5080 cell lines (top). Additionally, GFP + B220^−^CD138+ primary MM cells obtained from the BM of MI_cγ1_ (n = 1) and BI_cγ1_ (n = 1) mice were co-cultured with the corresponding T cells. d) Co-culture assays with CD8^+^ T cells and CD4^+^CD25^+^ Treg cells from MI_cγ1_ (n = 1) and BI_cγ1_ (n = 2) mice. CD8^+^ T cell proliferation was measured with increasing concentrations of Treg cells. CPM, counts per minute per well. e) RNAseq analysis of the expression of MHC-I-related genes (left) and measurement of MHC-I/I-A^b^ surface expression by flow cytometry (right) in MGUS or MM cells from MI_cγ1_ and BI_cγ1_ mice with respect to BMPCs from YFP_cγ1_ mice (left). f) CD11c^+^ dendritic cells (DC) isolated from the spleen of MI_cγ1_ (n = 1), BI_cγ1_ (n = 1), and YFP_cγ1_ (n = 1) control mice showed similar MHC-I antigen presenting ability. CPM, counts per minute per well. g) List of 16 peptides containing potential neoantigens (neoAgs) in MM5080 cells, predicted to be highly immunogenic based on the affinity to bind to MHC-I and/or MHC-II molecules (left). On the right, functional validation assays of the peptides by co-culturing 8×10^5 ^splenocytes from MM5080 transplanted mice (n = 8) vs. non-transplanted animals (n = 4) with the corresponding neoAg peptide, presented by MHC class I Kb and Db molecules or by MHC class II I-Ab molecules, during 24 h. Data is represented as mean ± s.d. P values are obtained using one-way ANOVA test followed by Tukey’s multiple comparison test (c, d and f), Kruskal-Wallis adjusted for multiple comparisons by Dunn’s test (c and e) and Mann-Whitney test (c). *p < 0.05; **p < 0.01; ***p < 0.001.

To directly compare the BM immune portraits in mouse and human MM, we performed bioinformatic deconvolution of bulk RNA-seq data to reconstruct the tumor microenvironment (TME) in samples from newly diagnosed MM patients and from mice of different genotypes developing MM^34,40^. Integrative studies classified the TME of MM patients and mice into distinct overlapping immune subgroups, allowing all the 28 mouse samples to be matched to 307 (87%) of the 354 human MM samples (Extended Data Fig. 7c). To explore the functional interaction between T cell subsets in the TME, single-cell RNA-seq coupled with T cell antigen receptor (TCR) sequencing (scRNA-seq/TCR-seq) was conducted in BM CD3^+^ T lymphocytes from mice (*n* = 60,858 cells) and patients (*n* = 50,154 cells) at the MGUS and MM stages, along with BM T cells from mouse and human healthy controls (Fig. 6a). In MI_cγ1_ and BI_cγ1_ mice, markers of exhaustion/activation (*Pdcd1*, *Tigit*, *Lag3*) and cytotoxicity (*Ifng*, *Gzma*, *Gzmb*, *Gzmk*) were similarly expressed by CD8^+^ T cells at MM states, but these were barely detected in MGUS samples. T_reg_ cells from both mouse models also expressed markers of an activated/immunosuppressive state, including *Tigit*, *Ctl4*, Cxcr3, *Tnfrsf9* (encoding Cd137), *Icos* and *Tnfrsf4* (encoding OX40). Intriguingly, such a T_reg_ cell-activated phenotype was already evident in MGUS samples and maintained in the MM stage in both MI_cγ1_ and BI_cγ1_ mice (Fig. 6b,c). In patients, such early activation of T_reg_ cells was also evidenced at MGUS and MM states, in contrast to the phenotype of CD8^+^ T lymphocytes, which was minimally activated/exhausted at the MGUS state and became fully exhausted at the MM state (Extended Data Fig. 8a). In this setting, frequent clonotypic TCR sequences were found among CD8^+^ T cells in mice and patients, which were already present at the MGUS stage, suggesting a tumor antigen-driven function. In contrast, the number of clonal TCR sequences was markedly lower in T_reg_ cells (Fig. 6d and Extended Data Fig. 8b). Functional ex vivo assays in mouse cells demonstrated the immunosuppressive capacity of T_reg_ cells over CD8^+^ T lymphocytes, while the latter exhibited MM cell-specific immune recognition (Extended Data Fig. 8c–f). Further, by applying major histocompatibility complex (MHC)-binding predictive algorithms to nonsynonymous single-nucleotide variations (SNVs) identified by exome sequencing data from two mouse MM cell lines, we identified potential neoantigens with high binding capacity to MHC class I and/or class II molecules, a fraction of which were functionally validated as having specific T cell immunogenicity (Extended Data Fig. 8g and Supplementary Table 9). These results reveal similarities between mouse and human BM immune microenvironments at the single-cell level, and define functional characteristics in tumor-reactive cytotoxic and immunosuppressive T cell subsets during MM development.

**Extended Data Fig. 9:**
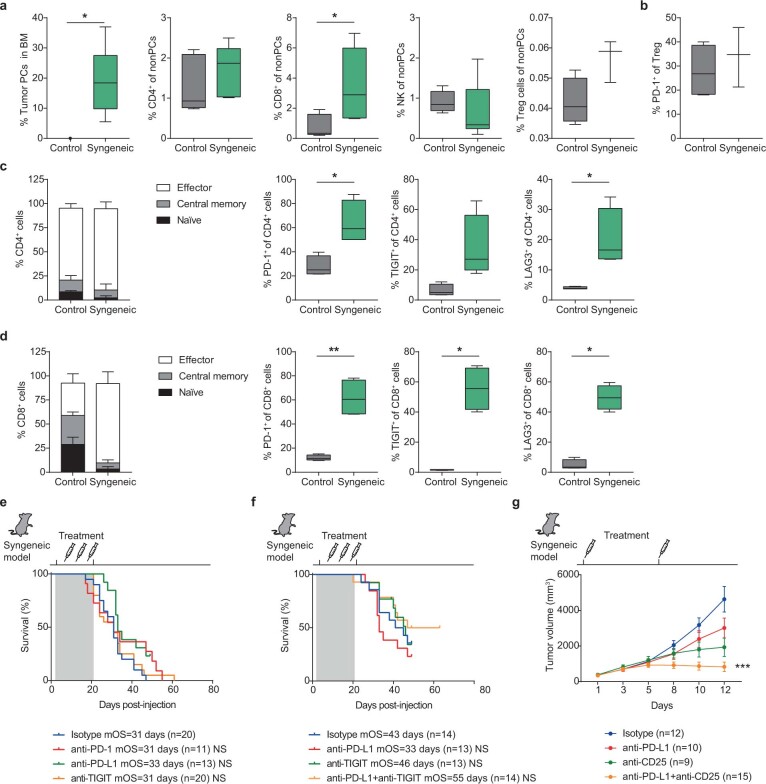
Syngeneic mouse models of multiple myeloma. a) Quantification of MM cells, CD4^+^ and CD8^+^ T lymphocytes, NK cells and T_reg_ cells in syngeneic MM5080 (n = 3-7) and control C57BL/6 (n = 3-6) mice is shown. b) Syngeneic transplants showed higher number of immunosuppressive PD-1^+^ Treg cells with respect to control C57BL/6 mice. c) Characterization of CD4^+^ T lymphocytes in syngeneic transplants (n = 4) *vs*. control C57BL/6 (n = 4) mice. d) Characterization of CD8^+^ T lymphocytes in syngeneic transplants (n = 4) *vs*. control C57BL/6 (n = 4) mice. e) Syngeneic transplants from the MM5080 cell line were refractory to therapies with moAbs that inhibit PD-1, PD-L1 and TIGIT. Therapy responses were determined by comparing median overall (mOS) in Kaplan-Meier survival curves. The number of mice included on each cohort is indicated. f) Simultaneous inhibition of PD-L1 and TIGIT moderately increased survival in a fraction of treated mice. Therapy responses were estimated by Kaplan-Meier survival curves. The number of mice included on each cohort is indicated. g) Depletion of T_reg_ cells with the anti-CD25 moAb combined with inhibition of PD-L1 efficacy decreased MM growth in the subcutaneous MM8273 syngeneic model. Boxes represent median, upper and lower quartiles and whiskers represent minimum to maximum range (a, b, c and d). P values obtained from two-tailed t tests (a, b, c and d), Mann-Whitney tests (a, b, c and d) and Kruskal-Wallis adjusted for multiple comparisons by Dunn’s test (g) are indicated. Log-rank (Mantel-Cox) test was used in e and f. *p < 0.05; **p < 0.01; ***p < 0.001; NS, not significant.

We next explored whether disturbing the balance between T cell cytotoxicity and immunosuppression experimentally would affect the response to ICB. To this end, anti-PD-1-resistant syngeneic transplants were established by intravenous injection of BI_cγ1_-derived MM cell lines into immunocompetent mouse recipients. Mice from one of the syngeneic models accumulated MM cells in the BM, along with abundant PD-1^+^, TIGIT^+^ and LAG3^+^ CD8^+^ T cells and a high number of PD-1^+^ T_reg_ cells (Extended Data Fig. 9a–d). In this context, no response to monoclonal antibodies inhibiting PD-1, PD-L1 and TIGIT was observed, in accordance with the distribution of T cell subsets in the BM microenvironment (Extended Data Fig. 9e). Depletion of CD8^+^ T cells markedly accelerated MM onset, while genetic depletion of T_reg_ cells in vivo delayed MM development, suggesting a role of CD8^+^ T cells and T_reg_ cells in the control of MM cells (Fig. 6e,f). Accordingly, mitigating CD8^+^ T cell exhaustion via TIGIT inhibition led to responses to both PD-1 and PD-L1 blockade, achieving durable MM responses (Fig. 6g and Extended Data Fig. 9f). Moreover, depletion of T_reg_ cells with a CD25 monoclonal antibody extended survival of mice, and enhanced efficacy of anti-PD-1 and anti-PD-L1 treatments (Fig. 6h and Extended Data Fig. 9g)^41^. Collectively, these data reinforce the notion that the BM CD8^+^ T/T_reg_ cell ratio predicts ICB responsiveness, and provide a potential biomarker to optimize MM immunotherapy in the clinic.

## Discussion

In contrast to other B cell malignancies, modeling MM in mice was difficult over the years^18–21^. Here, we generated 15 mouse models that fulfill the primary clinical, genetic and immunological characteristics of MM. Our in vivo genetic screen showed that the major experimental constraint to MM modeling is the recapitulation of its cellular origin from B lymphocytes, which once mutated have to transit through the GCs in lymphoid organs, terminally differentiate into plasmablasts, and home to the BM as PCs before progressing to the full malignant phenotype. Thus, transgenic B cells that failed class-switch recombination in GCs, migration to the BM or terminal PC differentiation did not induce MM, but rather generated other B cell malignancies including diffuse large B cell lymphoma, a disease that shares a GC B cell origin and multiple genetic features with MM^42^. Our results conclude that specific genetic changes that occur in GC B cells drive MM when bypassing these obstacles, while GC-derived lymphomas are induced by other combinations of mutations that do not. In this scenario, we show that IgM-secreting MM constitutes a distinct BM disease of non-class-switched PCs derived from early B lymphocytes, which matches recent studies in patients with IgM MM^27^.

Mice with the different transgenic lesions develop MM by acquiring comparable genetic abnormalities during disease evolution, defining a common MAPK–MYC oncogenic axis that underlies progression from pre-malignant states. These findings match the recurrently mutated pathways observed in MM patients, which lead to the activation of shared oncogenic cascades^4,6^. Based on these experimental results, we propose that MM is driven by genetically heterogeneous lesions that converge in a common MYC oncogenic pathway, which imposes time to progression. In this line, MYC activity also governs the immune-escape mechanisms that reshape the BM microenvironment through MM development, and conditions immunotherapy outcomes. Our experimental and clinical data highlight the value of an elevated ratio of tumor-reactive CD8^+^ T cells to immunosuppressive T_reg_ cells in the BM as a predictor of immunotherapy responses, particularly to PD-1/PD-L1 inhibitors. In line with our observations, the frequency of PD-1^+^CD8^+^ T cells relative to that of PD-1^+^ T_reg_ cells in the TME predicted clinical efficacy of PD-1 blockade therapy in patients with advanced melanoma and gastric carcinoma^43^. Moreover, the ratio of CD8^+^ T cells over MM cells dictated Cd137 monoclonal antibody efficacy in the transplantable Vk*MYC model of MM, which could be enhanced by depleting T_reg_ cells^44^. In this context, we found that only 14% of individuals with late-stage MM showed a proportion of cytotoxic and immunosuppressive T lymphocytes in the BM that was predictive of an ICB response, which may provide a scientific explanation to the negative results in anti-PD-1 clinical trials^15,16^.

An example of the translational applicability of our preclinical models is the prediction of clinical responses to drugs targeting the genetic drivers of MM progression, including several MAPK and MYC inhibitors currently being tested in cancer patients^45,46^. Our findings indicate that one optimal scenario for testing these inhibitors could be SMM patients, as early treatment might prevent progression into currently incurable MM^13,47^. In contrast, our models anticipate that successful immunotherapy in MM patients will require a personalized approach based on the individual immunological profiles. Our data can explain why only a small subset of individuals with active MM responded to ICB therapy, and predict that the majority of MM patients will benefit from treatment strategies for the adequate disentanglement of cytotoxic and immunosuppressive T cell properties within the BM microenvironment. We show that a subset of these individuals is characterized by a prominent T_reg_ cell-driven immunosuppression, which reinforces the key role of T_reg_ cells in MM pathogenesis from early states^48^, and suggests that early T_reg_ cell depletion with CD25 monoclonal antibodies will be of clinical value^41^. Another subset of MM cases harbors a lower number of infiltrating immune cells in the BM, which could benefit from the use of co-stimulatory molecules such as Cd137 monoclonal antibodies, or from bi-specific T cell engagers such as BCMAxCD3 monoclonal antibodies^44,49,50^.

The mouse resources presented here are now available to the scientific community to advance MM preclinical research. However, we would like to highlight certain limitations of the models. First, a T cell-driven immunization with SRBCs was performed to increase transgenic PC formation via the cγ1-cre allele in splenic GCs in young mice kept in a specific-pathogen free facility^26^; transgenic plasmablasts then migrate to the BM and progressively induce clonal MM. However, a splenic GC hyperplasia was observed following immunization, which may have influenced tumor immunity during progression. Reducing such systemic immune activation can be achieved by limiting transgenic boost in the spleens of mice with tamoxifen-inducible aid-cre-ERT2 or cγ1-cre-ERT2 alleles^51,52^; however, preliminary studies with the aid-cre-ERT2 model suggest that, while the splenic hyperplasia can be reduced, MM will be developed at late age and with a variable penetrance. Further investigations are warranted to define the optimal cre-recombinase system and the immunization protocol that drive more suitable models of MM at a reasonable timing and with full penetrance. Second, mouse cells have an inherent resistance to immunomodulatory drugs (IMiDs), as these cannot bind properly to the mouse Crbn protein, contrarily to human cells^53^. Indeed, we tested lenalidomide or pomalidomide alone and combined with bortezomib and dexamethasone in the mouse models, confirming IMiD refractoriness in vivo. To solve this limitation, our mice were crossed with a strain carrying a humanized *CRBN* gene^54^, which yielded sensitivity to IMiDs in vivo. Third, we found that the degree of lymphoid infiltration in the BM and the frequency of the immune subtypes do not exactly match the underlying genotype in mouse and in humans; such discrepancy remains to be investigated. Additional modifications are required to circumvent the current weaknesses of the models, with the aim of making them closer to human MM.

In summary, we present a set of genetically heterogeneous mouse models that recapitulate the principal MM genetic and immunological characteristics, which serve to investigate biological aspects of the disease during progression, as can be used as platforms to test and predict response to immunotherapy drug combinations. We expect that preclinical studies in these mice will accelerate the cures for MM within this decade.

## Methods

### Mouse strains

Eight transgenic mouse strains carrying common MM genetic changes were used. Five were obtained from The Jackson Laboratory: B6(Cg)-*Gt(ROSA)26Sor*^*tm4(Ikbkb)Rsky*^/J mice with constitutively active NF-κB signaling by *IKBKB* expression and a GFP reporter^55^; 129S/Sv-*Kras*^*tm4Tyj*^/J mice with the KRAS^G12D^ mutation^56^; B6.Cg-Tg(BCL2)22Wehi/J mice with *BCL2* expression^57^; C57BL/6N-Gt(ROSA)26Sor^tm13(CAG-MYC,-CD2*)Rsky^/J mice with *c-MYC* expression and a truncated human CD2 reporter^58^; and B6.129P2-*Trp53*^*tm1Brn*^/J mice with *Trp53* deletion^59^. The two previously reported mouse strains Cg-Tg (Eµ-cyclin D1) and B6.Cg-Tg (Eµ-c-MAF), which represent t(11;14) and t(14;16), respectively, were also used^60,61^. Finally, Rosa26-hMMSET-II^Stop-floxed^ mice were generated as a model of t(4;14). To establish this model, a construct encoding human MMSET-II cDNA preceded by a loxP-flanked STOP cassette was integrated into the mouse Rosa26 locus (using Addgene plasmid 15912). Consequently, transgene transcription is controlled by a CAG promoter, and its expression can be detected by GFP expression, which is placed under control of an internal ribosomal entry site downstream of the cDNAs. The linearized targeting vector was transfected into mouse embryonic stem cells, and targeted clones were isolated using positive (NeoR) selection. Correct integration was verified by Southern blot of EcoRI-digested genomic DNA from mouse embryonic stem cells and founder mouse tails using a Rosa26-specific probe (external Rosa probe A) and by PCR^62^. Transgenic activation was obtained by crossing mice with two cre-recombinase mouse lines: mb1-cre mice, kindly provided by M. Reth (University of Freiburg)^63^, and cγ1-cre mice (B6.129P2(Cg)-*Ighg1*^*tm1(cre)Cgn*^/J) obtained from The Jackson Laboratory^26^. As controls, mb1-cre or cγ1-cre mice crossed to B6.129 × 1-Gt(ROSA)*26Sor*^*tm1(EYFP)Cos*^*/*J mice (The Jackson Laboratory), which carry a YFP reporter, were generated^64^. The Vk*MYC mice, which die of human-like MM at late age, were also included as a positive disease control^17^. Strains were intercrossed by conventional breeding to obtain the corresponding compound mice with heterozygous or homozygous alleles, which were maintained in a hybrid C57BL6/129Sv genetic background. Mice of both sexes were used in the study. Mice were kept under specific-pathogen-free conditions in the animal facilities of the Center for Applied Medical Research (CIMA) at the University of Navarra. Animal experimentation was approved by the Ethical Committee of Animal Experimentation of the University of Navarra and by the Health Department of the Navarra Government. Genotyping protocols were performed using primers described in Supplementary Table 10.

### Genetic screens and immunization protocol

To model MM genetic heterogeneity, the eight strains of transgenic mice carrying MM genetic drivers were bred to engineer strains with single, double or triple genetic alterations (Supplementary Table 1). Genetic abnormalities were triggered in immature pre-B lymphocytes or mature GC B lymphocytes using mb1-cre or cγ1-cre mice, respectively^26,63^. To induce the formation of GFP^+^ transgenic PCs in mice housed under specific-pathogen-free conditions, animals were subjected to T cell-mediated immunization with SRBCs, which were prepared in a solution of 1 × 10^10^ cells per ml of 100% stock solution (Fitzerald) diluted in DPBS. Mice were intraperitoneally (i.p.) administered 100 µl of the SRBC solution at 8 weeks of age and were injected again every 21 d for 4 months. After immunization, a fraction of six-month-old mice from each cohort (*n* = 4–6) were necropsied and analyzed to determine the presence and characteristics of B cells and PCs in spleen and BM (Supplementary Fig. 1). The remaining mice from each cohort were monitored for tumor development up to 12 months of age (Supplementary Table 1). YFP_mb1_, YFP_cγ1_ and Vk*MYC mice were similarly immunized and characterized as controls. Survival rates of these diverse mouse strains were estimated using Kaplan–Meier OS curves.

### Flow cytometry analyses and cell sorting

Cell suspensions from spleen (obtained by mechanical disruption) and BM (flushed from femurs with DPBS) were filtered through a 70-µm cell strainer (Falcon) and treated with ACK lysis buffer to remove red blood cells. Then, cells were washed in DPBS and filtered a second time before they were labeled with antibodies for flow cytometry analysis. Mouse antibody panels (Supplementary Table 10) were used to detect tumor and immune cell subpopulations. Data acquisition was performed in a FACS CantoII flow cytometer (BD Biosciences) and analyzed using FlowJo v10.7.1 software. For cell sorting, stained cells were separated using a FACS Aria sorter instrument (BD Biosciences). Characterization of the BM microenvironment was performed by flow cytometry in 22 control, 44 MGUS and 59 MM mice representing the different genetic subgroups. Immune infiltration of the BM was evaluated according to the percentages of T cells (CD4^+^ plus CD8^+^) and NK cells present in the non-tumor fraction. Mice presenting an immune infiltration similar to that of control age-matched mice were considered as having a low number of immune cells in the BM (cutoff value, 1.8 times the mean value in the control group), while tumors with higher percentages of T and NK cells were classified as having a high number of immune-infiltrating cells.

### Serum protein electrophoresis and enzyme-linked immunosorbent assay

Sera were extracted from blood obtained by puncture of the submandibular vein and collected in a Microvette Z gel tube (Sarstedt). A 10-µl fraction was applied to an agarose gel (HYDRAGEL 30 Protein), which was analyzed in a semiautomated Hydrasys 2 device; this device quantified the serum protein components that were separated into five fractions by size and electrical charge. The gamma-globulin (γ) fraction in diseased mice was measured and compared with that in control aged-matched mice. In selected samples, an isotyping multiplex assay was used to simultaneously quantify immunoglobulin isotypes in serum using the MILLIPLEX Mouse Immunoglobulin Isotyping kit (Merck) on the Luminex xMAP platform.

### Laboratory analyses

Hemogram tests were performed with 10 µl of blood collected in a Microvette EDTA tube (Sarstedt) using an Element HT5 (CMV Diagnóstico Laboratorio) instrument. Calcium levels were detected by standard laboratory methods in a Cobas 8000 analyzer (Roche Diagnostics) at the Biochemistry Laboratory of the Clinic University of Navarra.

### Examination of bone lesions

Long mouse bones were examined using three-dimensional tomographic images acquired by X-ray micro-CT (Quantum-GX, Perkin Elmer). The three-dimensional tomographic images contained 512 slices with an isotropic 50-μm voxel size and a resolution of 512 × 512 pixels per slice. To perform the bone histomorphometry analysis, a region of interest containing the bone diaphysis and epiphysis (15 × 15 × 15 mm) was reconstructed from the original scan at a resolution of 30 μm per voxel using Quantum 3.0 software. Bone mineral density analysis in each region of interest was performed using a plugin developed for Fiji/ImageJ^65^. Studies were performed at the Imaging Platform at the Center for Applied Medical Research of the University of Navarra.

### *IghV* gene clonality

Two different strategies were used. First, *IghV* gene rearrangements were amplified by PCR in genomic DNA isolated from GFP^+^-sorted MM cells and splenic B220^+^ B cells from YFP_cγ__1_ mice using specific VHA, VHE and VHB forward primers and a reverse primer for JH4 (Supplementary Table 10). Individual fragments were purified from gel or directly from the PCR reaction mixture using NucleoSpin Gel and PCR Clean-up (Macherey-Nagel), sequenced, and blasted against the ImMunoGeneTics information system using a tool to determine VDJ usage (http://www.imgt.org/IMGT_vquest/). The second strategy consisted of the analysis of *Igh* gene clonality from the RNA-seq analysis in YFP^+^-sorted BM PCs from control YFP_cγ1_ mice and in GFP^+^ BM tumor cells from mice in the MGUS and MM states, through BCR reconstruction using the MiXCR tool^66^. Briefly, raw FASTQ data were analyzed by MiXCR v3.0.12 to reconstruct the BCR clonality based on the CDR3 clonotypes frequencies separately in *Igh*, *Igk* and *Igl* chains according to previously reported methods^34^. The presence of an explicit clonotype is determined by the first to second clonotype sizes (number of reads) ratio and by the fraction of the largest clonotype for each chain with sufficient coverage (*y* and *x* axes in the figure). Clonality is a measure of uneven quantity RNA reads for each uneven CDR3 sequence with normalization maximum of 100. The higher clonality corresponds to the sample with more explicit clonotypes.

### Immunohistochemistry

Spleen, bone and kidney tissues were fixed in 4% (wt/vol) formaldehyde (Panreac) for 72 h and washed in 70% ethanol before paraffin embedding. Tissue sections were stained with H&E and with specific monoclonal antibodies (Supplementary Table 9). An automated immunostaining platform (Discovery XT-ULTRA, Ventana-Roche) was used. Briefly, sections stained with rat anti-CD138 (clone 281-2; 1:20,000 dilution) were incubated with rabbit anti-rat secondary antibody (BA4001; 1:100 dilution). Then, the sections were incubated with goat anti-rabbit-labeled polymer using the EnVision^+^ System (Dako), and peroxidase activity was revealed using DAB^+^ (Dako). For stains with monoclonal anti-c-MYC (Y69; 1:100 dilution) or anti-GFP (D5.1; 1:100 dilution), slides were incubated with the visualization systems (OmniMap anti-Rabbit) conjugated to horseradish peroxidase. Immunohistochemistry reactions were developed using 30-diaminobenzidine tetrahydrochloride (ChromoMap DAB, Ventana, Roche) and purple chromogen (Discovery Purple Kit, Ventana, Roche). Finally, nuclei were counterstained in Hematoxylin II. In selected BM samples, Giemsa or alkaline phosphatase staining was performed according to standard procedures.

### Quantitative RT–PCR

A total of 1 μg of total RNA from MM GFP^+^-sorted cells was isolated with a NucleoSpin RNA kit (Macherey-Nagel) and reverse transcribed into cDNA using MMLV enzyme technology (Invitrogen). Real-time PCR was performed on an ABI Viia7 instrument using SYBR green fluorophore and primers designed to amplify specific mouse or human genes. Specific primers are listed in the Supplementary Table 10.

### Human multiple myeloma samples

Clinical BM aspirate samples from individuals of both sexes with newly diagnosed MGUS (*n* = 108), SMM (*n* = 167) or MM (*n* = 652) were analyzed by multi-parametric flow cytometry. In addition, 9 MGUS and 41 MM samples from newly diagnosed individuals were characterized by RNA-seq. BM aspirates from 24 adult donors of both sexes, ranging from younger to older ages (51 to 84 years; median age, 72.5 years), were included as controls. All samples were obtained from the University of Navarra Biobank. A series of 170 samples from patients of both sexes with newly diagnosed MM enrolled in the PETHEMA/GEM-CLARIDEX clinical trial (NCT02575144) were characterized by multi-parametric flow cytometry. A series of patients with 69 newly diagnosed SMM was included. This study was performed in accordance with the regulations of the Institutional Review Board of the University of Navarra and was conducted according to the principles of the Declaration of Helsinki. Informed consent was obtained from all patients.

### Flow cytometry analysis and cell sorting in human samples

Characterization of human samples was performed using the EuroFlow lyse-wash-and-stain using a standard sample preparation protocol adjusted to 10^6^ BM-derived nucleated cells, together with the eight-color combination of the monoclonal antibodies CD138-BV421, CD27-BV510, CD38-FITC, CD56-PE, CD45-PerCPCy5.5, CD19-PECy7, CD117-APC and CD81-APCH7 (BD Biosciences)^67^. Data acquisition was performed in a FACS CantoII flow cytometer (BD Biosciences). Samples were analyzed using the Infinicyt software (Cytognos SL) and the semiautomated pipeline ‘FlowCT’, based on the analysis of multiple files by automated cell clustering^68^. Cell sorting was performed in a FACS Aria sorter instrument. Classification of BM samples according to immune cell infiltration was calculated similar to that in the mouse samples. The maximum percentages of T cells and NK cells present in the BM from healthy control individuals (cutoff, 20%) were used to divide patients with MM into cases with low or high number of immune-infiltrating cells.

### Human multiple myeloma cell lines

Ten cell lines derived from individuals with MM (RPMI8226, KMS12, KMS26, KMS11, MM1S, U266, K620, JJN3, H929 and MOLP2) were included in this study. Cell lines were validated according to the AmpFLSTR Identifiler and were tested for *Mycoplasma sp*. (Supplementary Table 10).

### Generation of multiple myeloma-derived cell lines from primary mouse samples

Cell suspensions from BM and/or spleen samples from mice exhibiting MM development were injected through the tail vein of Rag2^−/−^ IL2γc^−/−^ immunodeficient mice (The Jackson Laboratory)^69^. Animals were monitored twice weekly for signs of disease and were then killed. Upon serial transplantations, cases that predominantly exhibited GFP^+^CD138^+^B220^−^IgM^−^ PCs cells were selected, and the cells were expanded in vitro. The samples that were able to grow for weeks ex vivo were tested for the presence of the original transgenic lesions and then characterized (Supplementary Fig. 4). Eight MM-derived cells lines established from mice carrying different lesions are listed in Supplementary Tables 5 and 10.

### In vitro therapy assays

For viability assays, mouse or human MM cells were seeded in 96-well black culture plates and treated with different drugs for 48 h. Cell viability was quantified using a Deep Blue Cell Viability Kit (BioLegend) and analyzed with a Skanit Varioskan Flash 2.4.3 (Thermo Scientific) fluorometer. Treatments were administered to cells at a density of 0.3 × 10^6^ cells per ml, and all tests were performed in triplicate. After treatment, cells were subjected to RT–qPCR or western blot analyses, as indicated, according to previously reported methods^70^.

### RNA sequencing

RNA-seq was performed in isolated BM GFP^+^CD138^+^B220^−^ PCs from mice at the MGUS (*n* = 25) and MM (*n* = 40) stages and in BM CD38^+^CD138^+^ PCs from patients with newly diagnosed MGUS (*n* = 9) and MM (*n* = 41). BM GFP^+^CD138^+^B220^−^IgM^−^ PCs (*n* = 6) and spleen B220^+^CD38^−^FAS^+^ GC B cells (*n* = 3) were isolated from T cell-immunized 6-month-old YFP_cγ1_ mice, and used as controls. In addition, human CD38^+^CD138^+^ PCs were isolated from BM aspirates from adult healthy donors (*n* = 7). RNA-seq was performed on 20,000 cells per sample using a reported MARS-seq protocol adapted for bulk RNA-seq with minor modifications^71^. Libraries were sequenced in an Illumina NextSeq 500 at a sequence depth of 10 million reads per sample. A second RNA-seq study was conducted in GFP^+^CD138^+^B220^−^ PCs isolated from 20 BM samples obtained from mice at the MGUS (*n* = 1) and MM (*n* = 19) stages. RNA was extracted from fresh-frozen samples maintained in TRIzol (Invitrogen), and libraries (PE 50 or 100 base pairs) were prepared using the TruSeq RNA sample kit and validated using an Agilent Technologies 2100 Bioanalyzer. Library preparation, sequencing and post-processing of the raw data were performed on an Illumina HiSeq 2500.

### Spectral karyotyping

Mouse MM cells were cultured, harvested and fixed according to standard cytogenetic protocols. Metaphase spreads from fixed cells were hybridized with the HiSKY probe (FPRPR0030). Slides were prepared for imaging using a CAD antibody kit (FPRPR0033, Applied Spectral Imaging) and counterstained with DAPI. Twenty metaphase spreads were then captured and analyzed using HiSKY software (Applied Spectral Imaging).

### Whole-exome sequencing

WES was performed in 71 BM samples isolated from GFP^+^CD138^+^B220^−^ PCs (purity, >99%), including 62 samples from the MM stage, 3 samples of pooled PCs from 9 mice at the MGUS stage (3 mice with similar genotype were included on each pooled sample) and 6 samples from MM-derived cell lines. As MM reference controls, 5TGM1 and 12598Vk*Myc cell lines were also characterized. Six BM samples with YFP^+^CD138^+^B220^−^ PCs (purity, >99%) isolated from YFP_cγ1_ mice were also included. Genomic DNA was purified using a NucleoSpin Tissue kit (Macherey-Nagel). DNA quality and concentration were evaluated using an Agilent 4200 Tape Station (Agilent) and a Qubit System (Invitrogen), respectively. Exome capture libraries were prepared according to the SureSelectXT mouse all exon target enrichment system (Agilent Technologies) and were sequenced using a 151 base-pair paired-end read protocol by Macrogen on an Illumina NovaSeq 6000. Sequencing resulted in a mean read depth of 112× (range 33–216×). The resulting FASTQ file analysis was performed with the Genome One platform (Dreamgenics). Raw FASTQ files were evaluated using the FASTQ and Trimmomatic quality controls. Each FASTQ was aligned with the GRCm38/mm10 version of the mouse genome reference with BWA-mem. Ordered BAM file generation was performed with SAMtools, and optic and PCR duplicate deletion was performed with Sambamba. SNVs and indels were identified with the combination of VarScan 2 and Dreamgenics to develop an algorithm for variant calling. Variants were annotated with Ensembl functional information, mouse population allelic frequencies from dbSNP, and an adaptation of the MGP database that did not include the wild-type mouse strain. Furthermore, a new database was generated with the variants identified in control mice. For potentially somatic preliminary variant selection in each sample, the following filters were applied: (a) Variants with total coverage of the affected position >20×, reads/variant ≥6 and allelic frequency >0.1; (b) absence of variants in dbSNP, MGP and the control database; (c) functional prediction of effects on protein; (d) absence of fault summary annotations; (e) frequency <0.05 of the variant in the sample; and (f) number of reads with the variant <5. Potential CNV identification analysis was performed with a MoCaSeq adaptation of CopywriteR.

### Whole-genome sequencing

WGS was performed in the two murine cell lines MM5080 and MM9275 and the corresponding matched germline DNAs. Briefly, genomic DNA was purified using a NucleoSpin Tissue kit (Macherey-Nagel). DNA quality and concentration were evaluated with a Qubit System (Invitrogen). Next-generation sequencing capture libraries were prepared according to the TruSeq Nano DNA Library (Illumina) and were sequenced using a 150 base-pair paired-end read protocol by Macrogen on an Illumina NovaSeq 6000. The resulting FASTQ file analysis was performed by the Genome One platform (Dreamgenics) using the HMMcopy adaptation of CopywriteR.

### Bulk RNA-seq and bioinformatic deconvolution

These studies were performed following reported methods^34,72^. Briefly, the sorted cell population compendium was used to develop a machine learning-based cell deconvolution algorithm to calculate the percentage of different cell types from bulk RNA-seq mixtures based on the minor difference between cell subpopulations. A two-stage hierarchical learning procedure for gradient boosting of a LightGBM model that included training on artificial RNA-seq mixtures of different cell types including immune and stromal cell populations was used. Artificial RNA-seq mixtures were created by admixing different datasets of sorted cells together in various cell proportions, and the LightGBM model was trained to predict the admixed cell percentage. Then, the model was used to reconstruct proportions of cell subpopulations using the information from the proportion of the major cell populations and subpopulations. The algorithm estimates the RNA proportion of a cell type in bulk RNA-seq mix of a sample, which could be converted into cell percentage if the RNA concentration of a cell type was known. Total RNA abundance in isolated cells was quantified using Qubic. Total BM samples from genetically diverse mice at MGUS (*n* = 6) and MM (*n* = 28) stages were included, along with three BM samples from healthy YFP_cγ1_ mice. For human sample analyses, public RNA-seq and microarray datasets corresponding to total or CD138-depleted BM samples from newly diagnosed MM patients (*n* = 426) in two clinical series were included: GSE136324 (ref. ^40^) and GSE104171 (ref. ^35^).

### Single-cell RNA-seq and TCR-seq

T cells were isolated by FACS based on expression levels of CD19, CD56, CD30e and CD3 for human cells and B220, CD3 and NK1.1 for mouse cells. scRNA-seq/TCR-seq was performed using 10X Genomics Single Cell 5′ Solution, version 2, according to the manufacturer’s instructions (10X Genomics). Libraries were sequenced on a NextSeq 500 (Illumina) and analyzed using Cell Ranger v3.0.0 software (10X Genomics). Quality-control metrics were used to select cells with mitochondrial genes representing <10% of total genes and with at least 200 genes. The final number of T cells characterized was as follows: 21,512 T cells from BI_cγ1_ (*n* = 3) and MI_cγ1_ (*n* = 3) mice with MGUS; 12,695 T cells from BI_cγ1_ (*n* = 3) and MI_cγ1_ (*n* = 2) mice with MM; 5,331 T cells from the BM of 6-month-old YFP_cγ1_ mice (*n* = 2); 32,988 T cells from newly diagnosed MM patients (*n* = 7); 15,870 T cells from patients with MGUS (*n* = 4); and 29,011 T cells from the BM of healthy adults (*n* = 6). Samples were analyzed using Seurat (https://satijalab.org/seurat/). Clonotypic TCRs were defined based on their presence in 10 or more cells. To integrate different scRNA-seq/TCR-seq samples we used a normalization and variance stabilization of molecular count data based on regularized negative binomial regression with a sctransfrom function^73^. Results were shown by UMAP plots of single-cell transcriptomic and TCR genomic profiles.

### Functional in vitro T cell assays

CD8^+^ T cells and CD4^+^CD25^+^ T_reg_ cells were isolated from the BM of control and MM mice. CD8^+^ T lymphocytes were stimulated with anti-CD3/anti-CD28 beads (bead:cell ratio of 1:3; Invitrogen) in the absence or presence of T_reg_ cells at decreasing ratios of CD8^+^ T/T_reg_ cells (1:3, 1:5, 1:10 and 1:15). Proliferation of CD8^+^ T cells was analyzed at day +4 by measuring tritiated thymidine incorporation using a scintillation counter. The number of tumor-specific interferon (IFN)-γ-producing cells was evaluated using the ELISPOT technique (BD Bioscience). Briefly, 6 × 10^5^ T cells were co-cultured with 6 × 10^4^ irradiated cells from EL4, 5TGM1 and MM5080 cell lines or with 10 × 10^4^ GFP^+^B220^−^CD138^+^ primary MM cells obtained from the BM of MI_cγ1_, BI_cγ1_ and YFP_cγ1_ control mice for 48 h in triplicate. Spots were measured using the ELISPOT reader (CTL). CD11c^+^ dendritic cells were purified by magnetic beads from the BM of MI_cγ1_, BI_cγ1_ and YFP_cγ1_ control mice and incubated with SIINFEKL peptide at 10 µg ml^−1^ during 2 h. After washing twice in PBS, SIINFEKL-specific dendritic cells were cultured during 48 h with CD8^+^ T cells of OTI mice (C57BL/6-Tg^TcraTcrb^1100Mjb/J), which carry a transgenic TCR designed to recognize ovalbumin peptide residues 257–264 (OVA257–264) in the context of H2Kb (CD8 co-receptor interaction with MHC class I). Culture supernatants were collected at 48 h and assessed for IFN-γ production by ELISA (Pharmigen).

### Prediction of potential neoantigens and functional validation

To define the landscape of neoantigens in two MM-derived cell lines (MM5080 and MM8273), we focused on nonsynonymous SNVs identified by exome sequencing data, and applied MHC-binding prediction algorithms to identify potential neoantigens containing these mutations. Briefly, 29-mer amino acid peptides containing the mutated residue at position 15 were designed. These sequences were applied to NetMHCPan 4.1 (https://services.healthtech.dtu.dk/service.php?NetMHCpan-4.1/) and NetMHCIIPan 4.0 (https://services.healthtech.dtu.dk/service.php?NetMHCIIpan-4.0/) to predict peptide binding to mouse H-2 Db and H-2 Kb class I and I-Ab class II molecules. For class I, 8-mer to 11-mer peptides containing the mutated residue and fulfilling the binding criteria (percentage rank of <2 for weak binders and <0.5 for strong binders) were selected. For class II, 15-mer peptides with a percentage rank of <10 for weak binders and <2 for strong binders were selected. A list of genes containing point mutations was defined for each of the cell lines. Given that some mutations may be contained by several overlapping peptides, a final list of potential neoantigen peptides (considering together MHC class I and class II epitopes) was determined (Supplementary Table 9). To explore whether the predicted neoantigens can be immunogenic to T lymphocytes, 16 peptides containing MM5080 somatic mutations, which were predicted to be highly immunogenic based on the affinity to bind to MHC class I and/or MHC class II molecules, were generated and subjected to functional assays. T cell responses present in tumor-bearing mice were measured by using an IFN-γ ELISPOT assay (BD Biosciences). Briefly, total BM cells (8 × 10^5^ per well) from eight syngeneic mice that developed BM tumors 14 d after injection of MM5080 cells were stimulated in antibody-coated plates for 24 h with neoantigen peptides (10 µM) or with irradiated (20,000 rads) tumor cells. As controls, BM samples from four non-transplanted animals were included. After washing and incubating with detection antibody for 2 h, spots were developed by using 3-amino-9-ethylcarbazole substrate. Spot-forming cells were counted with an ImmunoSpot automated counter (CTL-ImmunoSpot). Responses against peptides in tumor-bearing mice were considered positive if they were above two standard deviations of the mean of responses observed in naïve mice.

### Preclinical in vivo therapy trials

In vivo therapy trials were performed in MI_cγ1_ and BI_cγ1_ mice. Before therapy initiation, tumor burdens were estimated by measuring the immunoglobulin gamma fraction (M spikes) in serum by electrophoresis. Animals of both sexes with similar tumor burdens were separated into experimental groups. Depletion studies or immunotherapy preclinical trials were initiated when MI_cγ1_ and BI_cγ1_ mice were 4 and 6 months of age, respectively. Monoclonal antibodies were administered by i.p. injection once weekly for 8 weeks. Mice received 200 µg of anti-PD-1, anti-PD-L1, anti-TIGIT or rat IgG control antibody. For depletion studies, 100 µg of anti-CD4, anti-CD8 or rat IgG control antibody was administered on days +1, +4 and +8 and then weekly for 8 weeks. Therapy responses were determined by comparing serum M spikes at day 0 with those at 4 and 8 weeks after treatment initiation, and by mOS. All therapeutic regimens were well tolerated, with no evident body weight loss or overt signs of toxicity other than those attributable to the tumor itself. Animals were monitored twice weekly to detect any signs of discomfort and/or disease, which included hunching, ruffled fur, labored breathing, low body temperature, low mobility and/or >20% weight loss from the time of study initiation. Survival was estimated by Kaplan–Meier curves and was compared using the log-rank test.

### Multiple myeloma-derived syngeneic transplants and in vivo therapy

Establishment of syngeneic transplants was performed by injecting 5 × 10^6^ 5080MM cells in DPBS into the tail veins of 8- to 10-week-old C57BL/6 mice of both sexes. The MM8273 syngeneic model was established by subcutaneous injection of 10 × 10^6^ cells in DPBS in the flanks of 8- to 10-week-old C57BL/6 mice of both sexes. Upon injection of MM cells, animals of both sexes were randomly divided into experimental groups. CD4, CD8 or rat IgG control antibodies (100 µg each) were administered at days +1, +4, +8 and +16 after injection. To genetically deplete T_reg_ cells, B6.129 FoxP3 DTR mice (The Jackson Laboratory) were injected with 250 ng of diphtheria toxin weekly for 3 weeks starting on day +3 after injection. For immunotherapy studies, 200 µg of anti-PD-1, anti-TIGIT or anti-PD-L1 monoclonal antibodies was i.p. injected twice weekly for 3 weeks starting on day +3 after injection. Anti-CD25 (clone 7D4 (CD25 NIB), moIgG2a isotype) was administered by i.p. injection starting on day +3 after injection (75 µg per mouse) and continued weekly for three consecutive weeks. Therapy responses were estimated by Kaplan–Meier survival curves, which were compared using the log-rank test. In the subcutaneous MM8273 syngeneic models, therapy was started when tumors reached 400 mm^3^. Tumor growth was monitored every 2 d by measuring tumor size in two orthogonal dimensions using a caliper. Tumor volume was calculated using the formula *V* = (*L*^2 ^× *W*)/2.

### Statistical analysis

Statistical analyses were performed using GraphPad Prism 9.0 and SPSS v.25. Normality distribution was evaluated using the Shapiro–Wilk test. Next, parametric (two-tailed Student’s *t*-test or one-way analysis of variance test followed by Dunnett’s test for multiple comparisons) or non-parametric (Mann–Whitney *U* test or Kruskal–Wallis test followed by Dunn’s test for multiple comparisons) tests were used to evaluate the statistical significance. Mouse survival and human PFS were estimated using Kaplan–Meier curves and compared using the log-rank test. Multivariate analysis was performed using Cox proportional hazards analysis of PFS. Statistical values are indicated as *P < 0.05, **P < 0.01 and ***P < 0.001.

### Reporting summary

Further information on research design is available in the Nature Portfolio Reporting Summary linked to this article.

## Online content

Any methods, additional references, Nature Portfolio reporting summaries, source data, extended data, supplementary information, acknowledgements, peer review information; details of author contributions and competing interests; and statements of data and code availability are available at 10.1038/s41591-022-02178-3.

### Supplementary information

Supplementary Information Supplementary Figs. 1–7 with legends.

Reporting Summary

Supplementary Tables 1–10 Supplementary Table 1 Description of mouse mutant strains (*n* = 41), YFP_cγ1_/YFP_mb1_ control mice, and VK*MYC mice. Supplementary Table 2 GSEA from mouse RNA-seq data. a, Linear models for microarray/RNA-seq data analysis (LIMMA) was used to compare FACS-sorted MM cells from genetically heterogeneous mice (*n* = 38) and BM PCs from YFP_cγ1_ control mice (*n* = 6); b, LIMMA was used to compare MM cells from mice (*n* = 38) and spleen GC B cells from YFP_cγ1_ control mice (*n* = 3); c, LIMMA was used to compare MGUS cells from mice (*n* = 24) and BM PCs from YFP_cγ1_ control mice (*n* = 6). Supplementary Table 3 LIMMA was used to compare FACS-sorted MGUS (*n* = 9) and MM cells (*n* = 41) from patients and human BM PCs from healthy donors (*n* = 7). Supplementary Table 4 LIMMA was used in MGUS versus MM cells to compare the number of differentially expressed genes in MI_cγ1_ compared with BI_cγ1_-derived mice. Supplementary Table 5 List of MM cell lines established from primary samples in mice with MM. Supplementary Table 6 List of somatic mutations in MM (*n* = 62) and MGUS (*n* = 3) primary samples and in MM-derived cell lines (*n* = 6), also including the 5TGM1 and 12598 Vk*MYC mouse MM cell lines. a, MM samples; b, MGUS samples; c, MM cell lines. Supplementary Table 7 WES data in mouse models of MM. TMB, number of chromosomal changes and MAPK mutations at MGUS and MM states, distributed across strains. Supplementary Table 8 List of MAPK genes with somatic mutations found in mouse MM samples and in patients with MM in the CoMMpass study (*n* = 599). Supplementary Table 9 List of potential neoantigens identified in two MM-derived cell lines (MM5080 and MM8273) by analyses of nonsynonymous SNVs identified by exome sequencing data through MHC-binding prediction algorithms. a, MM5080 peptide MHC class I mutations; b, MM5080 peptide MHC class II mutations; c, MM8273 peptide MHC class I mutations; d, MM8273 peptide MHC class II mutations; e, Summary of data. Supplementary Table 10 Key resources table including all materials used in the manuscript.

### Source data

Source Data Fig. 1 and Extended Data Figs. 2 and 3 Unprocessed serum electrophoresis gels corresponding to gels in Fig. 1 and Extended Data Figs. 2 and 3.

Source Data Figs. 2 and 3 Unprocessed western blot images corresponding to blots in Figs. 2 and 3.

Source Data Fig. 4 Gating strategy for cytometry.

## Figures and Tables

**Extended Data Table 1:**
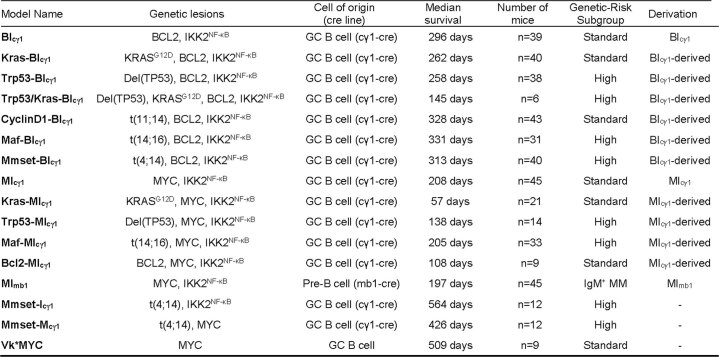
Genetically heterogeneous mouse models of MM

## Data Availability

Raw sequencing data was deposited on Gene Expression Omnibus with the following accession codes: GSE205447 (RNAseq data from mouse and human MGUS and MM samples and control mice and healthy donors); GSE205644 (bulk RNAseq data from mouse BM samples at MGUS and MM stages and control mice); GSE220997 (scRNAseq and TCR-RNAseq data from T-cells isolated from mice and patients and WES and WGS raw data form mouse samples and mouse cell lines).
