# Supplementary material for: Preclinical models for prediction of immunotherapy outcomes and immune evasion mechanisms in genetically heterogeneous multiple myeloma
Source: Nat Med. 2023 Mar 16;29(3):632–45. doi: 10.1038/s41591-022-02178-3 (PMC10033443; doi:10.1038/s41591-022-02178-3)
Supplement: Supplementary file 1 — Supplementary Figs. 1–7 with legends. [file 41591_2022_2178_MOESM1_ESM.pdf]

nature medicine

Article

https://doi.org/10.1038/s41591-022-02178-3

Preclinical models for prediction of immunotherapy outcomes and immune evasion mechanisms in genetically heterogeneous multiple myeloma

In the format provided by the authors and unedited

Supplementary Figure 1

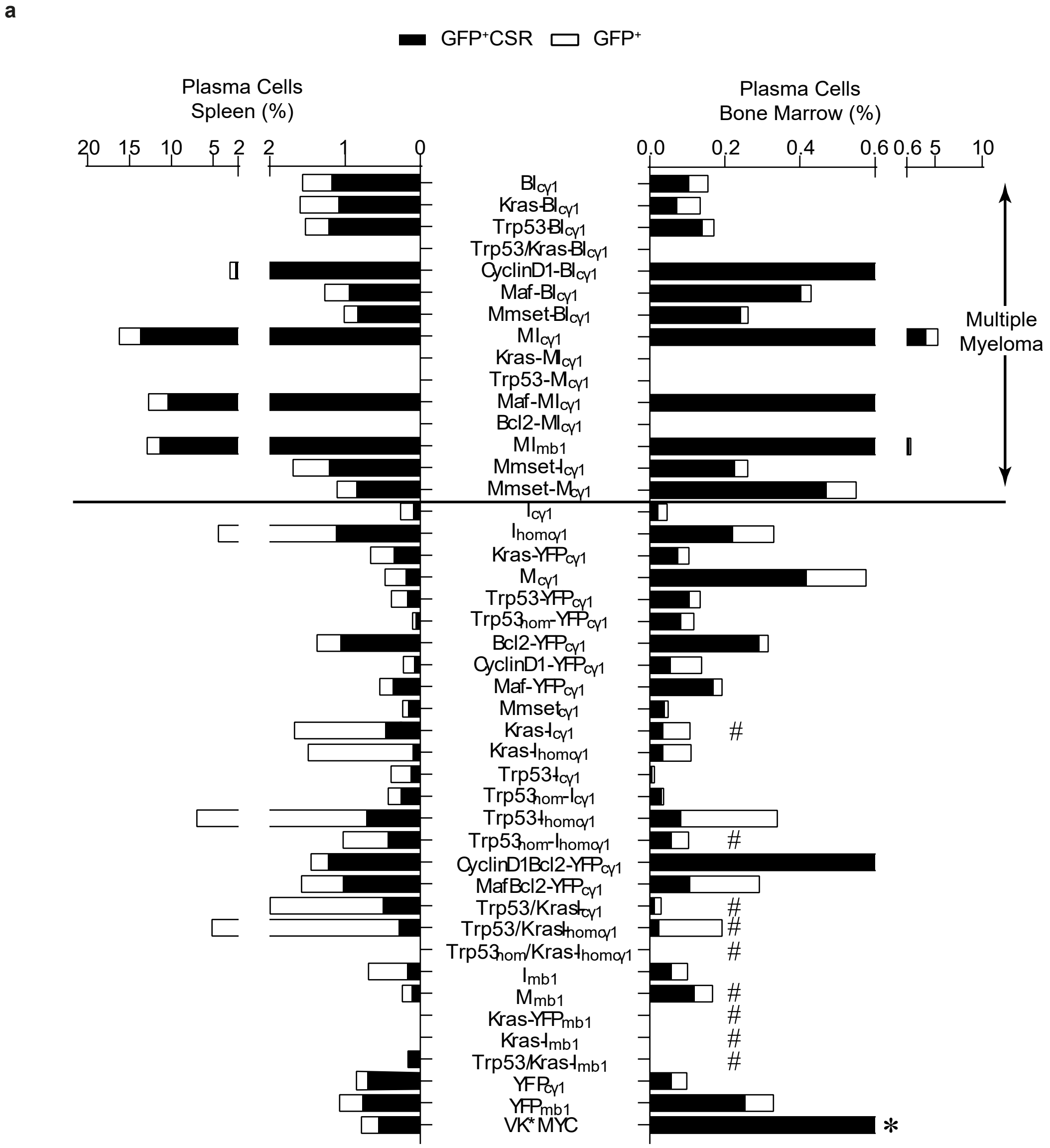

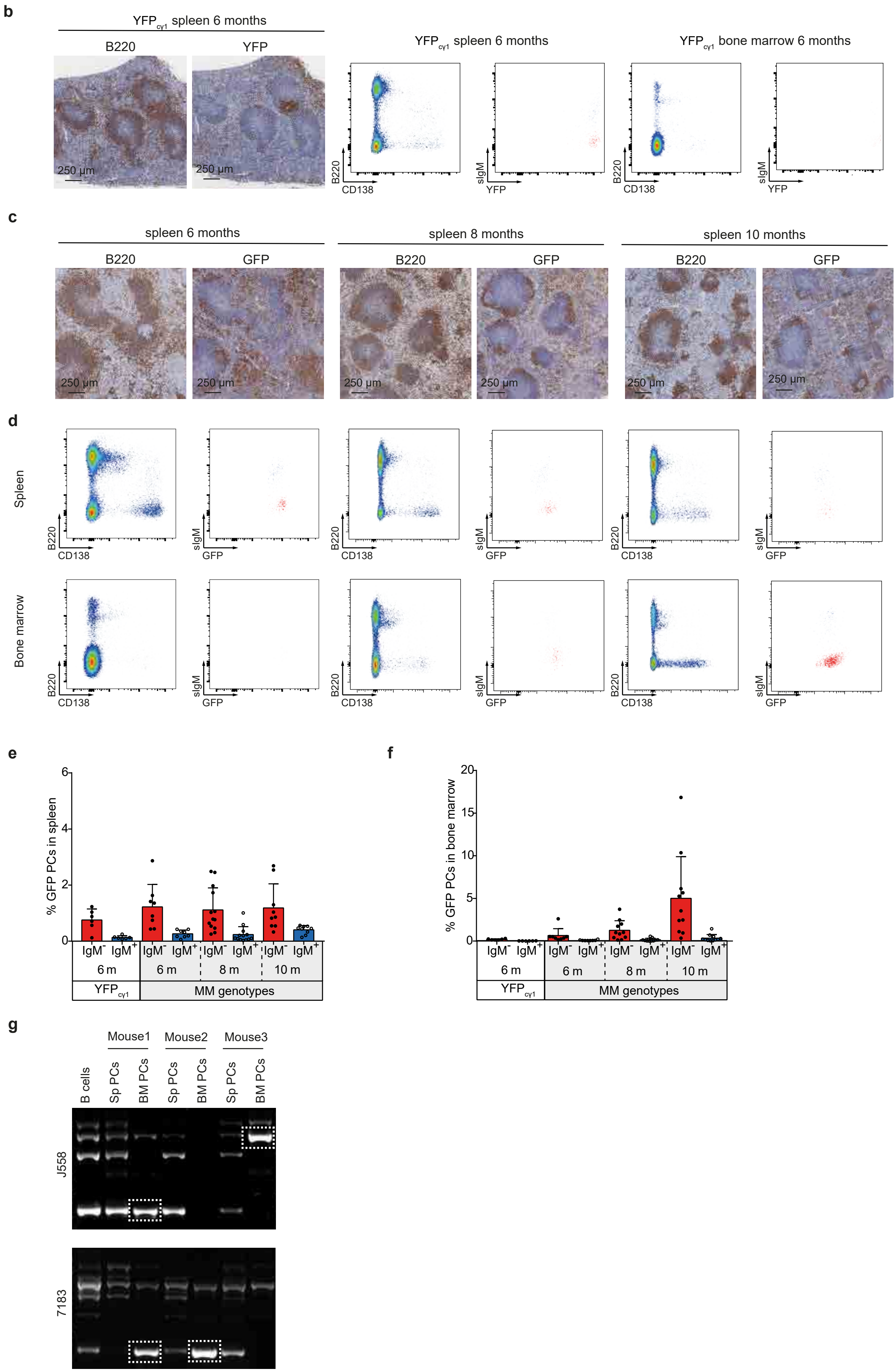
b
YFPcγ1 spleen 6 months
B220
YFP
250 µm
YFPcγ1 spleen 6 months
YFPcγ1 bone marrow 6 months
B220
CD138
sIgM
YFP
c
spleen 6 months
spleen 8 months
spleen 10 months
B220
GFP
250 µm
d
Spleen
Bone marrow
B220
CD138
sIgM
GFP
e
% GFP PCs in spleen
6
4
2
0
IgM- IgM+ IgM- IgM+ IgM- IgM+ IgM- IgM+
6 m
6 m
8 m
10 m
YFPcγ1
MM genotypes
f
% GFP PCs in bone marrow
20
15
10
5
0
IgM- IgM+ IgM- IgM+ IgM- IgM+ IgM- IgM+
6 m
6 m
8 m
10 m
YFPcγ1
MM genotypes
g
Mouse1
Mouse2
Mouse3
B cells
Sp PCs
BM PCs
Sp PCs
BM PCs
Sp PCs
BM PCs
J558
7183

Supplementary Figure 1. a) Flow cytometry analyses of BM and spleen samples from genetically heterogeneous mice, $YFP_{c\gamma1}$/YFP_{mb1} control mice, and VK*MYC mice at 6 months of age. CSR, class-switch recombination. Strains marked with # developed mature B-cell lymphoma or acute lymphoblastic leukemia, as shown in Supplementary Figure 2. (*) In Vk*MYC mice, the percentage of plasma cells in the BM and spleen correspond to total CSR cells. b) Representative example of immunohistochemical (IHC) examination of spleen from SRBC-immunized $YFP_{c\gamma1}$ control mice at 6 months of age, using B220 and YFP antibodies (left). On the right, flow cytometry study in a representative $YFP_{c\gamma1}$ control mouse at 6 months of age in spleen and in the BM, using B220, CD138 and sIgM antibodies. Transgenic, class-switched plasma cells ($YFP^{+}B220^{-}CD138^{+}sIgM^{-}$) are labeled in red. c) Representative examples of IHC studies in spleen from SRBC-immunized $BI_{c\gamma1}$ mice at 6, 8 and 10 months of age, using B220 and GFP antibodies. d) Flow cytometry studies in in spleen and in the BM from representative $BI_{c\gamma1}$ mice at 6, 8 and 10 months of age, using B220, CD138 and sIgM antibodies. Transgenic, class-switched plasma cells ($GFP^{+}B220^{-}CD138^{+}sIgM^{-}$) are labeled in red. e) Quantification of the % of $GFP^{+}CD138^{+}B220^{-}$ transgenic PCs in the spleen, according to the distribution of IgM^{+} and IgM^{-} subgroups, from SRBC-immunized $YFP_{c\gamma1}$ control mice (n=6) at 6 months of age and from SRBC-immunized $BI_{c\gamma1}$ mice at 6 (n=8), 8 (n=13) and 10 (n=9) months of age. f) Quantification of the % of $GFP^{+}CD138^{+}B220^{-}$ transgenic PCs in the BM of the same mice. g) Determination of *IghV* clonality by PCR on genomic DNA isolated from $GFP^{+}CD138^{+}B220^{-}$ transgenic PCs in spleen and in the BM at the time of death by MM in two $BI_{c\gamma1}$ and one $MI_{c\gamma1}$ mice. Specific VHA, VHE, and VHB forward primers and a reverse primer for JH4 are listed in the Supplementary Table 10. Clonal *IghV* bands in the BM of the three mice with MM are marked with squares, which were not detected in the spleen samples in the same mice, or in the control mice.

Supplementary Figure 2

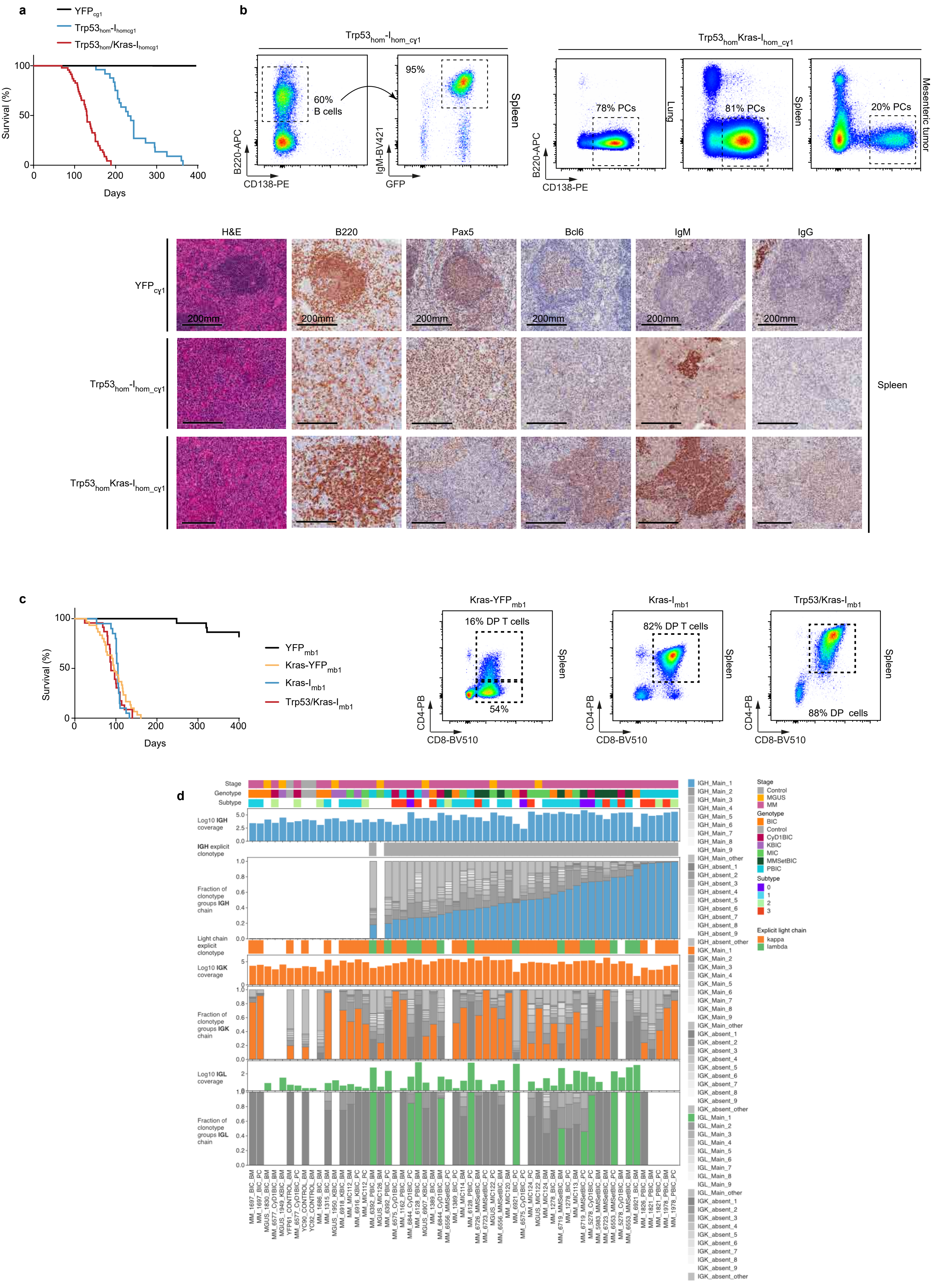

Supplementary Figure 2. Nine strains developed B-cell lymphoma, plasmablastic lymphoma, and acute lymphoblastic leukemia. a) Kaplan-Meier survival curves in two models that developed B-cell lymphoma: Trp53$_{hom}$-I$_{homc\gamma1}$ mice and Trp53$_{hom}$/Kras-I$_{homc\gamma1}$ mice. b) Representative flow cytometry and immunohistochemical analyses in these strains. c) Kaplan-Meier survival curves of three different models using the mb1-cre mice that developed T-cell acute lymphoblastic leukemia (left). Representative flow cytometry analyses of spleen samples are shown on the right. d) Clonotype abundance for each sample, including the coverage of heavy, kappa and lambda chains separately in each sample, fractions of clonotype groups, and the presence of explicit clonotypes according to the classifier (complementary to Figure 1l).

Supplementary Figure 3

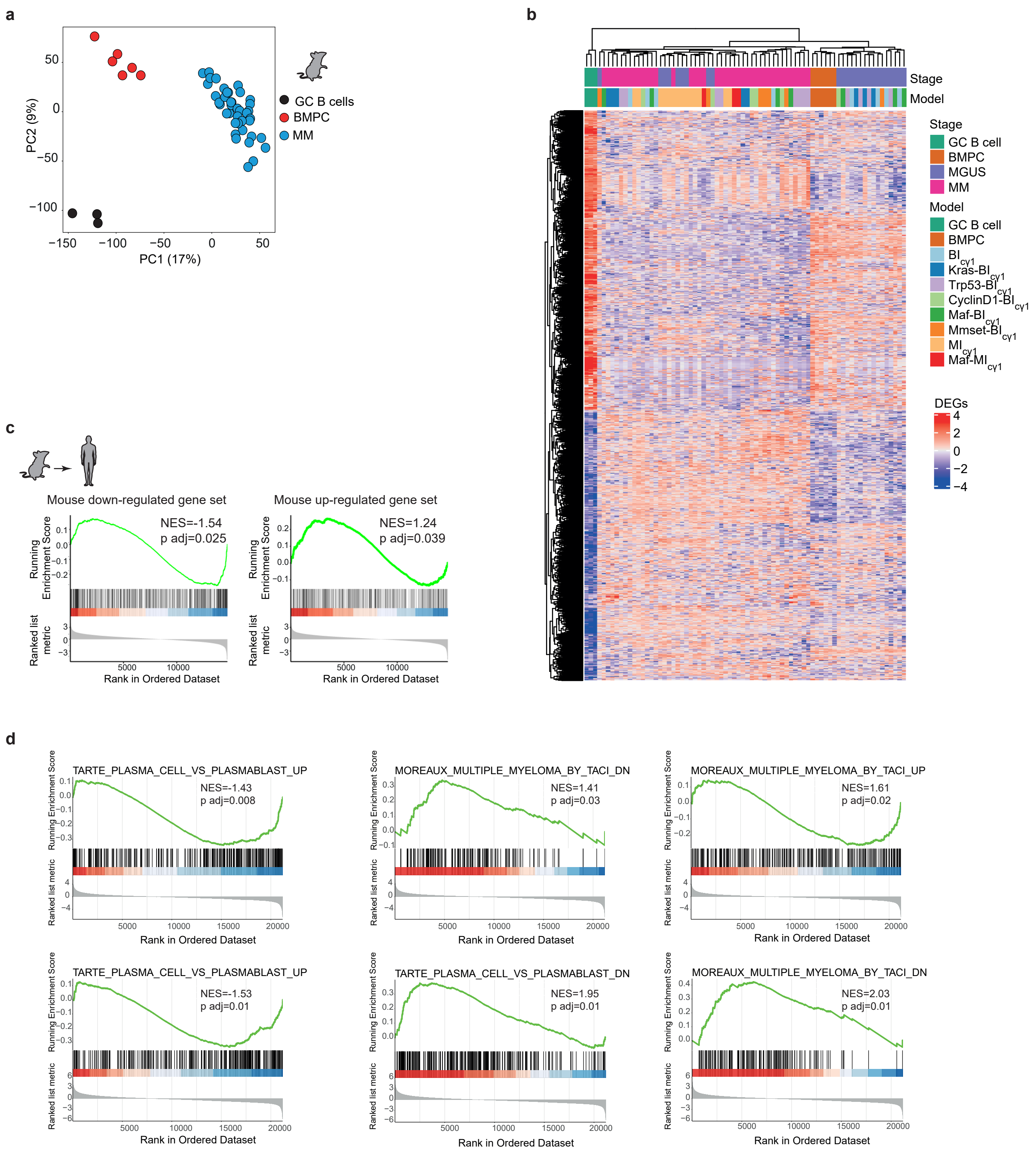

Supplementary Figure 3. a) PCA of mouse MM transcriptomes, which separates MM cells (n=38) from GC B cells (n=3) and PCs (n=6). The samples used are those characterized in Fig.2a. b) Unsupervised clustering analyses of the RNA-seq data corresponding to these samples, also including MGUS samples (n=24). DEGs, differentially expressed genes. c) Gene set enrichment analysis (GSEA) showing enrichment of mouse deregulated genes (down and up regulated) in the human MM transcriptome. NES, normalized enrichment score. d) Gene set enrichment analysis (GSEA) showing transcriptional similarities between Tarte and Moreaux datasets from *in vitro* human MM models and the MM mouse models.

Supplementary Figure 4

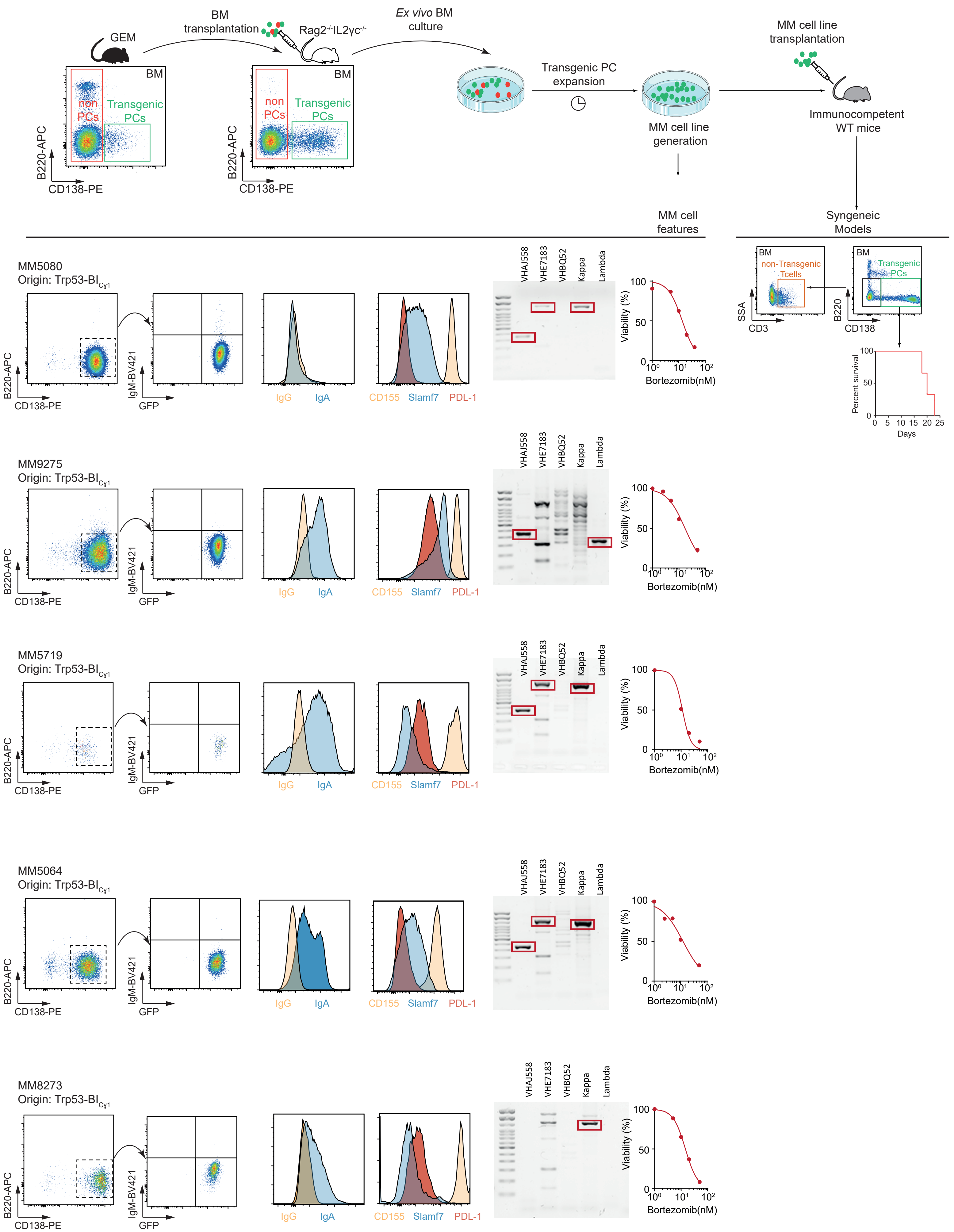

Supplementary Figure 4. Representation of MM cell line establishment from primary MM cells.

Supplementary Figure 5

MGUS sample (n=3)

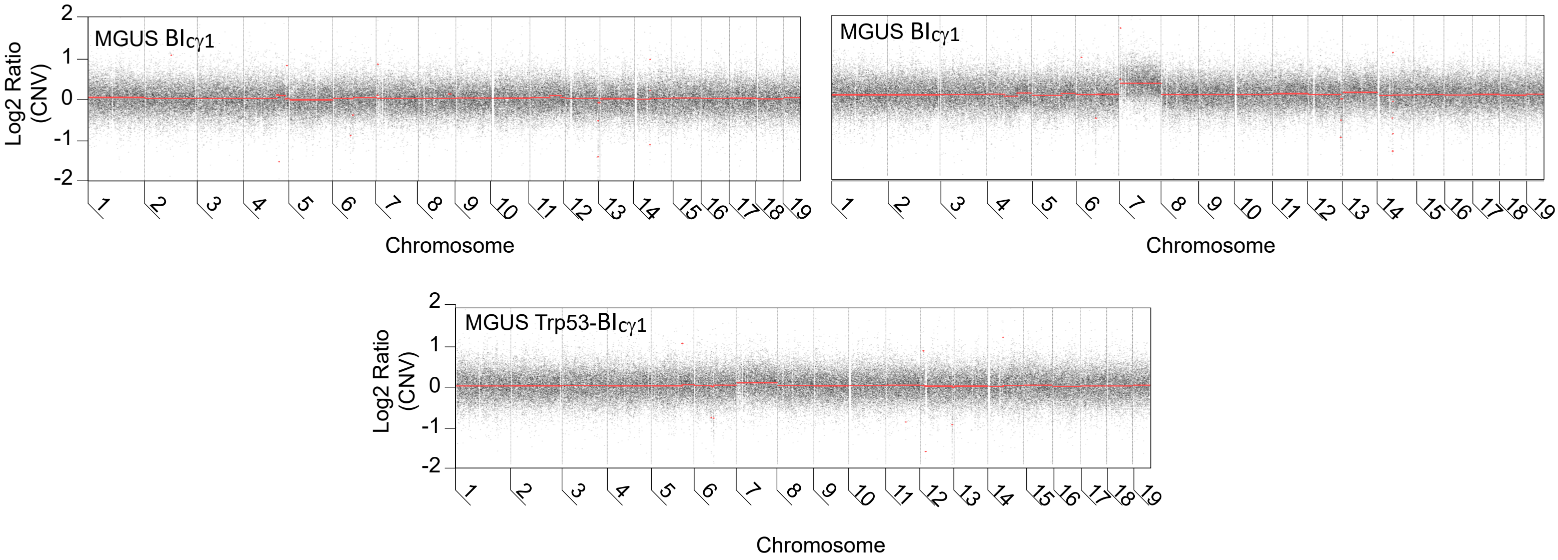

MM sample (n=62)

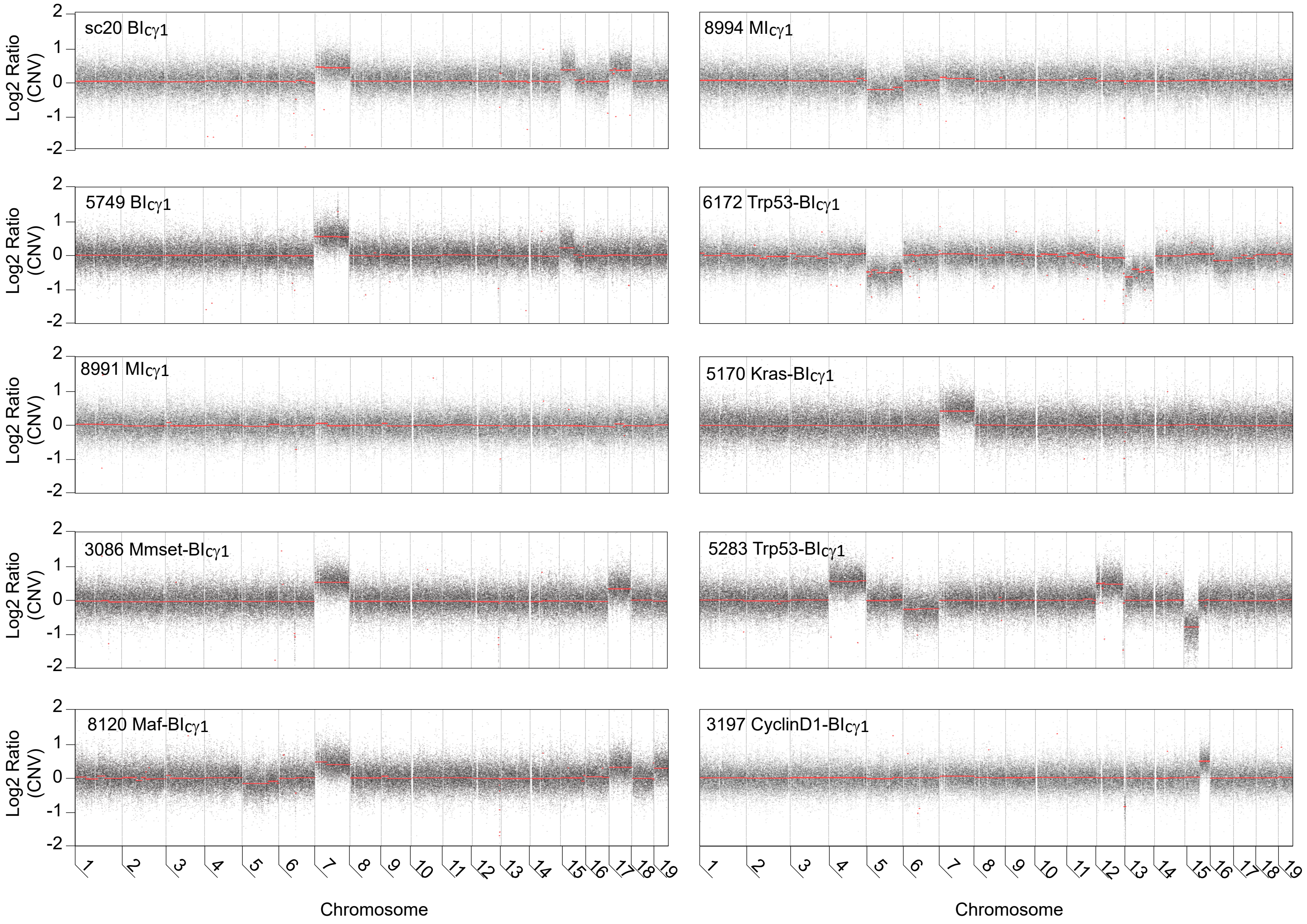

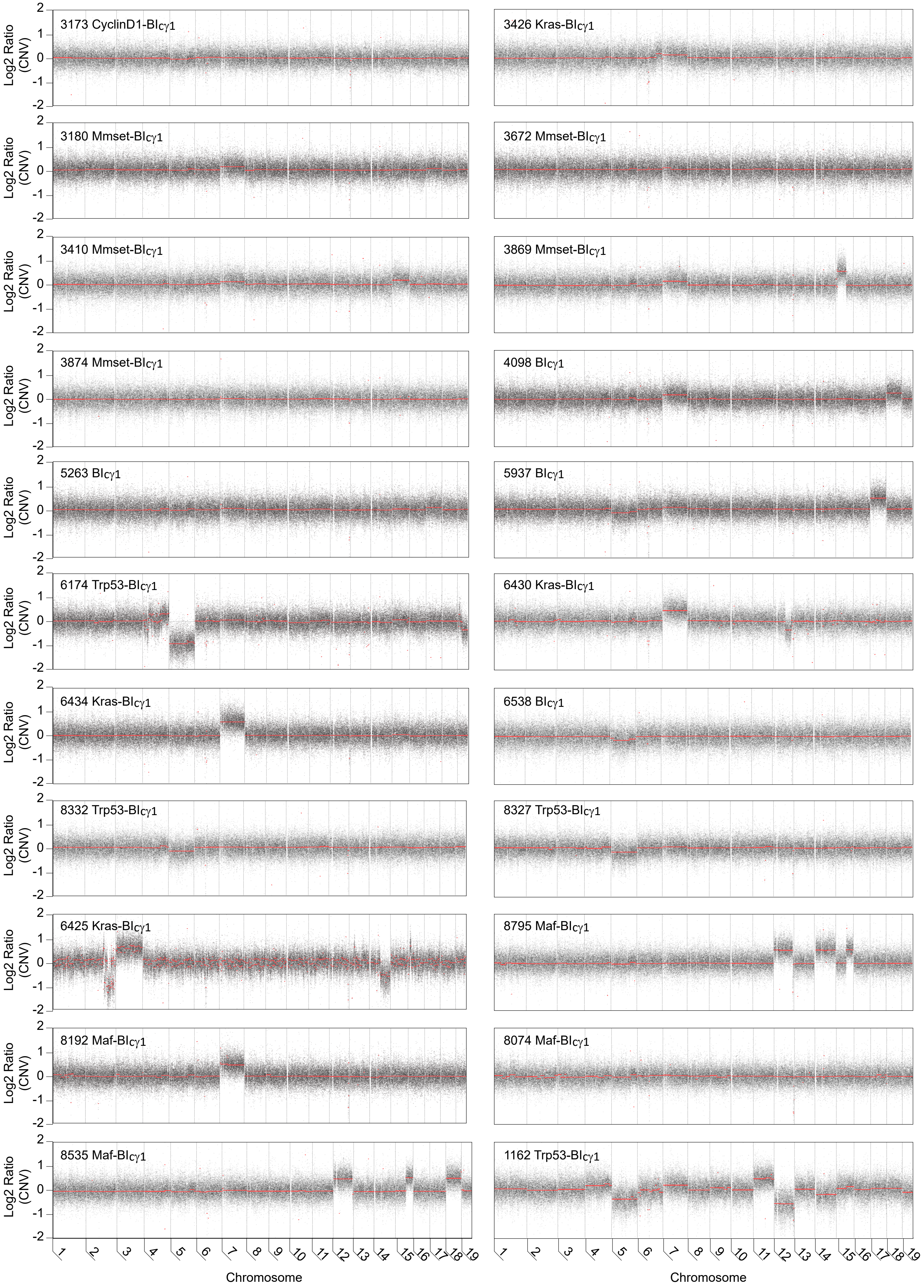
Log2 Ratio (CNV)
2
1
0
-1
-2
3173 CyclinD1-BIcγ1
3426 Kras-BIcγ1
3180 Mmset-BIcγ1
3672 Mmset-BIcγ1
3410 Mmset-BIcγ1
3869 Mmset-BIcγ1
3874 Mmset-BIcγ1
4098 BIcγ1
5263 BIcγ1
5937 BIcγ1
6174 Trp53-BIcγ1
6430 Kras-BIcγ1
6434 Kras-BIcγ1
6538 BIcγ1
8332 Trp53-BIcγ1
8327 Trp53-BIcγ1
6425 Kras-BIcγ1
8795 Maf-BIcγ1
8192 Maf-BIcγ1
8074 Maf-BIcγ1
8535 Maf-BIcγ1
1162 Trp53-BIcγ1
1 2 3 4 5 6 7 8 9 10 11 12 13 14 15 16 17 18 19
Chromosome

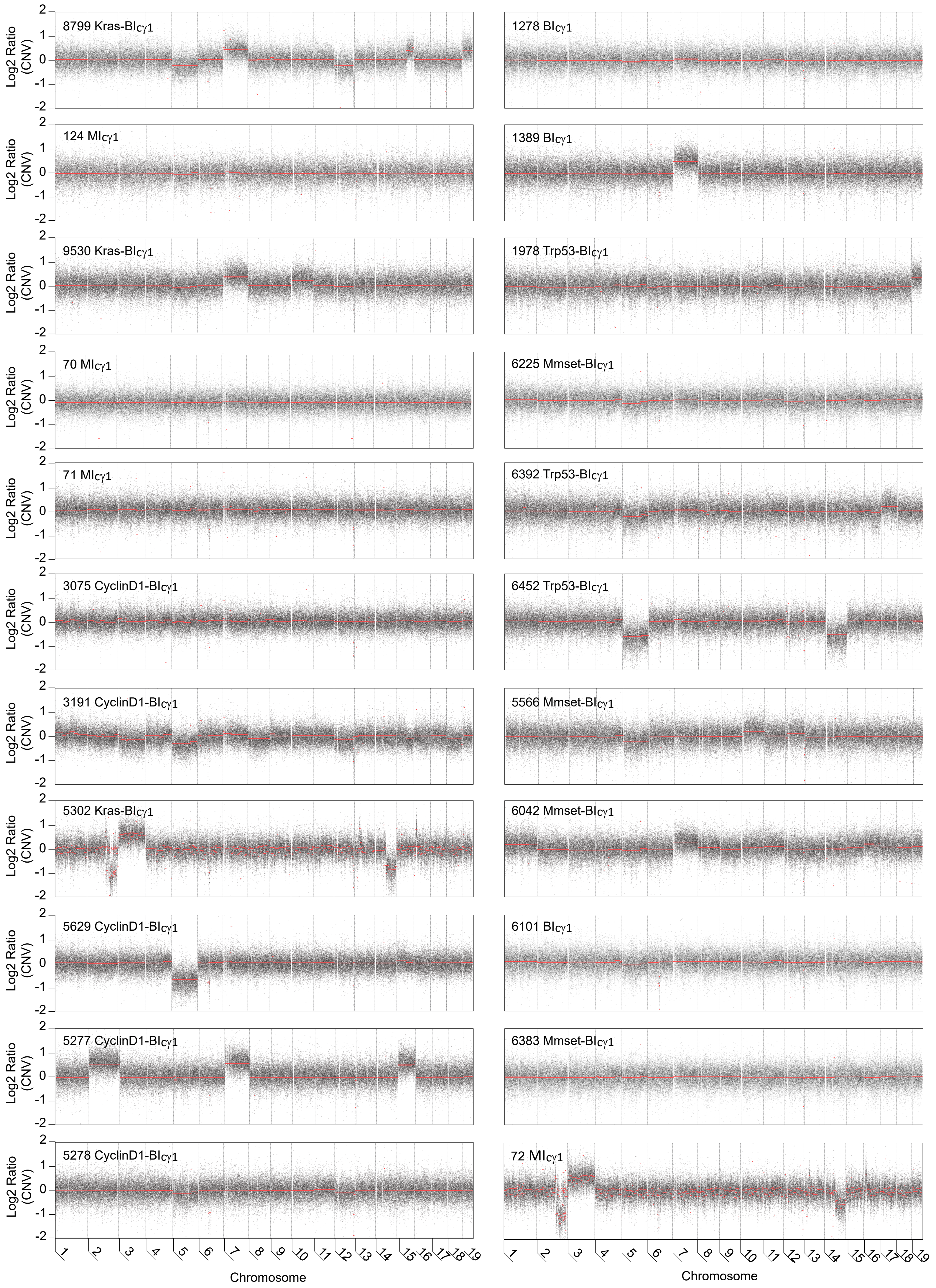
8799 Kras-BI$_{C\gamma1}$
1278 BI$_{C\gamma1}$
124 MI$_{C\gamma1}$
1389 BI$_{C\gamma1}$
9530 Kras-BI$_{C\gamma1}$
1978 Trp53-BI$_{C\gamma1}$
70 MI$_{C\gamma1}$
6225 Mmset-BI$_{C\gamma1}$
71 MI$_{C\gamma1}$
6392 Trp53-BI$_{C\gamma1}$
3075 CyclinD1-BI$_{C\gamma1}$
6452 Trp53-BI$_{C\gamma1}$
3191 CyclinD1-BI$_{C\gamma1}$
5566 Mmset-BI$_{C\gamma1}$
5302 Kras-BI$_{C\gamma1}$
6042 Mmset-BI$_{C\gamma1}$
5629 CyclinD1-BI$_{C\gamma1}$
6101 BI$_{C\gamma1}$
5277 CyclinD1-BI$_{C\gamma1}$
6383 Mmset-BI$_{C\gamma1}$
5278 CyclinD1-BI$_{C\gamma1}$
72 MI$_{C\gamma1}$
Log2 Ratio (CNV)
2
1
0
-1
-2
1 2 3 4 5 6 7 8 9 10 11 12 13 14 15 16 17 18 19
Chromosome

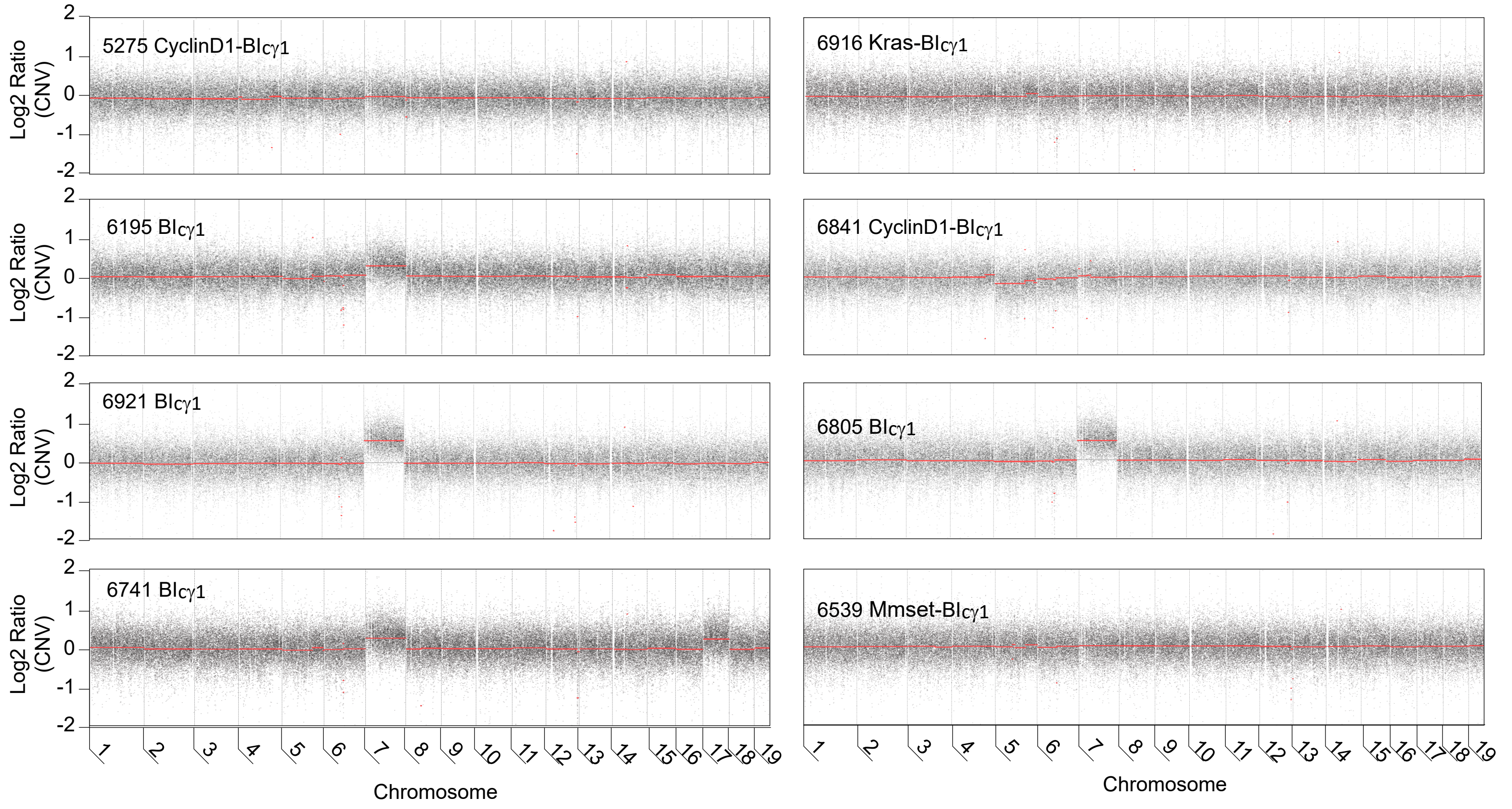

MM-derived cell lines (n=6)

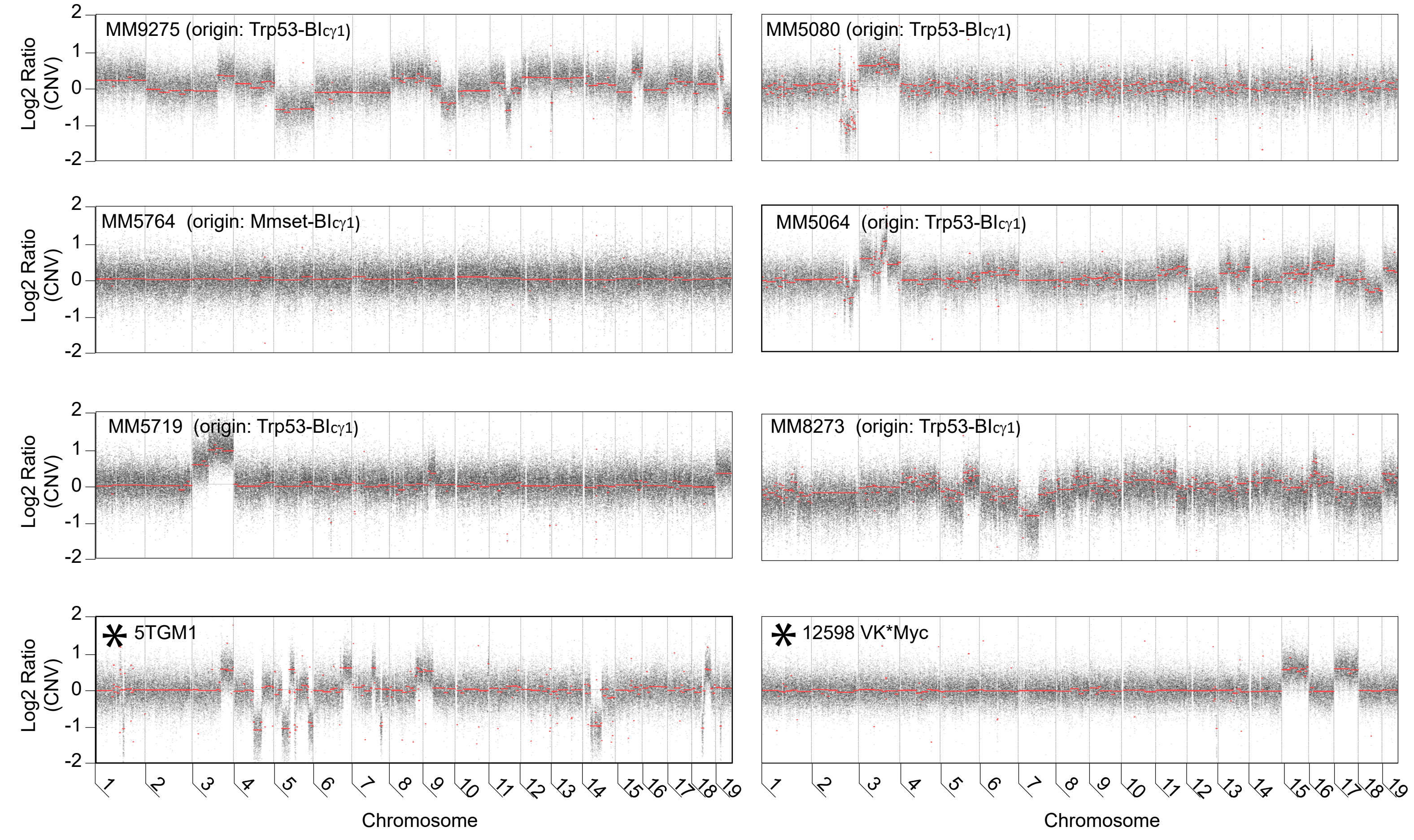

* Previously stablished MM-derived cell lines

Supplementary Figure 5. CNV in 62 murine samples from the MM stage, 3 samples of pooled PCs from 9 mice at the MGUS stage, and 6 samples from MM-derived cell lines. In addition, the 5TGM1 and 12598-Vk*MYC cell lines were analyzed.

Supplementary Figure 6

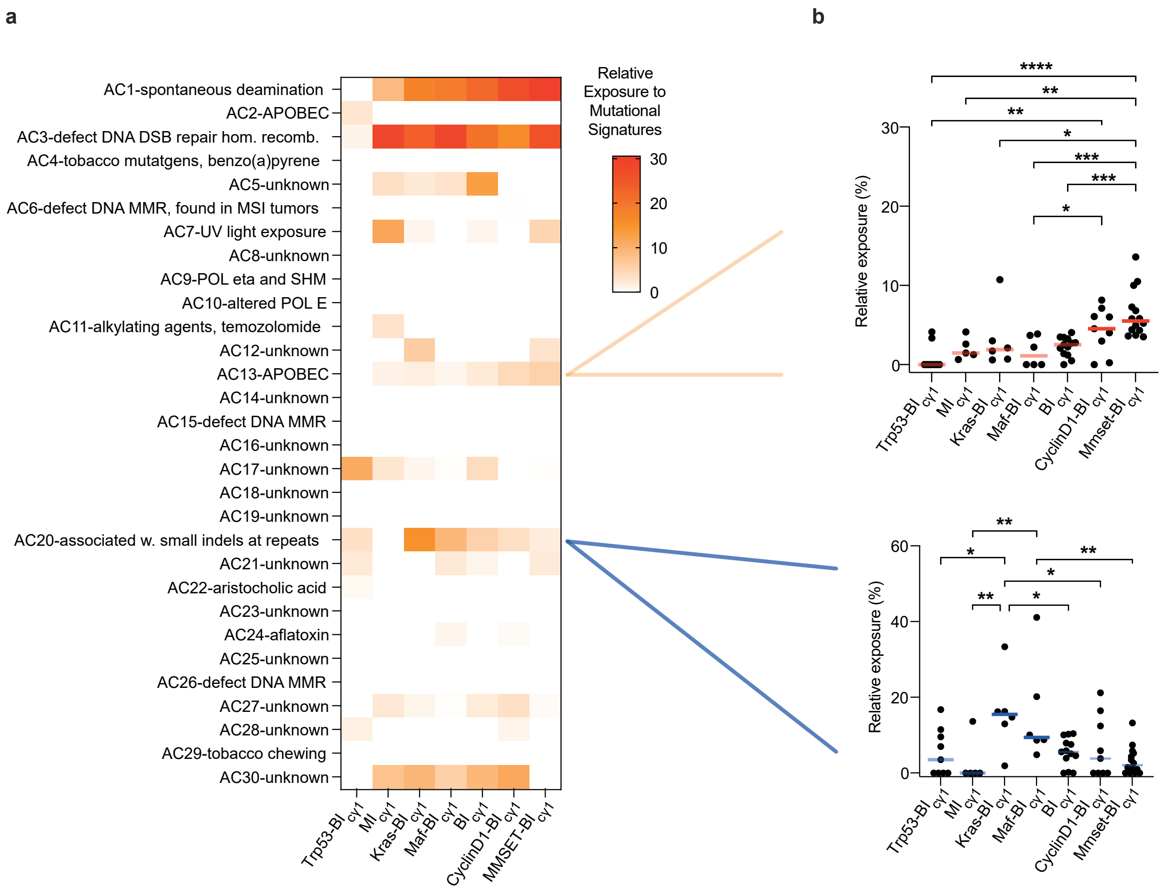

Supplementary Figure 6. Contribution of mutational signatures (according to Alexandrov et al. Nature 2013) to MM in mice. a) Heatmap showing median values in each MM mouse group (n≥6 animals per group) of the relative contribution of COSMIC mutational signatures. Single nucleotide variants (SNV) detected by WES in autosomal chromosomes were used for mutational signature analysis with the R package YAPSA. b) Distribution of relative exposures to signatures 13 (AC13) and 20 (AC20) in the cohort of MM samples, which have been attributed respectively to the AID/APOBEC family of cytidine deaminases or to defective DNA mismatch repair associated with small insertions and deletions. The mechanistic basis of some signatures remains speculative or unknown (labeled as unknown).

Supplementary Figure 7

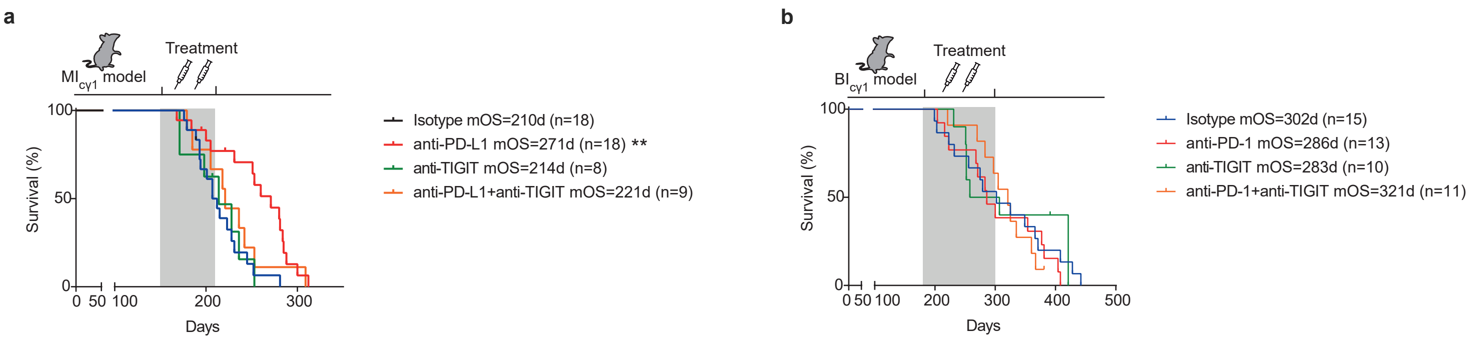

Supplementary Figure 7. a) Pre-clinical immunotherapy trial in $MI_{c\gamma1}$ mice. Four therapy cohorts received anti-PD-L1 (n=18), anti-TIGIT (n=8), or both anti-PD-L1 and anti-TIGIT (n=9) moAbs for 8 weeks beginning at 4 months of age, while control mice (n=18) were treated with rat IgG control antibody at similar intervals. Measurement of overall survival in the different treatment cohorts was determined in Kaplan–Meier curves. In the figure, median OS (mOS) values are shown. A statistically significant extension of survival was observed with the combination therapy with respect to the other therapy groups. b) Pre-clinical immunotherapy trial in $BI_{c\gamma1}$ mice. Four therapy cohorts received anti-PD-1 (n=13), anti-TIGIT (n=10), or both anti-PD-1 and anti-TIGIT (n=11) moAbs for 8 weeks beginning at 6 months of age, while control mice (n=15) were treated with rat IgG control antibody at similar intervals. Kaplan–Meier survival curves did not show statistically significant differences among the treatment cohorts.
