# Supplementary material for: Preclinical models for prediction of immunotherapy outcomes and immune evasion mechanisms in genetically heterogeneous multiple myeloma
Source: Nat Med. 2023 Mar 16;29(3):632–45. doi: 10.1038/s41591-022-02178-3 (PMC10033443; doi:10.1038/s41591-022-02178-3)
Supplement: Supplementary file 2 — Reporting Summary [file 41591_2022_2178_MOESM2_ESM.pdf]

nature portfolio

Corresponding author(s): Jose A Martinez-Climent

Last updated by author(s): Dec

Reporting Summary

Nature Portfolio wishes to improve the reproducibility of the work that we publish. This form provides structure for consistency and transparency in reporting. For further information on Nature Portfolio policies, see our Editorial Policies and the Editorial Policy Checklist.

Statistics

For all statistical analyses, confirm that the following items are present in the figure legend, table legend, main text, or Methods section.

n/a	Confirmed	
☐	☒	The exact sample size (*n*) for each experimental group/condition, given as a discrete number and unit of measurement
☒	☐	A statement on whether measurements were taken from distinct samples or whether the same sample was measured repeatedly
☐	☒	The statistical test(s) used AND whether they are one- or two-sided<br>*Only common tests should be described solely by name; describe more complex techniques in the Methods section.*
☒	☐	A description of all covariates tested
☐	☒	A description of any assumptions or corrections, such as tests of normality and adjustment for multiple comparisons
☐	☒	A full description of the statistical parameters including central tendency (e.g. means) or other basic estimates (e.g. regression coefficient) AND variation (e.g. standard deviation) or associated estimates of uncertainty (e.g. confidence intervals)
☐	☒	For null hypothesis testing, the test statistic (e.g. *F*, *t*, *r*) with confidence intervals, effect sizes, degrees of freedom and *P* value noted<br>*Give P values as exact values whenever suitable.*
☒	☐	For Bayesian analysis, information on the choice of priors and Markov chain Monte Carlo settings
☒	☐	For hierarchical and complex designs, identification of the appropriate level for tests and full reporting of outcomes
☐	☒	Estimates of effect sizes (e.g. Cohen's *d*, Pearson's *r*), indicating how they were calculated

Our web collection on statistics for biologists contains articles on many of the points above.

Software and code

Policy information about availability of computer code

Data collection	Flow cytometry data was collected using FACS Canto II cytometer or FACS Aria sorter using the software FACSDiva v6.1.3<br>RNAseq data were sequenced on an Illumina NextSeq500 and on an IlluminaHiSeq2500.<br>WES and WGS data were obtained after sequencing on an Illumina Novaseq600.<br>Public RNAseq and microarray datasets from multiple myeloma patients were included (GSE136324 and GSE104171)
Data analysis	For flow cytometry data analysis FlowJo v10.7.1, Infinicyt v2.0.5 and a semi-automated pipeline "FlowCT" (Botta C, Blood Advances,2021) were used.<br>RNAseq, WES and WGS analysis was performed in R software v3.6.1, using following packages Limma and cluster profiler (3.14.3) . For RNAseq analysis Illumina bcl2fastq v 2.20.0, STAR aligner v2.61, HTseq v0.11.0 and the R package clusterProfiler v3.14.3 were employed.<br>scRNAseq data were processed and analyzed with the package Seurat (https://satijalab.org/seurat/)<br>WES data analysis was performed using a custom pipeline dseigned by Dreamgenics S.L.<br>For bone reconstruction Quantum 3.0 software was used and spectral karotiping was analysed with HiSKY software<br>WGS data were analyzed using the HMMcopy adaptation of CopywriteR.<br>GraphPrism software v9.0 was employed for plotting and statistical analysis. SPSS v.25 software was used for COX regression analysis

For manuscripts utilizing custom algorithms or software that are central to the research but not yet described in published literature, software must be made available to editors and reviewers. We strongly encourage code deposition in a community repository (e.g. GitHub). See the Nature Portfolio guidelines for submitting code & software for further information.

Data

Policy information about availability of data

All manuscripts must include a data availability statement. This statement should provide the following information, where applicable:

- Accession codes, unique identifiers, or web links for publicly available datasets
- A description of any restrictions on data availability
- For clinical datasets or third party data, please ensure that the statement adheres to our policy

Raw sequencing data was deposited on Gene Expression Omnibus with the following accession codes: GSE205447 (RNAseq data from mouse and human MGUS and MM samples and control mice and healthy donors); GSE205644 (bulk RNAseq data from mouse BM samples at MGUS and MM stages and control mice); GSE220997 (scRNAseq and TCR-RNAseq data from T-cells isolated from mice and patients and WES and WGS raw data form mouse samples and mouse cell lines).

Human research participants

Policy information about studies involving human research participants and Sex and Gender in Research.

Reporting on sex and gender	Patients from both sexes were collected.
Population characteristics	Healthy donors and myeloma patients of both sexes and aged between 18 and 99 years were recruited for this study.
Recruitment	Samples were collected from September 2013 until November 2021
Ethics oversight	This study was performed in accordance with the regulations of the Institutional Review Board of the University of Navarra and was conducted according to the principles of the Declaration of Helsinki. Informed consent was obtained from all patients.

Note that full information on the approval of the study protocol must also be provided in the manuscript.

Field-specific reporting

Please select the one below that is the best fit for your research. If you are not sure, read the appropriate sections before making your selection.

☒ Life sciences ☐ Behavioural & social sciences ☐ Ecological, evolutionary & environmental sciences

For a reference copy of the document with all sections, see nature.com/documents/nr-reporting-summary-flat.pdf

Life sciences study design

All studies must disclose on these points even when the disclosure is negative.

Sample size	Sample size was determined according to previous variability observed in similar studies in this field.<br>In general, a minimum of 3 and up to 10 samples for each mouse genotype and disease stage were included. For human samples, the sample size was limited by the availability of subjects and the patients enrolled in the PETHEMA/GEM-CLARIDEX clinical trial
Data exclusions	No data were excluded from the analysis
Replication	Replication data was not performed for RNAseq, WES, WGS and scRNAseq, due to cost and availability of material.<br>For in vitro experiments, a minimum of four independent replicates were performed, with at least three concordant attemps. Immunoblot assays were repeated at least two times per cell line. Flow cytometry analysis was performed in a minimum of 10 samples per mouse genotype and disease stage.<br>In vivo experiments in syngeneic mouse models were repeated twice. Pre-clinical trials in multiple myeloma models were not replicate, due to time.
Randomization	Age-matched mice were randomly distributed into experimental groups. Mice of both sexes were equally allocated into treatment groups.
Blinding	Blinding was not applicable to this study.

Reporting for specific materials, systems and methods

We require information from authors about some types of materials, experimental systems and methods used in many studies. Here, indicate whether each material, system or method listed is relevant to your study. If you are not sure if a list item applies to your research, read the appropriate section before selecting a response.

Materials & experimental systems

n/a	Involved in the study
☐	☒ Antibodies
☐	☒ Eukaryotic cell lines
☒	☐ Palaeontology and archaeology
☐	☒ Animals and other organisms
☐	☒ Clinical data
☒	☐ Dual use research of concern

Methods

n/a	Involved in the study
☒	☐ ChIP-seq
☐	☒ Flow cytometry
☒	☐ MRI-based neuroimaging

Antibodies

Antibodies used

Rabbit anti-Bcl-2 Santa Cruz Biotechnology Cat#sc-783; RRID:AB_2243455
Rabbit anti-Bcl-xL R&D Systems Cat#MAB894
Rabbit anti-p44/42 MAPK(ERK1/2) Cell Signaling Technology Cat#9102; RRID: AB_330744
Rabbit anti-Phospho-p44/42 MAPK(ERK1/2) Cell Signaling Technology Cat#4376; RRID: AB_331772
Rabbit anti-MCL1 Abcam Cat#Ab32087; RRID:AB_776245
Rabbit anti-c-Myc Abcam Cat#Ab32072; RRID:AB_731658
Rabbit anti-c-Myc (phospho T58) Abcam Cat#ab185655
Rabbit anti-c-Myc (phospho S62) Abcam Cat#ab185656
Mouse anti-Actin Calbiochem Cat#CP01; RRID:AB_566293
Mouse anti-α-Tubulin SigmaAldrich Cat#T6074; RRID: AB_477582
Donkey anti-Rabbit IgG-HRP Amersham Cat#NA934; RRID:AB_772206
Sheep anti-Mouse IgG-HRP Amersham Cat#NA931; RRID:AB_772210
Rabbit anti-rat IgG(H+L) Vector Laboratories Cat#BA-4001; RRID: AB_10015300
PE/CY7 anti-mouse CD3 BioLegend Cat#100220; RRID:AB_1732057
APC anti-mouse CD4 BioLegend Cat#100516; RRID:AB_312719
Pacific Blue anti-mouse CD4 BioLegend Cat#116008; RRID:AB_11149680
Brilliant Violet 510 anti-mouse CD8a BioLegend Cat#100752; RRID:AB_2563057
APC/Cy7 anti-mouse CD19 BioLegend Cat#115530; RRID:AB_830707
PE-Cy7 Mouse anti-Human CD19 BD Biosciences Cat#560728; RRID:AB_1727438
Brilliant Violet 510 anti-mouse CD25 BioLegend Cat#102041; RRID:AB_2562269
FITC Mouse anti-Human CD38 BD Biosciences Cat#555459; RRID:AB_395852
APC/Fire 750 anti-mouse/human CD44 BioLegend Cat#103061; RRID:AB_2616726
PerCPCy5.5 Mouse anti-Human CD45 BD Biosciences Cat#564105; RRID:AB_2744405
APC anti-mouse/human CD45R/B220 BioLegend Cat#103212; RRID:AB_312997
PE Mouse anti-Human CD56 BD Biosciences Cat#555516; RRID:AB_395906
PE anti-mouse CD62L BioLegend Cat#104407; RRID:AB_313094
APC-H7 Mouse anti-Human CD81 BD Biosciences Cat#656647; RRID:AB_2565861
APC Mouse anti-Human CD117 BD Biosciences Cat#550412; RRID:AB_398461
APC anti-mouse CD138 BD Biosciences Cat#561705; RRID:AB_1645216
Brilliant Violet 421 Mouse anti-Human CD138 BD Biosciences Cat#562935; RRID:AB_2737904
PE anti-mouse CD138 (Syndecan-1) BioLegend Cat#142504; RRID:AB_10916119
APC anti-mouse CD223 (LAG-3) BioLegend Cat#125209; RRID:AB_10639935
PE/Cy7 anti-mouse CD223 (LAG-3) BioLegend Cat#125226; RRID:AB_2715764
PE/Cy7 anti-mouse CD226 (DNAM-1) BioLegend Cat#133625; RRID:AB_2716223
anti-mouse CD267 (TACI)-APC Miltenyi Biotec Cat#130-103-361; RRID:AB_2656776
PE Rat anti-mousse CD274 (PD-L1) BD Biosciences Cat#558091; RRID:AB_397018
Brilliant Violet 421 anti-mouse CD279 (PD-1) BioLegend Cat#135218; RRID:AB_2561447
Brilliant Violet 510 anti-mouse CD279 (PD-1) BioLegend Cat#135241; RRID:AB_2715761
PE anti-mouse CD319 BioLegend Cat#152005; RRID:AB_2632676
Brilliant Violet 421 anti-mouse IgM BioLegend Cat#406518; RRID:AB_2561444
Brilliant Violet 421 anti-mouse NK-1.1 BioLegend Cat#108731; RRID:AB_10895916
PE anti-mouse TIGIT (Vstm3) BioLegend Cat#142103; RRID:AB_10895760
PE/Cy7 anti-mouse TIGIT (Vstm3) BioLegend Cat#142108; RRID:AB_2565649
PE anti-mouse Foxp3 eBiosciences Cat#12-5776-82; RRID: AB_465936

Validation

Flow cytometry antibodies from BioLegend, Miltenyi and BD biosciences were validated for manufactures. Additionally, they were evaluated in spleen and bone marrow samples of C57BL/6 mice.
Western-blot and immunohistochemistry antibodies were validated for manufactures. Rabbit anti-cMYC (phopshoS62) antibody from Abcam was tested in a CRISPR knock out cMYC murine cell line by Western-blot.
In vivo monoclonal antibodies have been widely used in the last years for the scientific community. The antiCD25 antibody (clone 7D4) was extensively validated by Roche (Solomo et al. Nat Cancer1, 1153-1166;2020)

Eukaryotic cell lines

Policy information about cell lines and Sex and Gender in Research

Cell line source(s)

RPMI8226; DSMZ; Cat# ACC-402; RRID: CVCL_2989
KMS12; DSMZ; Cat# ACC-551; RRID: CVCL_1334
KMS26; Takemi O et al., Gene Funct Dis. 2000; RRID: CVCL_2992.

	KMS11; Namba M et al., In Vitro Cell Dev Biol. 1989; RRID: CVCL_2989<br>MM1S; ATCC; Cat#CRL-2974; RRID: CVCL_8792<br>U266; DSMZ; Cat#ACC-9; RRID: CVCL_0566<br>K620; DSMZ; Cat#ACC-514; RRID: CVCL_1823<br>JJN3; DSMZ; Cat#ACC-541; RRID: CVCL_2078<br>H929; DSMZ; Cat#ACC-163; RRID: CVCL_1600<br>MOLP2; DSMZ; Cat#ACC-607, RRID: CVCL_2123<br>5TGM1; Garrett IR et al., Bone 1997; RRID: CVCL_VI66. Provided by Sandra Hervás (CIMA, Spain)<br>Vk12598; Meermeier EW et al., Blood 2021. Provided by Leif Bergsagel (Mayo Clinic, Arizona)<br>In this paper we generated eight new multiple myeloma murine cell lines: MM9275, MM8273, MM5080, MM5064, MM5719, MM6452, MM5764 and MM2732.
Authentication	Commercial cell lines were authenticated by PCR based STR fingerprinting.
Mycoplasma contamination	Cell lines were tested every two weeks for mycoplasma contamination using the MycoAlert Mycoplasma Detection Kit. Cells were discarded if the test was positive.
Commonly misidentified lines<br>(See ICLAC register)	No commonly misidentified cell lines were used in this study

Animals and other research organisms

Policy information about studies involving animals; ARRIVE guidelines recommended for reporting animal research, and Sex and Gender in Research

Laboratory animals	The following commercial mouse strains were used in this study:<br>B6(Cg)-Gt(ROSA)26Sortm4(Ikbkb)Rsky/J; The Jackson Laboratory; Strain code:008242<br>129S/Sv-Krastm4Tyj/J; The Jackson Laboratory; Strain code:008180<br>B6.Cg-Tg(BCL2)22Wehi/J; The Jackson Laboratory; Strain code:002319<br>C57BL/6N-Gt(ROSA)26Sortm13(CAGMYC,-CD2*)Rsky/J; The Jackson Laboratory; Strain code:020458<br>B6.129P2-Trp53tm1Brn/J; The Jackson Laboratory; Strain code:008462<br>B6.129P2(Cg)-Ighg1tm1(cre)Cgn/J; The Jackson Laboratory; Strain code:010611<br>B6.129X1-Gt(ROSA)26Sortm1(EYFP)Cos/J; The Jackson Laboratory; Strain code:006148<br>129S4-Rag tm1.1Flv/I2rgtm1.1Flv /J; The Jackson Laboratory; Strain code:014593<br>B6.129(Cg)-Foxp3tm3(DTR/GFP)Ayr/J; The Jackson Laboratory; Strain code:016958<br>BXSB.B6-Tg(TcraTcrb)1100Mjb/DcrJ; The Jackson Laboratory; Strain code: 021880<br>C57BL/6JOlaHsd; Envigo; Strain code:057C<br>The mouse models listed below were kindly provided by collaborators.<br>B6.Cg-Tg (Eµ-CCND1) Katz SG et al., Blood 2014<br>B6.Cg-Tg (Eµ-C-Maf) Morito N et al., Cancer Res. 2011<br>B6.C(Cg)-Cd79atm1(cre)Reth/EhobJ Hobeika et al. PNAS 2006<br>VK*MYC Chesi M et al., Cancer Cell 2008<br>The Rosa26-hMMSET-IIStop-Floxed mice were generated for this study following the protocol detailed in material and methods of the manuscript.<br>Trasngenic mice were studied until signs of disease appeared.<br>Syngeneic mouse models were conducted in mice between 8-12 weeks-old.<br>Mice were kept under specific pathogen-free conditions and light/dark cycles of 12h. The temperature was constantly mantained between 18-23ºC.
Wild animals	This study did not involve wild animals
Reporting on sex	Mice from both sexes were used. Mice of both sexes were randonly assiganted to therapy groups.
Field-collected samples	This study did not involve samples collected in the field
Ethics oversight	Animals used in this study were kept under specific pathogen-free conditions in the animal facilities of the Center for Applied Medical Research CIMA at the University of Navarra. Animal experimentation was approved by the Ethical Committee of Animal Experimentation of the University of Navarra and by the Health Department of the Navarra Government.

Note that full information on the approval of the study protocol must also be provided in the manuscript.

Clinical data

Policy information about clinical studies
All manuscripts should comply with the ICMJE guidelines for publication of clinical research and a completed CONSORT checklist must be included with all submissions.

Clinical trial registration	ClinicalTrails.gov NCT02575144
Study protocol	The full protocol can be found on https://clinicaltrials.gov/ct2/show/NCT02575144
Data collection	Multiple myeloma patients addressing the inclusion criteria of the trial were enrolled from July 2015 to May 2019. Data collection will be registered until October 2024. Location: Spain and US

Outcomes	The primary outcome of this phase III clinical trial is to assess progression free survival in multiple myeloma patients ineligible for ASCT, receiving Rd with or without clarithromycin

Flow Cytometry

Plots

Confirm that:

☒ The axis labels state the marker and fluorochrome used (e.g. CD4-FITC).

☒ The axis scales are clearly visible. Include numbers along axes only for bottom left plot of group (a 'group' is an analysis of identical markers).

☒ All plots are contour plots with outliers or pseudocolor plots.

☒ A numerical value for number of cells or percentage (with statistics) is provided.

Methodology

Sample preparation	As described in methods, mouse cell suspensions from spleen (obtained by mechanical disruption) and bone marrow (flushed from femurs with DPBS) were filtered through a 70-µm cell strainer (Falcon) and treated with ACK lysis buffer to remove red blood cells. Then, cells were washed in DPBS and filtered a second time before they were labeled with antibodies for flow cytometric analysis.<br>Human samples were obtained by bone marrow aspiration and processed using a standard EuroFlow lyse-wash and stain protocol.
Instrument	BD FACS Canto II was used for flow cytometry and FACS Aria II for cell sorting
Software	Data were analyzed in FlowJo v10.7.1
Cell population abundance	Different cell populations were measured by flow cytometry in mice and human samples
Gating strategy	All samples were FSC-A and FSC-H gated to select singlet cells, then FSC-A/SSC-A gating was applied to select mononucleated cells. Death cells were excluded using the viability marker 7AAD. Subsequent gating was conducted to select targeted populations.

☒ Tick this box to confirm that a figure exemplifying the gating strategy is provided in the Supplementary Information.
