# Supplementary material for: Preclinical models for prediction of immunotherapy outcomes and immune evasion mechanisms in genetically heterogeneous multiple myeloma
Source: Nat Med. 2023 Mar 16;29(3):632–45. doi: 10.1038/s41591-022-02178-3 (PMC10033443; doi:10.1038/s41591-022-02178-3)
Supplement: Supplementary file 4 — Unprocessed serum electrophoresis gels corresponding to gels in Fig. 1 and Extended Data Figs. 2 and 3. [file 41591_2022_2178_MOESM4_ESM.pdf]

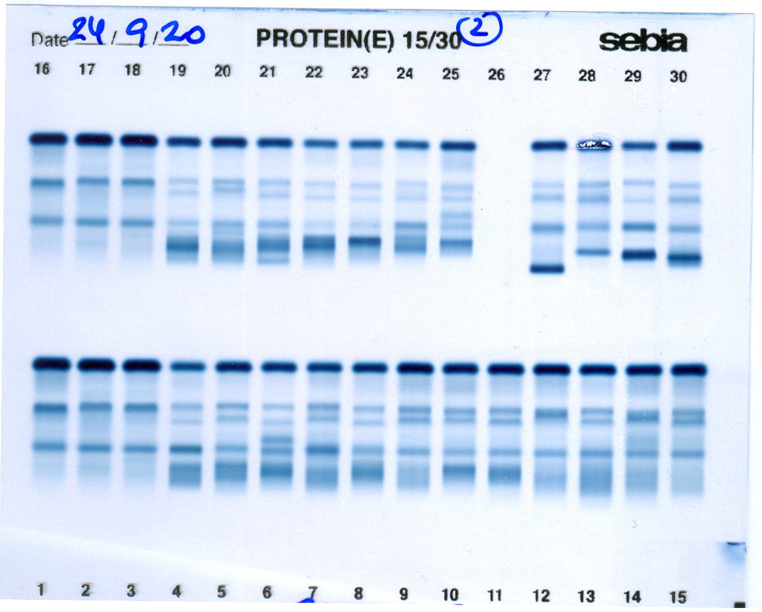

Figure 1f
YFPcy1: sample 3
Blcy1: sample 30
Mlcy1: sample 19
Mlmb1: sample 22

Extended Data Figure 2a
YFPcy1: sample 3
Blcy1: sample 29

Extended Data Figure 2b
YFPcy1: sample 3
Mlcy1: sample 19

Extended Data Figure 2c
YFPcy1: sample 3
Blcy1: sample 29
Mlcy1: sample 19

Extended Data Figure 3a
YFPcy1: sample 3
Blcy1: sample 29

Extended Data Figure 3b
YFPcy1: sample 3
Mlcy1: sample 19

Extended Data Figure 3c
YFPcy1: sample 3

Extended Data Figure 3d
YFPcy1: sample 3
Blcy1 MGUS: sample 14 and 15
Blcy1 MM: sample 29 and 30
Mlcy1 MGUS: sample 4 and 5
Blcy1 MM: sample 21 and 22

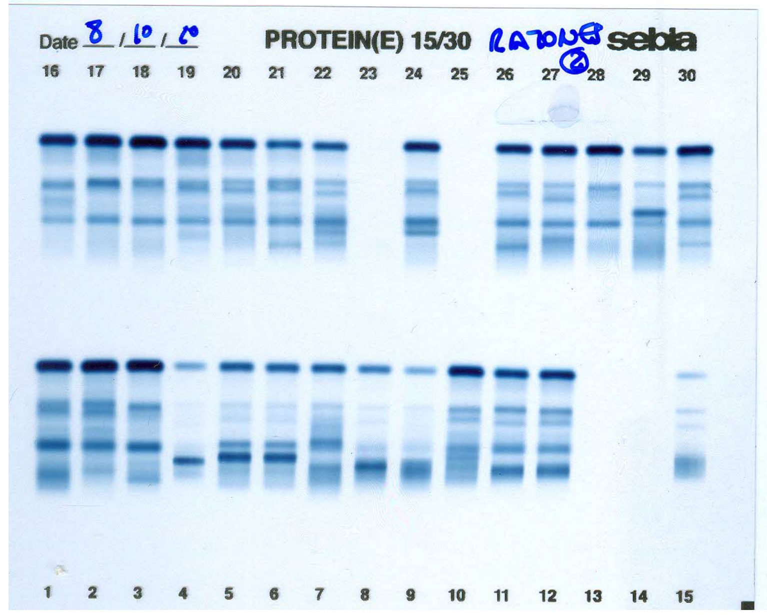

Extended Data Figure 2c
Bcl2-Mlcy1: sample 8

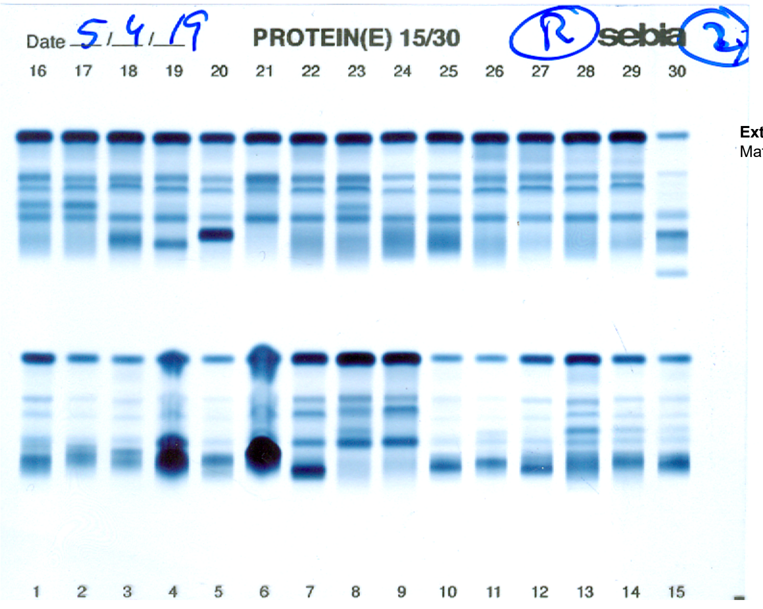

Extended Data Figure 3b
Maf-Mlcy1: sample 13

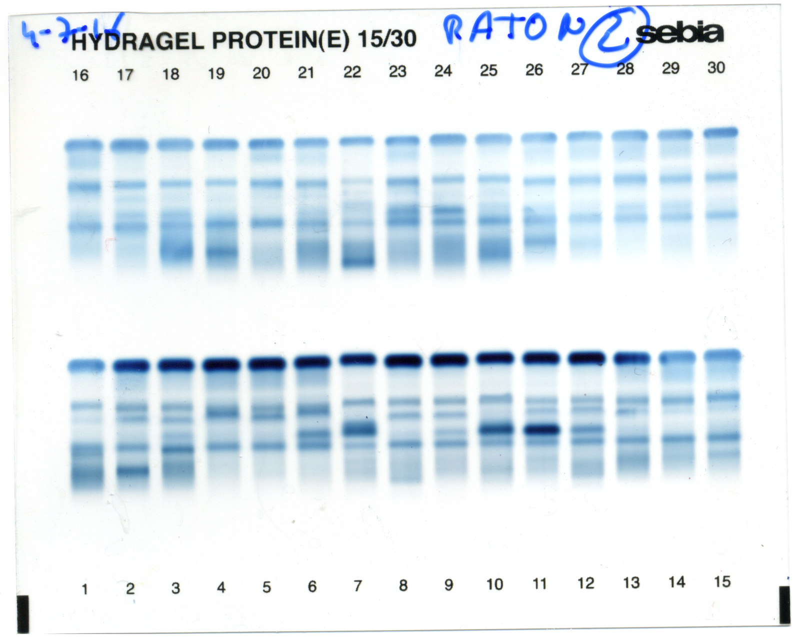

Extended Data Figure 3c
Icy1: sample 8

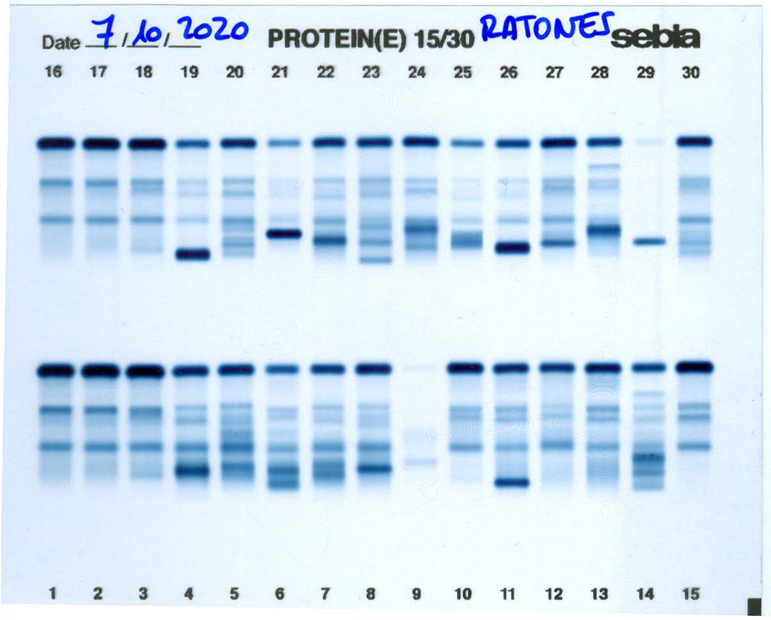

Extended Data Figure 2a
Kras-Blcy1: sample 4 and 5
Trp53-Blcy1: sample 8 and 11
Kras/Trp53-Blcy1: sample 19

Extended Data Figure 3a
Maf-Blcy1: sample 19
CyclinD1-Blcy1: sample 26
Mmset-Blcy1: sample 28

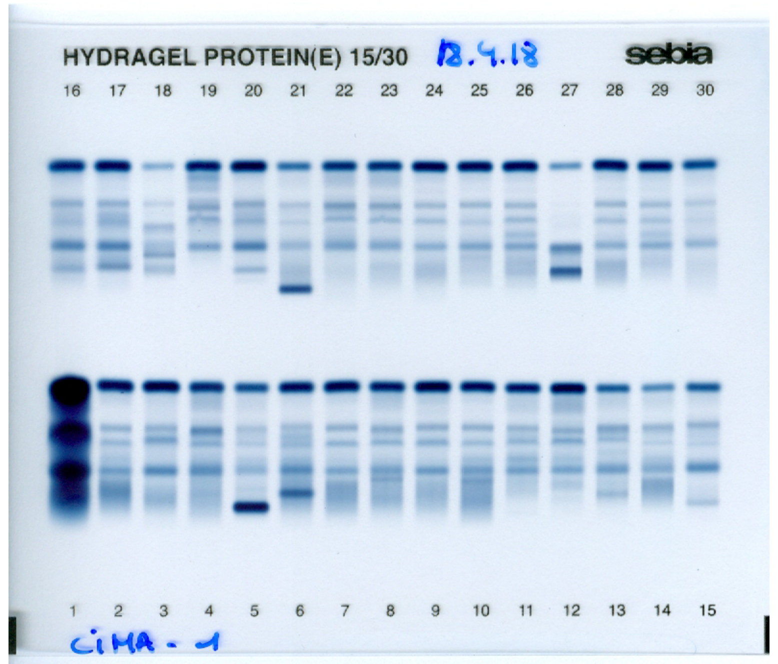

Extended Data Figure 2b
Kras-Mlcy1: sample 14

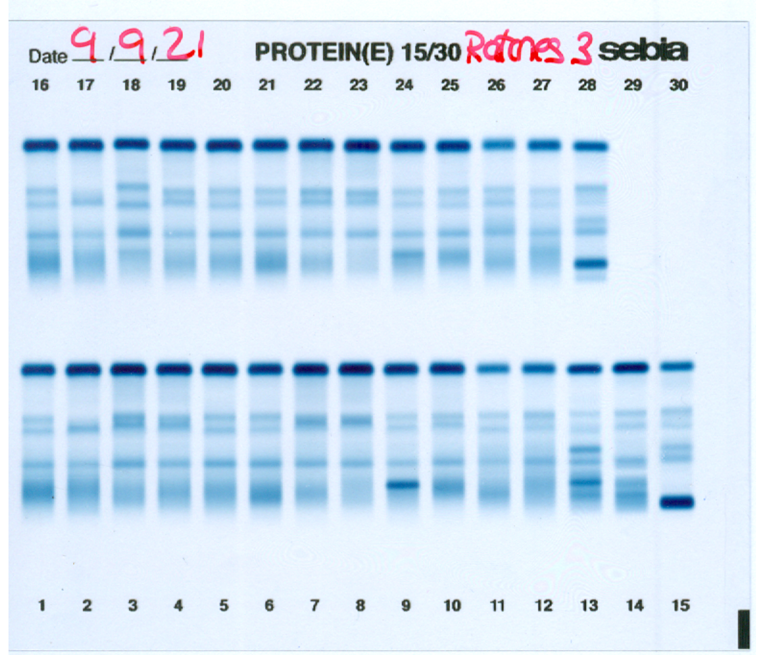

Extended Data Figure 3c
Mmset-Icy1: sample 15

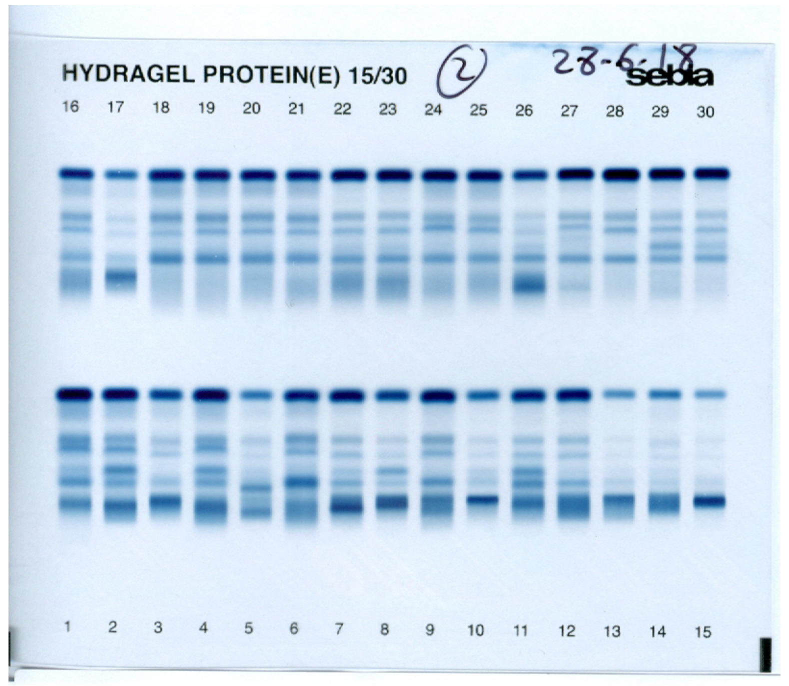

Extended Data Figure 2a
Kras/Trp53-Blcy1: sample 26

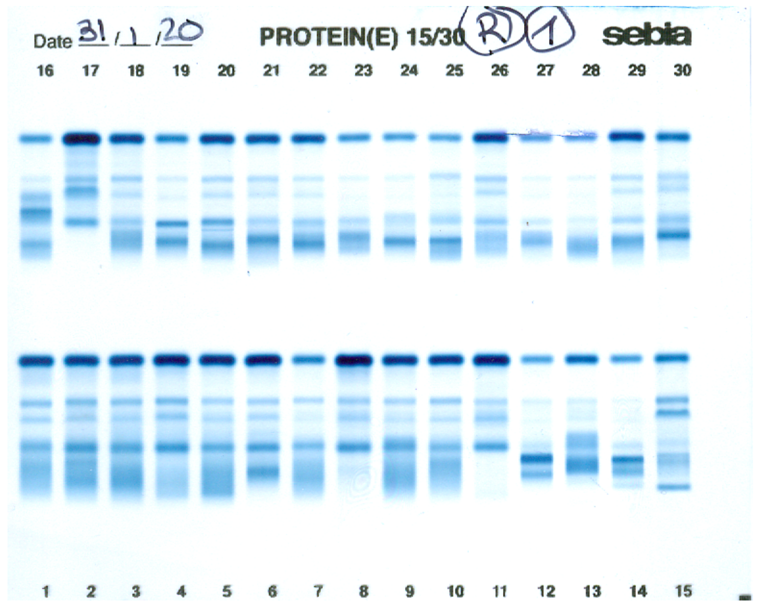

Extended Data Figure 2a
Trp53-BMcy1: sample 14

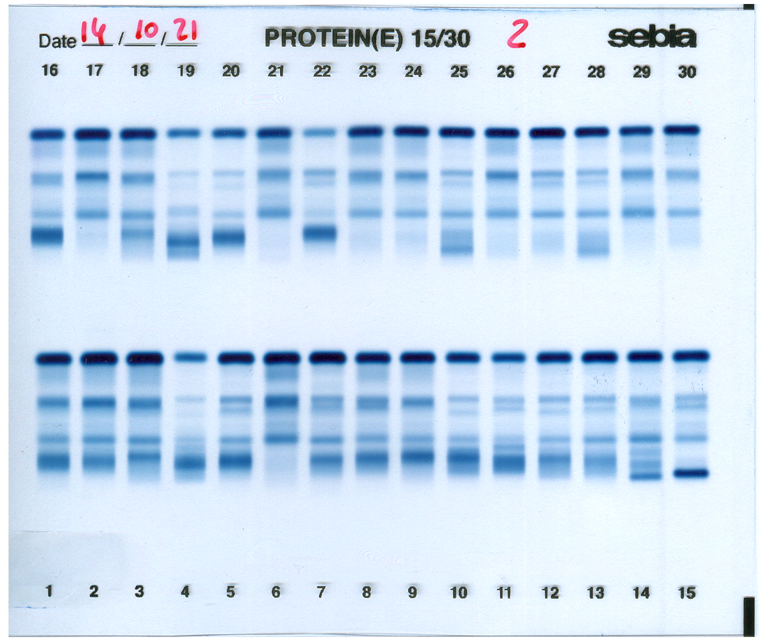

Extended Data Figure 3c
Mmsetcy1: sample 30
Mmset-Mcy1: sample 15
