# Supplementary material for: Preclinical models for prediction of immunotherapy outcomes and immune evasion mechanisms in genetically heterogeneous multiple myeloma
Source: Nat Med. 2023 Mar 16;29(3):632–45. doi: 10.1038/s41591-022-02178-3 (PMC10033443; doi:10.1038/s41591-022-02178-3)

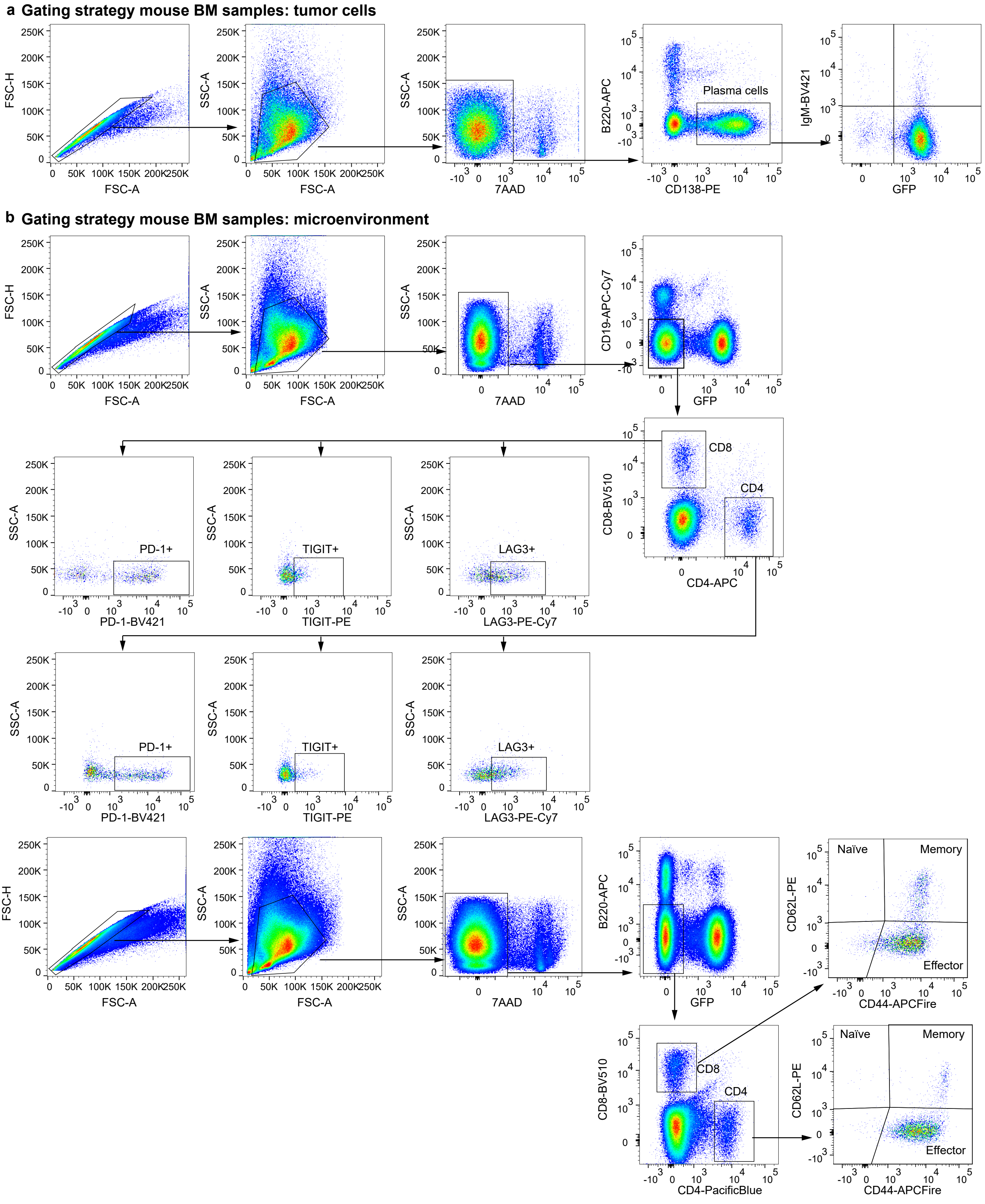
a Gating strategy mouse BM samples: tumor cells
FSC-H
FSC-A
SSC-A
7AAD
B220-APC
CD138-PE
Plasma cells
IgM-BV421
GFP
b Gating strategy mouse BM samples: microenvironment
CD19-APC-Cy7
CD8-BV510
CD4-APC
CD8
CD4
PD-1+
PD-1-BV421
TIGIT+
TIGIT-PE
LAG3+
LAG3-PE-Cy7
CD62L-PE
CD44-APCFire
Naïve
Memory
Effector
CD4-PacificBlue

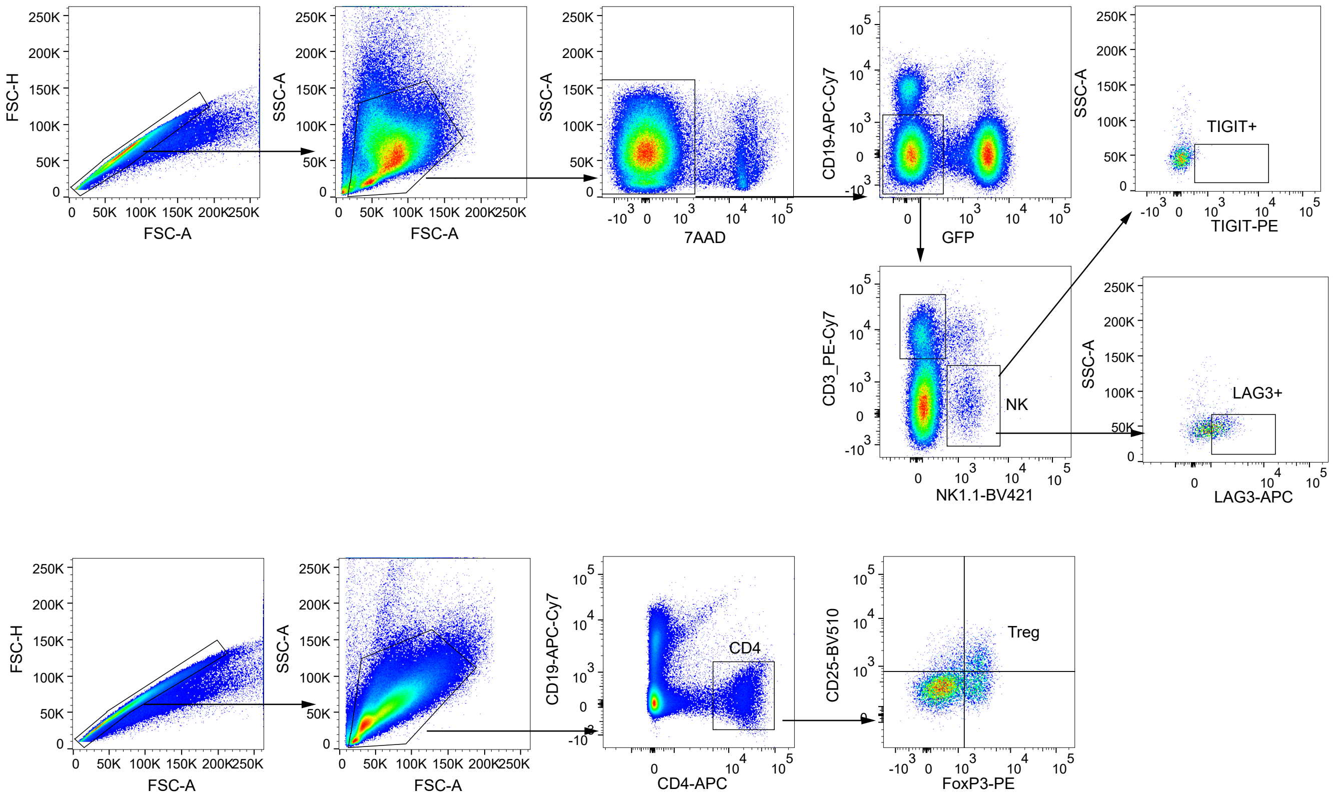

c Gating strategy mouse BM samples: tumor cells and T cells

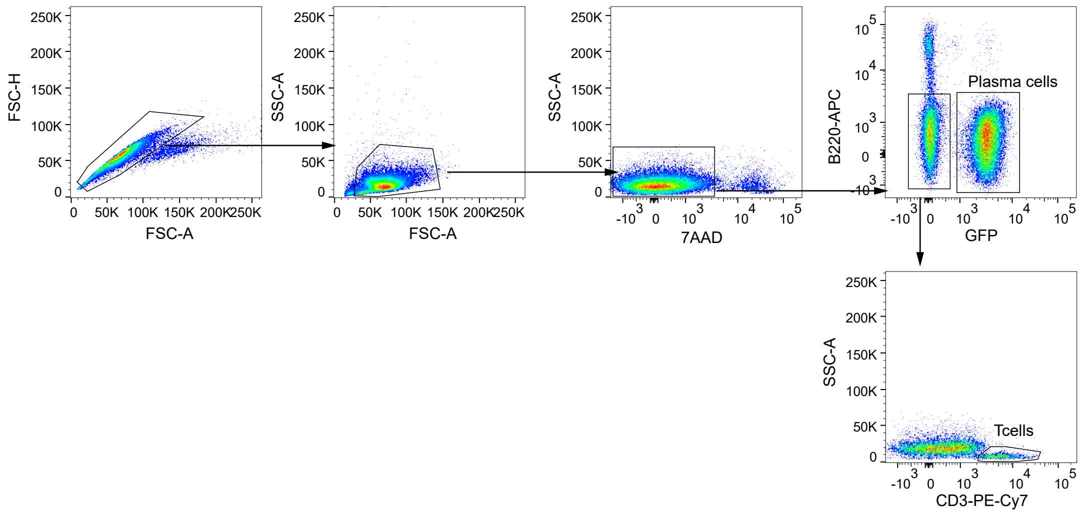

Supplement: Supplementary file 6 — Gating strategy for cytometry. [file 41591_2022_2178_MOESM6_ESM.pdf]
