# Supplementary material for: Preclinical models for prediction of immunotherapy outcomes and immune evasion mechanisms in genetically heterogeneous multiple myeloma
Source: Nat Med. 2023 Mar 16;29(3):632–45. doi: 10.1038/s41591-022-02178-3 (PMC10033443; doi:10.1038/s41591-022-02178-3)

Figure 2f MYC

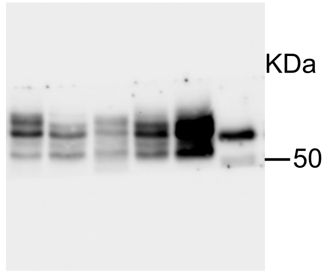

Figure 2f Tubulin

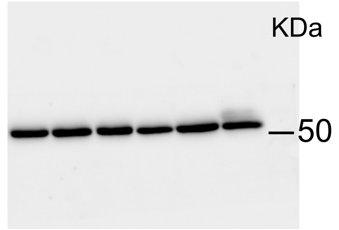

Figure 2g MYC

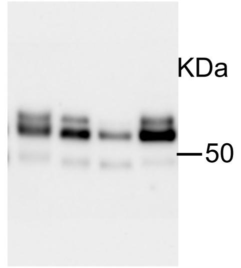

Figure 2g Tubulin

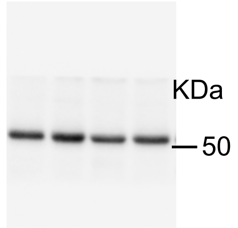

Figure 2k MYC

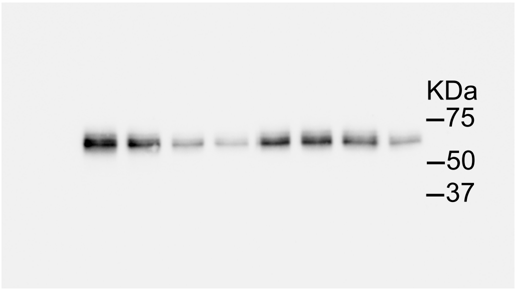

Figure 2k Actin

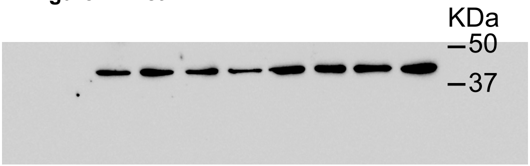

Figure 2k MYC JJN3

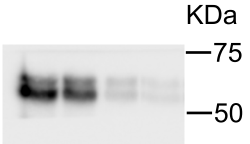

Figure 2k Actin JJN3

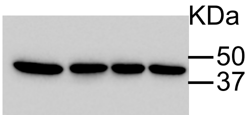

Figure 2k MYC RPMI8226

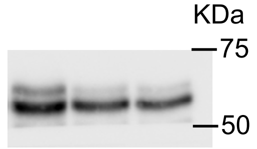

Figure 2k Actin RPMI8226

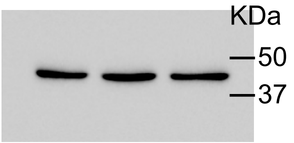

Figure 3c pERK mouse

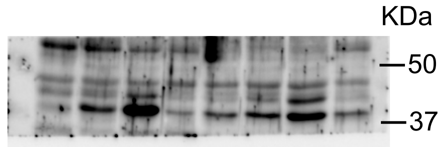

Figure 3c ERK mouse

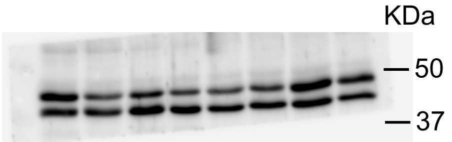

Figure 3c Tubulin mouse

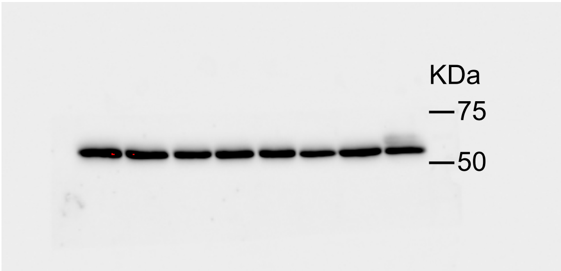

Figure 3c pERK human

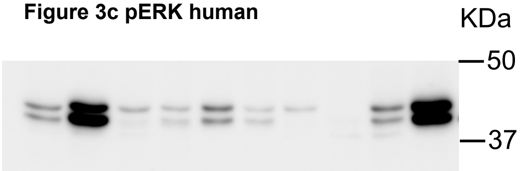

Figure 3c ERK human

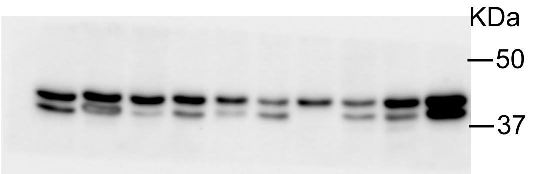

Figure 3c Tubulin human

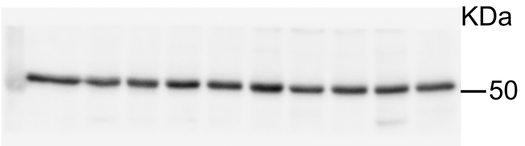

Figure 3d pERK mouse

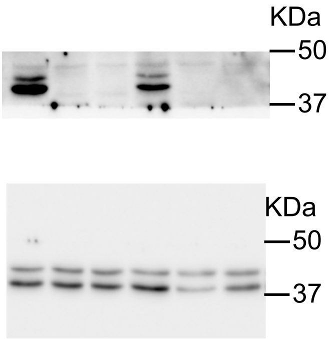

Figure 3d MYC mouse

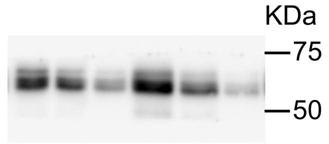

Figure 3d Actin mouse

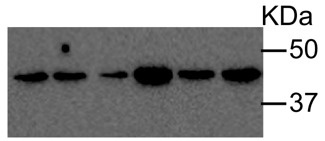

Figure 3d pERK U266

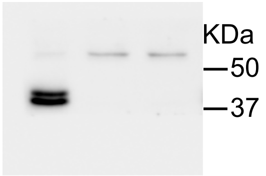

Figure 3d ERK U266

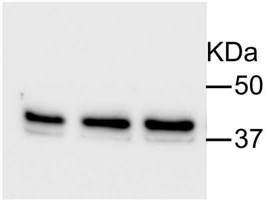

Figure 3d MYC U266

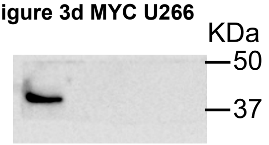

Figure 3d Actin U266

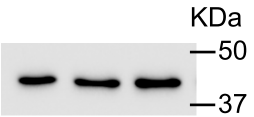

Figure 3d pERK JJN3

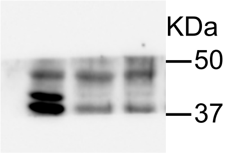

Figure 3d ERK JJN3

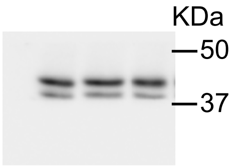

Figure 3d MYC JJN3

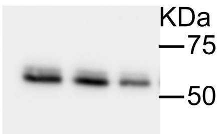

Figure 3d Actin JJN3

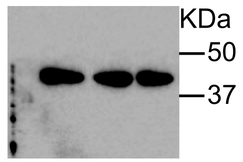

Figure 3f pMYC-S62 MM9275

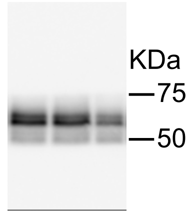

Figure 3f MYC MM9275

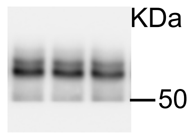

Figure 3f Actin MM9275

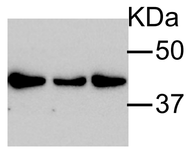

Figure 3f pMYC-S62 MM5080

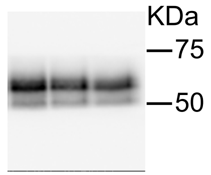

Figure 3f MYC MM5080

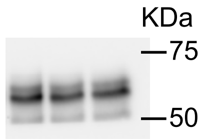

Figure 3f Actin MM5080

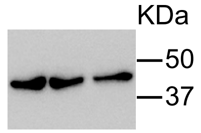

Figure 3f pMYC-S62 H929

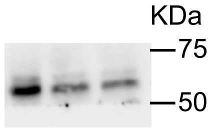

Figure 3f MYC H929

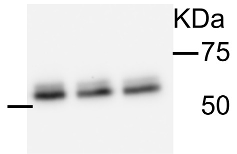

Figure 3f Actin H929

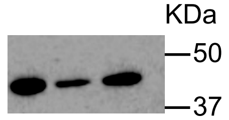

Figure 3f pMYC-S62 JJN3

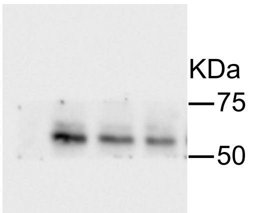

Figure 3f MYC JJN3

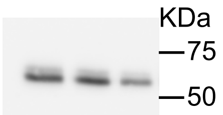

Figure 3f Actin JJN3

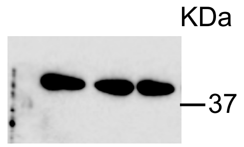

Supplement: Supplementary file 5 — Unprocessed western blot images corresponding to blots in Figs. 2 and 3. [file 41591_2022_2178_MOESM5_ESM.pdf]
